# Stereo- and Enantioselective Syntheses of 1,2-Oxaborinan-3-enes and δ-Boryl-Substituted Homoallylic Alcohols

**DOI:** 10.1021/acs.orglett.4c03755

**Published:** 2024-11-19

**Authors:** Zheye Zhang, Ming Chen

**Affiliations:** Department of Chemistry, Virginia Tech, Blacksburg, Virginia 24061, United States

## Abstract

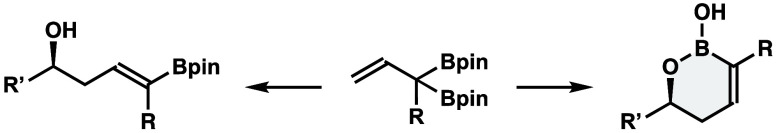

Stereo- and enantioselective syntheses of 1,2-oxaborinan-3-enes and δ-boryl-substituted homoallylic alcohols are reported. We developed a practical approach to synthesize α-boryl-substituted allylboronate. This reagent was utilized to generate α,α-disubstituted allylboronates, and such reagents cannot be accessed via the Pd-catalyzed alkene isomerization approach. Chiral Brønsted-acid-catalyzed aldehyde addition with these reagents gave 1,2-oxaborinan-3-enes with excellent stereo- and enantioselectivities. Lewis-acid-catalyzed aldehyde addition also worked well, affording δ-boryl-substituted homoallylic alcohols with high stereoselectivities. The enantioselective variant of the reaction was achieved via a chiral Brønsted-acid-catalyzed aldehyde addition and Pd-catalyzed alkene isomerization approach.

Homoallylic alcohols with an internal alkene unit (e.g., **I**–**IV**) are common structural motifs in numerous bioactive natural products (Figure 1). For instance, biselyngbyolide B and mangrolide D contain a δ-substituted *E*-homoallylic alcohol unit, while a *Z*-homoallylic alcohol is embedded in the carbon framework of epothilone D and linieodolide B.^1^ Owing to their biological importance, the development of methods that could allow for stereo- and enantioselective syntheses of these δ-substituted homoallylic alcohols would be valuable.

**Figure 1:**
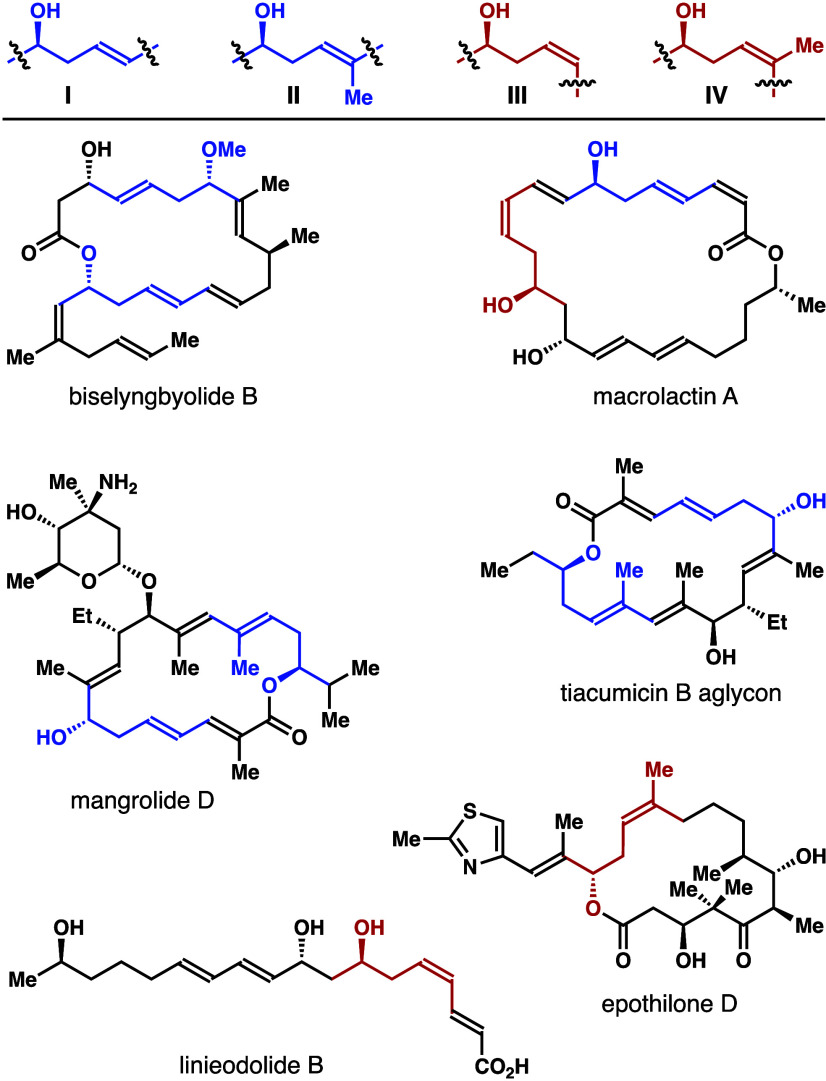
Selected natural products containing an (*E*)- or (*Z*)-δ-substituted-homoallylic alcohol structural motif.

Enantioselective aldehyde addition with allylation reagents has been one of the most adopted methods to generate enantioenriched homoallylic alcohols. Over the past few decades, many classes of allylation reagents have been developed to produce highly enantioenriched homoallylic alcohols with a terminal alkene.^2,3^ By contrast, stereoselective syntheses of the analogous homoallylic alcohols with a stereodefined internal alkene (e.g., **I**–**IV**) remain underdeveloped.^4^ One potential approach that could permit access to homoallylic alcohols **I**–**IV** is asymmetric aldehyde addition with α-boryl-substituted allylation reagents. However, the development of such allylation reagents has not attracted much attention until recently.^5^ As shown in Scheme 1, the Murakami group reported that α-boryl allylboronate **1a** can be generated *in situ* from vinyl boronate **A**.^5f^ The transient allylboronate intermediate **1a** underwent asymmetric aldehyde addition and Pd-catalyzed alkene isomerization to give enantioenriched δ-boryl-substituted homoallylic alcohols **2** with high *E*-selectivities. The vinyl Bpin group in **2** can undergo subsequent transformations to give homoallylic alcohols **I** with an internal *E*-alkene unit. However, a similar approach to enantioenriched homoallylic alcohols **II**–**IV** via **1** is not available currently.

**Scheme 1:**
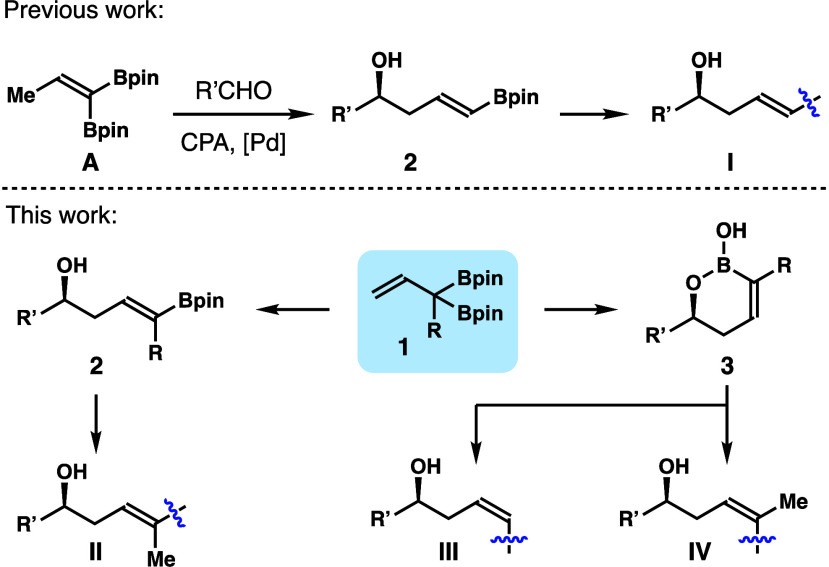
Approaches to δ-Substituted Homoallylic Alcohols

With our ongoing research interest in asymmetric synthesis with organoboron compounds,^6^ we report herein the development of α-boryl-substituted allylboronate and α,α-disubstituted allylboron reagents **1** (Scheme 1). Stereo- and enantioselective aldehyde addition with the reagents formed allylated products **2** and **3** with high selectivities. The boryl group in **2** and **3** allows for further transformations to generate alcohols **II**–**IV**.

The initial goal was to identify a practical protocol to synthesize reagents **1**. Inspired by the pioneering work from the Matteson group, we envisaged α-boryl allylboronate **1a** should be readily accessible from 1,1-bisboryl-chloromethane **4** via a Matteson homologation approach.^7^ In the event, boronate **4** was prepared and treated with vinyl Grignard **5**.^8^ The resulting ate complex **6** underwent a 1,2-vinyl shift to give α-boryl allylboronate **1a** in 80% yield (Scheme 2). The reaction is highly reproducible and can be conveniently conducted in a multigram-scale, affording **1a** in 68% yield.

**Scheme 2:**
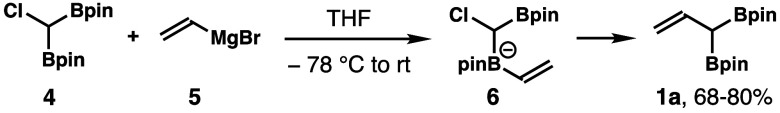
Synthesis of α-Boryl Allylboronate **1a**

With a robust approach to reagent **1a**, we next explored methods to generate the corresponding α,α-disubstituted allylboronates **1b** and **1c**. As shown in Scheme 3, α-boryl allylboronate **1a** was treated with LiTMP at 0 °C. Owing to the electronic stabilization provided by the neighboring two boron atoms, it is anticipated that the resulting anion should be localized at the carbon atom α to the boryl groups to give intermediate **7** (the boron α-anion effect).^9^ Subsequent alkylation with MeI afforded **1b** in moderate yield. Similarly, reagent **1c** was produced in 45% yield from EtI.

**Scheme 3:**
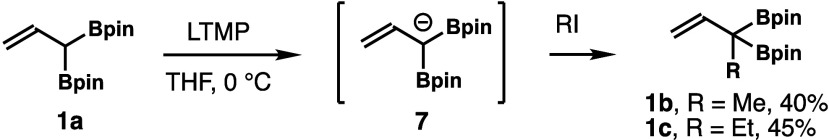
Syntheses of α,α-Disubstituted Allylboronates

With boron reagents **1** in hand, aldehyde addition studies with reagent **1a** were conducted next. As shown in Scheme 4, **1a** was treated with a broad spectrum of aldehydes at ambient temperature. The reactions worked well with aromatic aldehydes, α,β-unsaturated aldehydes, and aldehydes containing a heterocycle, affording products **3a**–**j** in 74–96% yields with 11:1 to >20:1 *Z*-selectivities. Aliphatic aldehydes also reacted with **1a** to give **3k**,**l** in 77–80% yields, albeit with lower *Z*-selectivities (5:1).

**Scheme 4:**
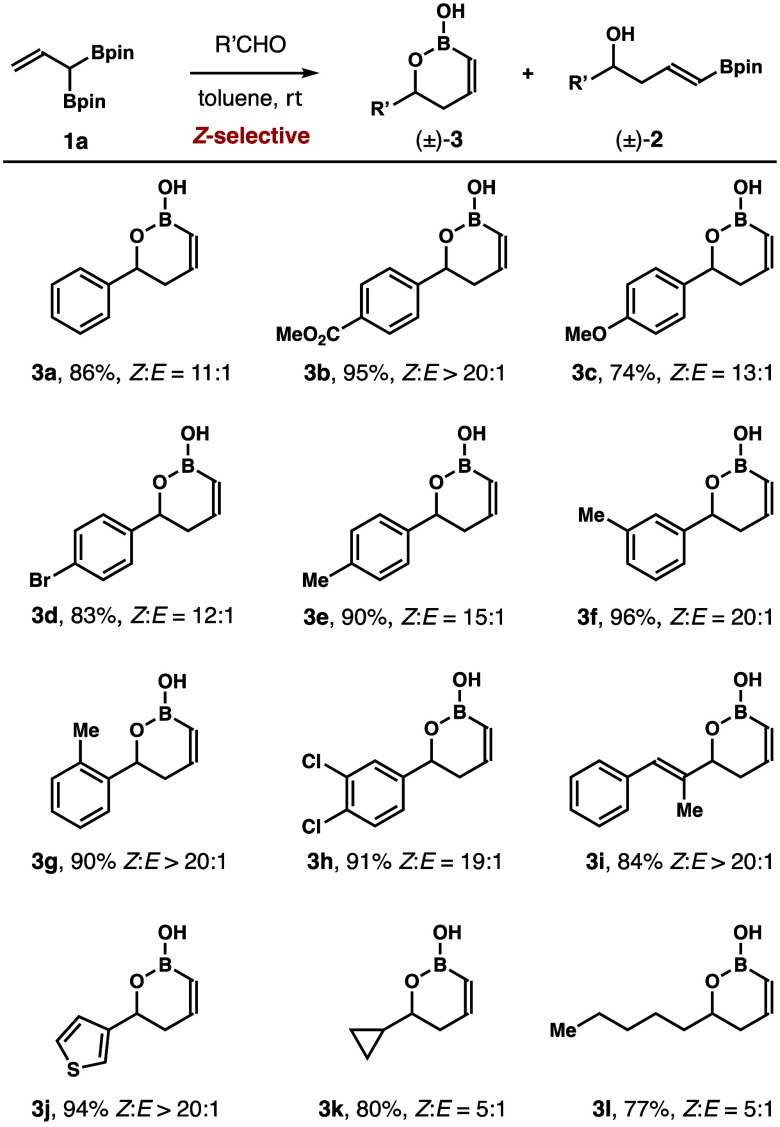
Aldehyde Scope for the Reactions with Reagent **1a**– Boronate **1a** (0.13 mmol, 1.3 equiv), aldehyde (0.1 mmol, 1.0 equiv), toluene, rt. *E*/*Z*-selectivities were determined by ^1^H NMR analysis of the crude reaction products. Yields of isolated products are listed.

The observed *Z*-selectivity was rationalized by the transition state analyses shown in Scheme 5. In transition state **TS**-**1** that leads to product (±)-**3**, the α-Bpin group of reagent **1a** is oriented in a pseudoaxial position with minimal steric interaction. By contrast, in transition state **TS**-**2** that forms product (±)-**2**, the α-Bpin group is placed in a pseudoequatorial position, and unfavorable gauche interactions are developed between the α-Bpin group and the pinacol group on the chelating boron atom. The formation of product (±)-**2** is therefore disfavored.

**Scheme 5:**
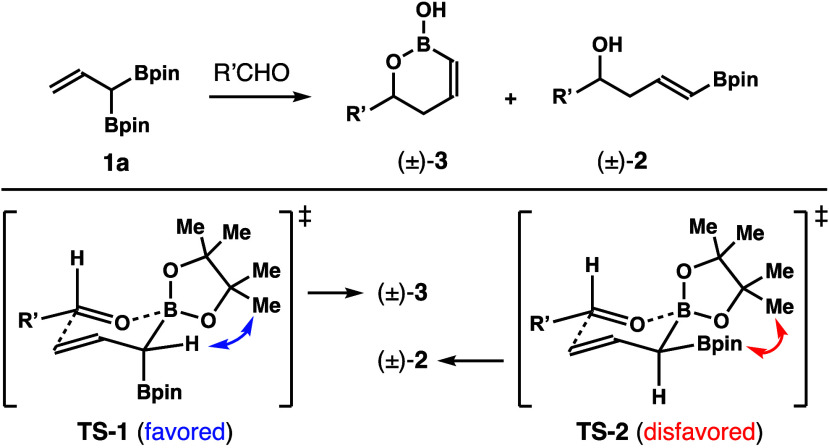
Transition State Analyses

A variety of unsaturated boronates have been shown to participate in asymmetric aldehyde addition in the presence of a chiral Brønsted acid catalyst, affording products with excellent enantioselectivities.^10−13^ To evaluate whether enantioenriched 1,2-oxaborinan-3-enes **3** could be generated from reagents **1**, chiral phosphoric acid (*S*)-**A**-catalyzed aldehyde addition studies were performed. As shown in Scheme 6, the reactions were conducted with 5 mol % (*S*)-**A** as the catalyst in toluene at −45 °C. Several representative aldehydes reacted with reagent **1a** under the conditions to generate allylated products **3d**–**i** in 89–96% yield with >20:1 *Z*-selectivities and 90–99% ee. Asymmetric allylation with reagents **1b** and **1c** was also conducted. The reactions with *para*-bromo-benzaldehyde under the same conditions exclusively gave *E*-products **3m** and **3n** in 82% yield with 99% ee and 96% yield with 99% ee, respectively. The sense of asymmetric induction in these reactions is consistent with prior reports.^13^

**Scheme 6:**
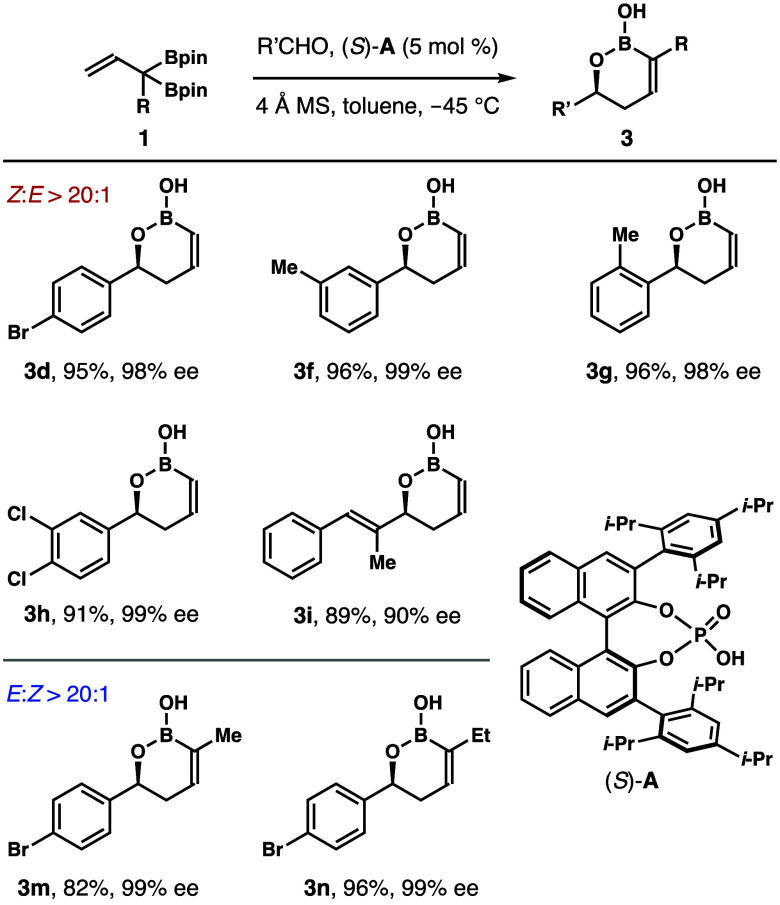
Chiral Phosphoric-Acid-Catalyzed Asymmetric Aldehyde Addition with Reagents **1**– Reaction conditions: boronate **1** (0.13 mmol, 1.3 equiv), aldehyde (0.1 mmol, 1.0 equiv), (*S*)-**A** (5 mol %), 4 Å molecular sieves (50 mg), toluene, −45 °C. *E*/*Z*-selectivities were determined by ^1^H NMR analysis of the crude reaction products. Yields of isolated products are listed. Enantioselectivities were determined by HPLC analysis of the derivatives obtained from Suzuki coupling with PhI.

Studies on Lewis-acid-catalyzed aldehyde addition with reagents **1** were carried out next.^14^ In the presence of 20 mol % BF_3_·OEt_2_, the reaction of reagents **1a**–**c** with benzaldehyde at −78 °C generated the racemic product (±)-**2a**–**c** in 80–91% yields with high stereoselectivities (Scheme 7). Similar results were achieved with *para*-Cl-PhCHO, affording products **2d**,**e** in 81–84% yields again with high stereoselectivities.

**Scheme 7:**
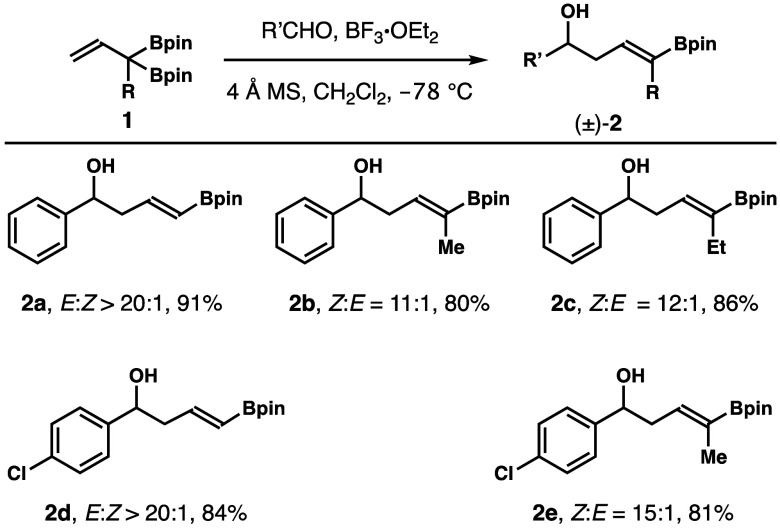
Lewis-Acid-Catalyzed Aldehyde Addition with Reagents **1**– Reaction conditions: boronate **1** (0.13 mmol, 1.3 equiv), R′CHO (0.1 mmol, 1.0 equiv), BF_3_·OEt_2_ (20 mol %), 4 Å molecular sieves (50 mg), CH_2_Cl_2_, −78 °C. *E*/*Z*-selectivities were determined by ^1^H NMR analysis of crude reaction products. Yields of isolated products are listed.

To obtain enantioenriched product **2**, we investigated a one-pot chiral Brønsted-acid-catalyzed aldehyde addition and Pd-catalyzed alkene isomerization strategy that was pioneered by the Murakami group.^5a^ As shown in Scheme 8, the reaction of *para*-chlorobenzaldehyde with reagent **1a** was conducted with 5 mol % acid (*S*)-**A** and 5 mol % [Pd(μ-Br)P^*t*^Bu_3_]_2_ as the catalyst system.^15^ Gratifyingly, product **2d** was obtained in 75% yield with excellent stereo- and enantioselectivity.

**Scheme 8:**
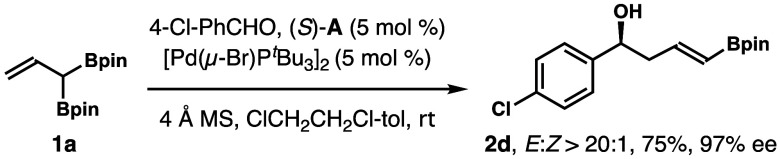
Enantioselective Synthesis of δ-Boryl-Substituted (*E*)-Homoallylic Alcohol

The derivatization studies with products obtained from reagents **1** were conducted next. To generate structural motifs that are closely related to the natural products shown in Figure 1, the studies were focused on Pd-catalyzed Suzuki couplings. As shown in Scheme 9, **3a** underwent coupling with *E*- and *Z*-vinyl iodides, **8** and **9**, to give *Z*,*E*-diene **11** and *Z*,*Z*-diene **12** in 71 and 75% yield, respectively. Coupling of **2a** with vinyl iodide **9** gave *E*,*Z*-diene **13** in a 78% yield. Diene **14** was obtained in 74% yield from **2a** and vinyl bromide **10**. The reaction of vinyl iodide **9** and boronate **2b** occurred to give product **15** in 80% yield. Boronate **2b** reacted with vinyl bromide **10** to deliever diene **16** in 76% yield. Finally, boronates **2d** and **2e** coupled with *E*-vinyl iodide **8**, affording dienes **17** and **18** in 74–79% yields.

**Scheme 9:**
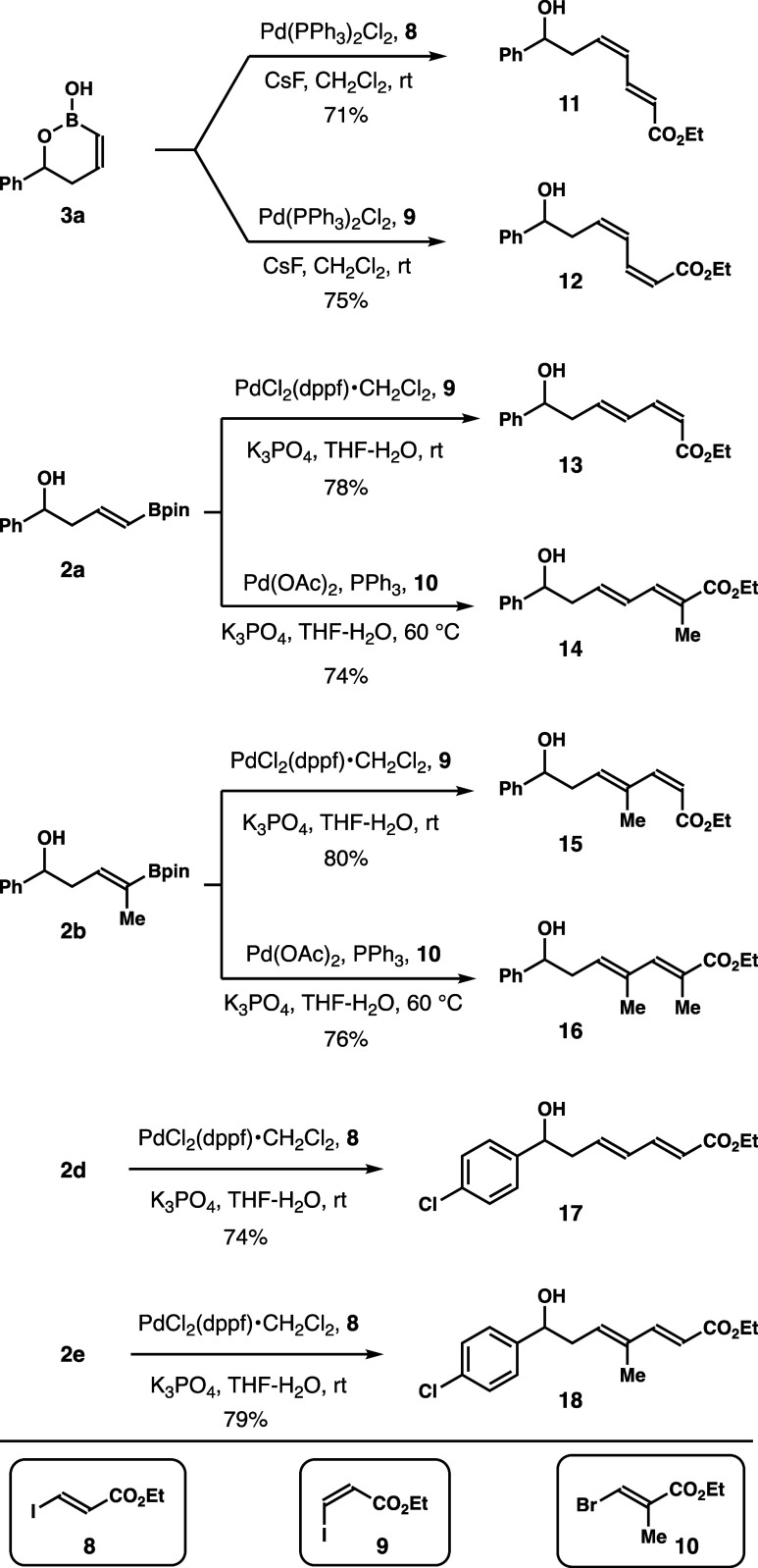
Product Derivatization Studies

In summary, we developed a simple protocol to synthesize α-boryl-substituted allylboronate **1a**. α,α-Disubstituted boronate reagents **1b** and **1c** were generated via alkylation from **1a**. It is worth noting that reagents **1b** and **1c** have not been synthesized in any prior studies. While reagent **1a** can be generated *in situ* via an alkene isomerization approach, the same approach is not applicable to α,α-disubstituted boron reagents **1b** and **1c**. Chiral Brønsted-acid-catalyzed aldehyde addition with reagents **1** gave 1,2-oxaborinan-3-enes **3** with excellent stereo- and enantioselectivities. Lewis-acid-catalyzed aldehyde addition with **1** afforded racemic δ-boryl-substituted homoallylic alcohols **2** with high stereoselectivities. Enantioenriched homoallylic alcohols **2** can be generated via a one-pot chiral Brønsted-acid-catalyzed aldehyde addition and Pd-catalyzed alkene isomerization approach. The boryl group in products **2** and **3** allows for further transformations to generate homoallylic alcohols with a stereodefined internal alkene. Synthetic applications are currently under investigation.

## Data Availability

The data underlying this study are available in the published article and its Supporting Information.

## Supporting Information Available

^1^H and ^13^C NMR spectra for all new compounds (PDF)
