# Supplementary material for: Stereo- and Enantioselective Syntheses of 1,2-Oxaborinan-3-enes and δ-Boryl-Substituted Homoallylic Alcohols
Source: Org Lett. 2024 Nov 19;26(47):10102–7. doi: 10.1021/acs.orglett.4c03755 (PMC11613688; doi:10.1021/acs.orglett.4c03755)
Supplement: Supplementary file 1 — ol4c03755_si_001.pdf [file ol4c03755_si_001.pdf]

Stereo- and Enantioselective Syntheses of 1,2-Oxaborinan-3-enes and δ-Boryl-Substituted Homoallylic Alcohols

*Zheye Zhang and Ming Chen**

Department of Chemistry, Virginia Tech

E-mail: mzc0102@vt.edu

Supporting Information: Experimental Procedures, Tabulated Spectroscopic Data, ^{1}H and ^{13}C Spectra of New Compounds

General Experimental Details. All reaction solvents were purified before use. Dichloromethane, THF and toluene were purified by passing through a solvent column composed of activated A-1 alumina. Unless indicated otherwise, all reactions were conducted under an atmosphere of argon using flame-dried or oven-dried (140 °C) glassware. The term “concentrated under reduced pressure” refers to the removal of solvents and other volatile materials using a rotary evaporator with the water bath temperature below 30 °C, followed by the removal of residual solvents at high vacuum (< 0.2 mbar).

Proton nuclear magnetic resonance (^{1}H NMR) spectra were acquired on commercial instruments at 400, 500 and 600 MHz. Carbon-13 nuclear magnetic resonance (^{13}C NMR) spectra were acquired at 101, 126 and 151 MHz. The proton signal for the residual non-deuterated solvent (δ 7.26 for $CHCl_3$) was used as an internal reference for ^{1}H NMR spectra. For ^{13}C NMR spectra, chemical shifts are reported relative to the δ 77.36 resonance of $CHCl_3$. Coupling constants are reported in Hz. High-resolution mass spectra were recorded on a commercial high-resolution mass spectrometer (mass analyzer type: QTOF).

Analytical thin layer chromatography (TLC) was performed on Kieselgel 60 F254 glass plates precoated with a 0.25 mm thickness of silica gel. The TLC plates were visualized with UV light and/or by staining with Hanessian solution (ceric sulfate and ammonium molybdate in aqueous sulfuric acid) or $KMnO_4$. Column chromatography was performed using Kieselgel 60 (230-400 mesh) silica gel, typically using a 50 – 100:1 weight ratio of silica gel to crude product.

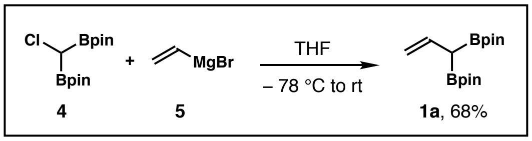

2,2'-(prop-2-ene-1,1-diyl)bis(4,4,5,5-tetramethyl-1,3,2-dioxaborolane) (1a): To a solution of boronate **4** [1] (2.42 g, 8 mmol, 1.0 equiv) in 50 mL anhydrous THF at −78 °C was slowly added vinyl magnesium bromide (0.7 M solution in THF, 12 mL, 8.4 mmol, 1.05 equiv) dropwise over 15 min. The reaction mixture was allowed to warm to ambient temperature. The reaction progress was monitored by ^{1}H NMR analysis. After complete consumption of boronate **4**, hexane (100 mL) was added to the reaction mixture. The precipitation was removed by filtration, and the filtrate was concentrated under reduced pressure to a volume about 5 mL. Hexane (50 mL) was added, and the resulting mixture was filtered. The filtrate was concentrated under reduced pressure. Purification of the crude product was performed by flash column chromatography (gradient elution with hexane and ethyl acetate, 50:1 to 10:1) to give product **1a** in 68% yield (1.60 g) as colorless oil. ^{1}H NMR (600 MHz, $CDCl_3$) δ 5.97 (ddd, *J* = 17.0, 10.0 Hz, 10.0 Hz, 1H), 4.92 (dd, *J* = 17.0, 1.8 Hz, 1H), 4.88 (dd, *J* = 9.9, 1.8 Hz, 1H), 1.85 (d, *J* = 9.9 Hz, 1H), 1.24 (s, 12H), 1.23 (s, 12H). ^{13}C NMR (151 MHz, $CDCl_3$) δ 135.4, 113.5, 83.6, 25.0, 24.9. The data was consistent with data reported in the literature. [2]

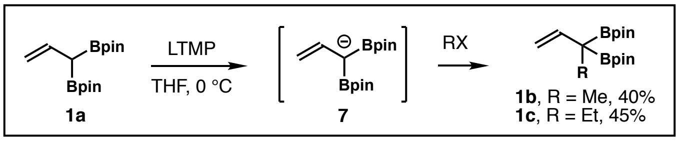

General procedure for the synthesis of allylboronates 1b-c: A round-bottomed flask equipped with a magnetic stir bar were added LTMP (1.2 mmol, 1.2 equiv) and anhydrous THF (5 mL). The mixture was cooled to 0 °C and stirred for 5 min. Then a solution of boronate **1a** (1.0 mmol, 1.0 equiv) in THF (1 mL) was added and the resulting mixture was stirred at 0 °C for 30 min. A solution of alkyl halide (1.5 mmol, 1.5 equiv) in THF (1 mL) was added. The reaction mixture was stirred at 0 °C. After completion of the reaction (~1 h), a saturated NH_4Cl solution (5 mL) was added, and the mixture was allowed to warm to ambient temperature. The organic layer was separated, and the aqueous layer was extracted with Et_2O (5 mL x 3). The combined organic extracts were dried over anhydrous sodium sulfate, filtered, and concentrated under reduced pressure. Purification of the crude product by flash column chromatography provided product **1**.

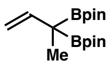

2,2'-(but-3-ene-2,2-diyl)bis(4,4,5,5-tetramethyl-1,3,2-dioxaborolane) (1b) Prepared according to the general procedure with MeI. The crude mixture was purified by flash column chromatography (gradient elution with hexane and ethyl acetate, 30:1 to 10:1) to give compound **1b** in 40% yield (123 mg) as colorless oil. ^{1}H NMR (600 MHz, $CDCl_3$) δ 6.27 (dd, J = 17.4, 10.6 Hz, 1H), 4.90 (dd, J = 10.6, 1.6 Hz, 1H), 4.83 (dd, J = 17.4, 1.6 Hz, 1H), 1.22 (s, 12H), 1.21 (s, 12H), 1.20 (s, 3H). ^{13}C NMR (151 MHz, $CDCl_3$) δ 141.9, 109.1, 83.6, 25.0, 24.8, 14.1. HRMS (ESI$^+$): m/z for $C_{16}H_{30}B_2O_4Na$ $[M+Na]^+$ calcd. 331.2228, found: 331.2232.

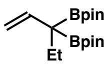

2,2'-(pent-1-ene-3,3-diyl)bis(4,4,5,5-tetramethyl-1,3,2-dioxaborolane) (1c) Prepared according to the general procedure with EtI. The crude mixture was purified by flash column chromatography (gradient elution with hexane and ethyl acetate, 30:1 to 10:1) to give compound **1c** in 45% yield (145 mg) as colorless oil. ^{1}H NMR (600 MHz, $CDCl_3$) δ 6.07 (dd, J = 17.7, 10.8 Hz, 1H), 5.02 (dd, J = 10.7, 1.7 Hz, 1H), 4.97 (dd, J = 17.6, 1.7 Hz, 1H), 1.81 (q, J = 7.3 Hz, 2H), 1.22 (s, 24H), 0.91 (t, J = 7.3 Hz, 3H). ^{13}C NMR (151 MHz, $CDCl_3$) δ 139.6, 112.0, 83.5, 25.0, 24.9, 23.1, 12.2. HRMS (ESI$^+$): m/z for $C_{17}H_{32}B_2O_4Na$ $[M+Na]^+$ calcd. 345.2384, found: 345.2380.

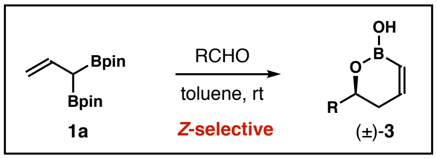

General procedure for the syntheses of (±)-3: To a reaction vial containing a stirring bar were added the aldehyde substrate (0.1 mmol) and toluene (0.2 mL). Allylboronate **1a** (0.13 mmol, 1.3 equiv) in toluene (0.1 mL) was added slowly to the reaction mixture. The mixture was allowed to stir at ambient temperature and the reaction progress was monitored by ^{1}H NMR analysis. After complete consumption of the aldehyde, the reaction mixture was filtered through a pad of silica gel. The filtrate was concentrated under reduced pressure. Purification of the crude product was performed by flash column chromatography to give product (±)-**3**.

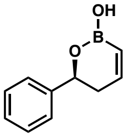

***rac*-6-phenyl-5,6-dihydro-2H-1,2-oxaborinin-2-ol (3a)** Prepared according to the general procedure. The crude mixture was purified by flash column chromatography (gradient elution with hexane and ethyl acetate, 15:1 to 5:1) to give compound **3a** in 86% yield (15 mg, Z:E = 11:1) as colorless oil. A 1-mmol scale reaction was conducted with PhCHO (106 mg, 1 mmol), and product **3a** was obtained in

81% yield (141 mg) with 10:1 *Z*-selectivity. ^{1}H NMR (600 MHz, $CDCl_3$) δ 7.36 – 7.40 (m, 4H), 7.29 – 7.32 (m, 1H), 6.98 (m, 1H), 5.83 (dd, *J* = 12.1, 2.5 Hz, 1H), 5.15 (dd, *J* = 11.0, 5.0 Hz, 1H), 4.12 (s, 1H), 2.43 – 2.52 (m, 2H). ^{13}C NMR (151 MHz, $CDCl_3$) δ 150.9, 143.0, 128.8, 128.0, 126.1, 76.0, 37.1. The data was consistent with data reported in the literature.[3]

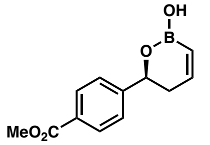

***rac*-methyl-4-(2-hydroxy-5,6-dihydro-2H-1,2-oxaborinin-6-yl)-benzoate** (**3b**) Prepared according to the general procedure. The crude mixture was purified by flash column chromatography (gradient elution with hexane and ethyl acetate, 15:1 to 3:1) to give compound **3b** in 95% yield (22 mg, *Z*:*E* > 20:1) as white solid. ^{1}H NMR (600 MHz, $CDCl_3$) δ 8.04 (d, *J* = 8.4 Hz, 2H), 7.46 (d, *J* = 8.0 Hz, 2H), 6.97 (ddd, *J* = 12.2, 5.7, 2.2 Hz, 1H), 5.83 (dd, *J* = 12.1, 2.6 Hz, 1H), 5.20 (dd, *J* = 12.0, 4.1 Hz, 1H), 4.27 (s, 1H), 3.92 (s, 3H), 2.50 – 2.54 (m, 1H), 2.37 – 2.43 (m, 1H). ^{13}C NMR (151 MHz, $CDCl_3$) δ 167.3, 150.6, 148.1, 130.1, 129.6, 125.9, 75.5, 52.5, 36.9. HRMS (ESI$^+$): m/z for $C_{12}H_{14}BO_4$ $[M+H]^+$ calcd. 233.0985, found: 233.0989.

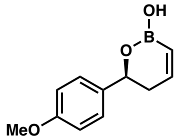

***rac*-6-(4-methoxyphenyl)-5,6-dihydro-2H-1,2-oxaborinin-2-ol (3c)** Prepared according to the general procedure. The crude mixture was purified by flash column chromatography (gradient elution with hexane and ethyl acetate, 15:1 to 3:1) to give compound **3c** in 74% yield (15 mg, *Z*:*E* = 13:1) as colorless oil. ^{1}H NMR (600 MHz, $CDCl_3$) δ 7.32 (d, *J* = 8.7 Hz, 2H), 6.96 – 6.98 (m, 1H), 6.90 (d, *J* = 8.7 Hz, 2H), 5.80 – 5.82 (m, 1H), 5.09 (dd, *J* = 9.9, 6.1 Hz, 1H), 4.08 (s, 1H), 3.81 (s, 3H), 2.44 – 2.47 (m, 2H). ^{13}C NMR (151 MHz, $CDCl_3$) δ 159.4, 151.0, 135.2, 127.4, 114.1, 75.7, 55.7, 37.0. HRMS (ESI$^+$): m/z for $C_{11}H_{14}BO_3$ $[M+H]^+$ calcd. 205.1036, found: 205.1035.

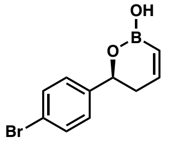

***rac*-6-(4-bromophenyl)-5,6-dihydro-2H-1,2-oxaborinin-2-ol (3d)** Prepared according to the general procedure. The crude mixture was purified by flash column chromatography (gradient elution with hexane and ethyl acetate, 15:1 to 5:1) to give compound **3d** in 83% yield (21 mg, *Z*:*E* = 12:1) as white solid. ^{1}H NMR (400 MHz, $CDCl_3$) δ 7.47 (d, *J* = 8.5 Hz, 2H), 7.25 (d, *J* = 8.3 Hz, 3H), 6.92 – 6.96 (m, 1H), 5.80 (ddd, *J* = 12.1, 2.7, 0.8 Hz, 1H), 5.09 (dd, *J* = 11.5, 4.5 Hz, 1H), 4.11 (s, 1H), 2.44 – 2.50 (m, 1H), 2.33 – 2.43 (m, 1H). ^{13}C NMR (151 MHz, $CDCl_3$) δ 150.7, 142.1, 131.9, 127.8, 121.7, 75.3, 37.0. HRMS (ESI$^+$): m/z for $C_{10}H_{11}BBrO_2$ $[M+H]^+$ calcd. 253.0035, found: 253.0037.

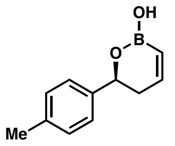

***rac*-6-(p-tolyl)-5,6-dihydro-2H-1,2-oxaborinin-2-ol (3e)** Prepared according to the general procedure. The crude mixture was purified by flash column chromatography (gradient elution with hexane and ethyl acetate, 15:1 to 5:1) to give compound **3e** in 90% yield (17 mg, *Z*:*E* = 15:1) as colorless oil. ^{1}H NMR (600 MHz, $CDCl_3$) δ 7.28 (d, *J* = 8.0 Hz, 2H), 7.18 (d, *J* = 8.0 Hz, 2H), 6.95 – 6.99 (m, 1H), 5.81 (ddd, *J* = 12.1, 1.6, 1.6 Hz, 1H), 5.11 (dd, *J* = 9.4, 6.6 Hz, 1H), 4.08 (s, 1H), 2.45 – 2.47 (m, 2H), 2.35 (s, 3H). ^{13}C NMR (151 MHz, $CDCl_3$) δ 151.0, 140.0, 137.7, 129.4, 126.1, 75.9, 37.0, 21.5. HRMS (ESI^{+}): m/z for $C_{11}H_{14}BO_2$ $[M+H]^{+}$ calcd. 189.1087, found: 189.1090.

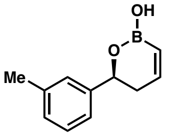

***rac*-6-(m-tolyl)-5,6-dihydro-2H-1,2-oxaborinin-2-ol (3f)** Prepared according to the general procedure. The crude mixture was purified by flash column chromatography (gradient elution with hexane and ethyl acetate, 15:1 to 5:1) to give compound **3f** in 96% yield (18 mg, *Z*:*E* = 20:1) as yellow oil. ^{1}H NMR (600 MHz, $CDCl_3$) δ 7.24 – 7.27 (m, 1H), 7.22 (s, 1H), 7.17 (d, *J* = 7.7 Hz, 1H), 7.11 (d, *J* = 7.4 Hz, 1H), 6.96 – 6.99 (m, 1H), 5.81 – 5.83 (m, 1H), 5.11 (dd, *J* = 9.9, 6.2 Hz, 1H), 4.10 (s, 1H), 2.45 – 2.48 (m, 2H), 2.37 (s, 3H). ^{13}C NMR (151 MHz, $CDCl_3$) δ 151.0, 142.9, 138.5, 128.73, 128.69, 126.8, 123.2, 76.1, 37.1, 21.8. HRMS (ESI^{+}): m/z for $C_{11}H_{14}BO_2$ $[M+H]^{+}$ calcd. 189.1087, found: 189.1088.

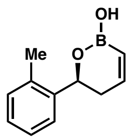

***rac*-6-(o-tolyl)-5,6-dihydro-2H-1,2-oxaborinin-2-ol (3g)** Prepared according to the general procedure. The crude mixture was purified by flash column chromatography (gradient elution with hexane and ethyl acetate, 15:1 to 5:1) to give compound **3g** in 90% yield (17 mg, *Z*:*E* > 20:1) as colorless oil. ^{1}H NMR (600 MHz, $CDCl_3$) δ 7.52 (d, *J* = 8.1 Hz, 1H), 7.25 (d, *J* = 10.5 Hz, 1H), 7.19 – 7.21 (m, 1H), 7.15 (d, *J* = 7.5 Hz, 1H), 6.98 – 7.01 (m, 1H), 5.84 (dd, *J* = 12.1, 2.5 Hz, 1H), 5.36 (dd, *J* = 11.2, 4.9 Hz, 1H), 4.09 (s, 1H), 2.38 – 2.47 (m, 2H), 2.34 (s, 3H). ^{13}C NMR (151 MHz, $CDCl_3$) δ 151.1, 140.9, 134.6, 130.8, 127.8, 126.6, 126.1, 73.1, 35.6, 19.4. HRMS (ESI^{+}): m/z for $C_{11}H_{14}BO_2$ $[M+H]^{+}$ calcd. 189.1087, found: 189.1085.

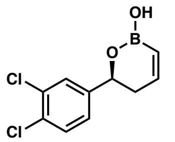

***rac*-6-(3,4-dichlorophenyl)-5,6-dihydro-2*H*-1,2-oxaborinin-2-ol (3h)** Prepared according to the general procedure. The crude mixture was purified by flash column chromatography (gradient elution with hexane and ethyl acetate, 15:1 to 5:1) to give compound **3h** in 91% yield (22 mg, *Z*:*E* = 19:1) as white solid. ^{1}H NMR (600 MHz, $CDCl_3$) δ 7.51 (d, *J* = 2.0 Hz, 1H), 7.43 (d, *J* = 8.3 Hz, 1H), 7.20 (dd, *J* = 8.3, 1.9 Hz, 1H), 6.94 – 6.97 (m, 1H), 5.82 (dd, *J* = 12.1,

2.7 Hz, 1H), 5.10 (dd, J = 12.0, 4.1 Hz, 1H), 4.14 (s, 1H), 2.47 – 2.51 (m, 1H), 2.34 – 2.40 (m, 1H). ^{13}C NMR (151 MHz, $CDCl_3$) δ 150.5, 143.3, 132.9, 131.7, 130.7, 128.1, 125.3, 74.6, 36.9. HRMS (ESI^+): m/z for $C_{10}H_{10}BCl_2O_2$ $[M+H]^+$ calcd. 243.0151, found: 243.0154.

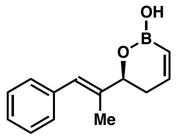

***rac*-(*E*)-6-(1-phenylprop-1-en-2-yl)-5,6-dihydro-2H-1,2-oxaborinin-2-ol** (**3i**) Prepared according to the general procedure. The crude mixture was purified by flash column chromatography (gradient elution with hexane and ethyl acetate, 15:1 to 3:1) to give compound **3i** in 84% yield (18 mg, *Z*:*E* > 20:1) as yellow oil. ^{1}H NMR (600 MHz, $CDCl_3$) δ 7.33 – 7.35 (m, 2H), 7.27 – 7.31 (m, 2H), 7.20 – 7.24 (m, 1H), 6.96 – 6.99 (m, 1H), 6.58 (s, 1H), 5.77 (dd, J = 12.1, 2.7 Hz, 1H), 4.64 (ddd, J = 11.8, 4.3, 1.0 Hz, 1H), 4.09 (s, 1H), 2.41 – 2.47 (m, 1H), 2.34 – 2.39 (m, 1H), 1.91 (d, J = 1.4 Hz, 3H). ^{13}C NMR (151 MHz, $CDCl_3$) δ 151.1, 138.4, 137.8, 129.4, 128.4, 126.9, 126.3, 79.3, 33.6, 14.3. HRMS (ESI^+): m/z for $C_{13}H_{16}BO_2$ $[M+H]^+$ calcd. 215.1243, found: 215.1247.

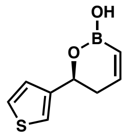

***rac*-6-(thiophen-3-yl)-5,6-dihydro-2H-1,2-oxaborinin-2-ol (3j)** Prepared according to the general procedure. The crude mixture was purified by flash column chromatography (gradient elution with hexane and ethyl acetate, 15:1 to 5:1) to give compound **3j** in 94% yield (17 mg, *Z*:*E* > 20:1) as colorless oil. ^{1}H NMR (600 MHz, $CDCl_3$) δ 7.32 (dd, J = 5.0, 3.0 Hz, 1H), 7.24 – 7.25 (m, 1H), 7.10 (dd, J = 5.0, 1.3 Hz, 1H), 6.96 – 6.98 (m, 1H), 5.80 – 5.82 (m, 1H), 5.23 (dd, J = 10.2, 5.5 Hz, 1H), 4.07 (s, 1H), 2.49 – 2.58 (m, 2H). ^{13}C NMR (151 MHz, $CDCl_3$) δ 150.7, 144.3, 126.5, 126.0, 121.3, 72.3, 35.9. HRMS (ESI^+): m/z for $C_8H_{10}BSO_2$ $[M+H]^+$ calcd. 181.0495, found: 181.0498.

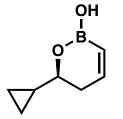

***rac*-6-cyclopropyl-5,6-dihydro-2H-1,2-oxaborinin-2-ol** (**3k**) Prepared according to the general procedure. The crude mixture was purified by flash column flash chromatography (gradient elution with hexane and ethyl acetate, 15:1 to 3:1) to give compound **3k** in 80% yield (11 mg, *Z*:*E* = 5:1) as colorless oil. ^{1}H NMR (600 MHz, $CDCl_3$) δ 6.89 – 6.91 (m, 1H), 5.70 (dd, J = 12.1, 1.4 Hz, 1H), 4.14 (s, 1H), 3.36 (ddd, J = 10.1, 8.4, 5.0 Hz, 1H), 2.31 – 2.41 (m, 2H), 1.01 – 1.07 (m, 1H), 0.56 – 0.59 (m, 1H), 0.49 – 0.54 (m, 1H), 0.38 – 0.42 (m, 1H), 0.18 – 0.22 (m, 1H). ^{13}C NMR (151 MHz, $CDCl_3$) δ 151.1, 78.7, 34.5, 16.9, 3.4, 2.3. HRMS (ESI^+): m/z for $C_7H_{12}BO_2$ $[M+H]^+$ calcd. 139.0930, found: 139.0933.

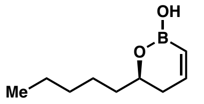

***rac*-6-pentyl-5,6-dihydro-2H-1,2-oxaborinin-2-ol** (**3l**) Prepared according to the general procedure. The crude mixture was purified by flash column chromatography (gradient elution with hexane and ethyl acetate, 15:1 to 3:1) to give compound **3l** in 77% yield (13 mg, *Z*:*E* = 5:1) as colorless oil. ^{1}H NMR (600 MHz, $CDCl_3$) δ 6.89 (ddd, *J* = 12.1, 5.3, 2.4 Hz, 1H), 5.69 (ddd, *J* = 12.0, 2.6, 0.9 Hz, 1H), 4.04 – 4.08 (m, 2H), 2.23 – 2.28 (m, 1H), 2.11 – 2.18 (m, 1H), 1.60 – 1.63 (m, 1H), 1.43 – 1.53 (m, 2H), 1.23 – 1.36 (m, 5H), 0.89 (t, *J* = 7.0 Hz, 3H). ^{13}C NMR (151 MHz, $CDCl_3$) δ 151.1, 74.0, 37.1, 34.4, 32.1, 25.4, 23.0, 14.4. HRMS (ESI^+): m/z for $C_9H_{18}BO_2$ $[M+H]^+$ calcd. 169.1400, found: 169.1400.

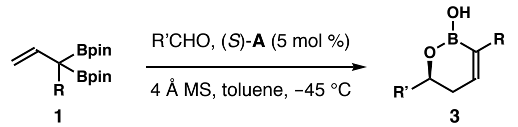

General procedure for CPA-catalyzed aldehyde addition with allylboronate 1: To a reaction vial containing a magnetic stir bar and freshly activated 4 Å MS (50 mg) was added phosphoric acid (*S*)-**A** (4 mg, 0.005 mmol). Toluene (0.2 mL) was added to the vial followed by dropwise addition of freshly distilled aldehyde (0.1 mmol, if it is a liquid). The mixture was placed in a −45 °C cold bath and stirred for 15 min. Allylboronate **1** (0.13 mmol, 1.3 equiv) in toluene (0.1 mL) was added slowly to the reaction mixture via a microliter syringe. The mixture was kept stirring at −45 °C and the reaction progress was monitored by ^{1}H NMR analysis. After complete consumption of the aldehyde, the reaction was filtered through a pad of silica gel. The filtrate was concentrated under reduced pressure. Purification of the crude product was performed by flash column chromatography (gradient elution with hexane and ethyl acetate) to give product **3**. The enantiopurities of **3** were determined by HPLC analysis of the phenylation products derived from Suzuki coupling with PhI.

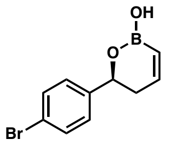

(*S*)-6-(4-bromophenyl)-5,6-dihydro-2H-1,2-oxaborinin-2-ol (**3d**) Prepared according to the general procedure. The crude mixture was purified by flash column chromatography (gradient elution with hexane and ethyl acetate, 15:1 to 5:1) to give compound **3d** in 95% yield (24 mg, *Z*:*E* > 20:1) as a white solid. Enantiomeric excess was determined by HPLC analysis of the corresponding phenylation product to be 98% ee (254 nm, 25 °C); t_1 = 6.48 min, t_2 = 6.84 min [(Chiralpak IB) hexane/*i*-PrOH, 90:10, 1.0 mL/min]. $[\alpha]_D^{20}$ = − 18.0 (c 0.5, $CHCl_3$).

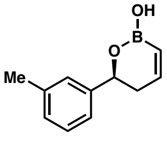

(*S*)-6-(m-tolyl)-5,6-dihydro-2H-1,2-oxaborinin-2-ol (3f) Prepared according to the general procedure. The crude mixture was purified by flash column chromatography (gradient elution with hexane and ethyl acetate, 15:1 to 3:1) to give compound **3f** in 96% yield (18 mg, *Z*:*E* > 20:1) as colorless oil. Enantiomeric excess was determined by HPLC analysis of the corresponding phenylation product to be > 99% ee (254 nm, 25 °C); t_1 = 10.1 min, t_2 = 11.3 min [(Chiralpak IG) hexane/*i*-PrOH, 95:5, 1.0 mL/min]. $[\alpha]_D^{20}$ = – 21.6 (c 0.3, $CHCl_3$).

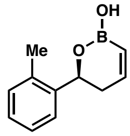

(*S*)-6-(o-tolyl)-5,6-dihydro-2H-1,2-oxaborinin-2-ol (3g) Prepared according to the general procedure. The crude mixture was purified by flash column chromatography (gradient elution with hexane and ethyl acetate, 15:1 to 3:1) to give compound **3g** in 96% yield (18 mg, *Z*:*E* > 20:1) as colorless oil. Enantiomeric excess was determined by HPLC analysis of the corresponding phenylation product to be 98% ee (254 nm, 25 °C); t_1 = 9.00 min, t_2 = 9.74 min [(Chiralpak IA) hexane/*i*-PrOH, 95:5, 1.0 mL/min]. $[\alpha]_D^{20}$ = – 20.4 (c 0.35, $CHCl_3$)

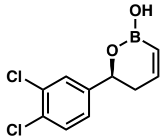

(*S*)-6-(3,4-dichlorophenyl)-5,6-dihydro-2*H*-1,2-oxaborinin-2-ol (3h) Prepared according to the general procedure. The crude reaction mixture was purified by flash column chromatography (gradient elution with hexane and ethyl acetate, 15:1 to 5:1) to give compound **3h** in 91% yield (22 mg, *Z*:*E* > 20:1) as white solid. Enantiomeric excess was determined by HPLC analysis of the corresponding phenylation product to be > 99% ee (254 nm, 25 °C); t_1 = 6.42 min, t_2 = 6.79 min [(Chiralpak IB) hexane/i-PrOH, 90:10, 1.0 mL/min]. $[\alpha]_D^{20}$ = – 15.2 (c 0.075, $CHCl_3$).

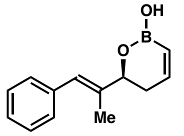

(*S*,*E*)-6-(1-phenylprop-1-en-2-yl)-5,6-dihydro-2H-1,2-oxaborinin-2-ol (3i) Prepared according to the general procedure. The crude mixture was purified by flash column chromatography (gradient elution with hexane and ethyl acetate, 15:1 to 3:1) to give compound **3i** in 89% yield (19 mg, *Z*:*E* > 20:1) as yellow oil. Enantiomeric excess was determined by HPLC analysis of the corresponding phenylation product to be 90% ee (254 nm, 25 °C); t_1 = 10.6 min, t_2 = 11.4 min [(Chiralpak IA) hexane/i-PrOH, 95:5, 1.0 mL/min]. $[\alpha]_D^{20}$ = – 8.8 (c 0.3, $CHCl_3$).

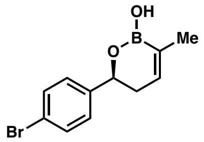

(*S*)-6-(4-bromophenyl)-3-methyl-5,6-dihydro-2H-1,2-oxaborinin-2-ol (3m) Prepared according to the general procedure. The crude mixture was purified by flash column chromatography (gradient elution with

hexane and ethyl acetate, 15:1 to 5:1) to give compound **3m** in 82% yield (22 mg, *E*:*Z* > 20:1) as white solid. Enantiomeric excess was determined by HPLC analysis of the corresponding phenylation product to be > 99% ee (254 nm, 25 °C); t_1 = 9.83 min, t_2 = 10.3 min [(Chiralpak IA) hexane/i-PrOH, 95:5, 1.0 mL/min]. ^{1}H NMR (500 MHz, $CDCl_3$) δ 7.48 (d, *J* = 8.4 Hz, 2H), 7.25 (d, *J* = 7.8 Hz, 2H), 6.52 (s, 1H), 5.05 (dd, *J* = 10.9, 5.2 Hz, 1H), 4.13 (s, 1H), 2.33 – 2.44 (m, 2H), 1.81 – 1.83 (m, 3H). ^{13}C NMR (126 MHz, $CDCl_3$) δ 143.0, 142.3, 131.8, 127.7, 121.6, 77.6, 36.9, 18.9. HRMS (ESI$^+$): m/z for $C_{11}H_{13}BBrO_2$ $[M+H]^+$ calcd. 267.0191, found: 267.0195. $[\alpha]_D^{20}$ = – 19.6 (c 0.2, $CHCl_3$).

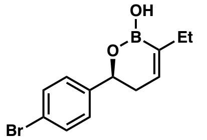

(*S*)-6-(4-bromophenyl)-3-ethyl-5,6-dihydro-2H-1,2-oxaborinin-2-ol (**3n**) Prepared according to the general procedure. The crude mixture was purified by flash column chromatography (gradient elution with hexane and ethyl acetate, 15:1 to 5:1) to give compound **3n** in 96% yield (27 mg, *E*:*Z* > 20:1) as white solid. Enantiomeric excess was determined by HPLC analysis of the corresponding phenylation product to be > 99% ee (254 nm, 25 °C); t_1 = 8.77 min, t_2 = 9.57 min [(Chiralpak IA) hexane/i-PrOH, 95:5, 1.0 mL/min]. ^{1}H NMR (600 MHz, $CDCl_3$) δ 7.48 (d, *J* = 8.5 Hz, 2H), 7.25 – 7.26 (d, *J* = 8.5 Hz, 2H), 6.50 (d, *J* = 3.7 Hz, 1H), 5.05 (dd, *J* = 11.6, 4.5 Hz, 1H), 4.15 (s, 1H), 2.41 – 2.45 (m, 1H), 2.34 – 2.40 (m, 1H), 2.14 – 2.24 (m, 3H), 1.06 (t, *J* = 7.4, 3H). ^{13}C NMR (151 MHz, $CDCl_3$) δ 142.3, 141.1, 131.8, 127.8, 121.6, 75.5, 36.8, 26.3, 14.1. HRMS (ESI$^+$): m/z for $C_{12}H_{15}BBrO_2$ $[M+H]^+$ calcd. 281.0348, found: 281.0351. $[\alpha]_D^{20}$ = – 21.6 (c 0.35, $CHCl_3$).

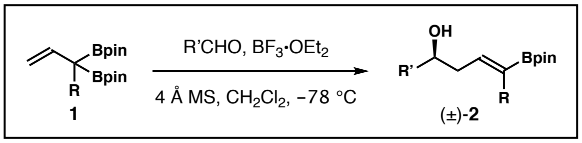

General procedure for the syntheses of (±)-2: To a reaction flask containing a stir bar were added allylboronate **1** (0.13 mmol, 1.3 equiv), freshly activated 4 Å MS (50 mg), and dichloromethane (2 mL). The mixture was cooled in a –78 °C cold bath and stirred for 5 min. Then $BF_3{\cdot}OEt_2$ (0.5 M in CH_2Cl_2, 40 μL, 0.02 mmol) was added. After stirring at –78 °C for 20 min, freshly distilled aldehyde (0.1 mmol, if it is a liquid) was added slowly to the reaction mixture via a microliter syringe. The reaction mixture was kept stirring at –78 °C until complete consumption of the aldehyde (typically after 12 h). Then a saturated aqueous solution of sodium bicarbonate (1 mL) and Et_2O (5 mL) were added to the flask. The reaction mixture was allowed to warm to ambient temperature. Brine (5 mL) was added, and the resulting mixture was stirred for another 3 h. Then the organic

layer was separated, and the aqueous layer was extracted with Et_2O (5 mL x 3). The combined organic extracts were dried over anhydrous sodium sulfate, filtered, and concentrated under reduced pressure. Purification of the crude product by flash column chromatography (gradient elution with hexane and ethyl acetate) provided product **2**.

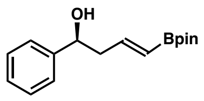

***rac*-(*E*)-1-phenyl-4-(4,4,5,5-tetramethyl-1,3,2-dioxaborolan-2-yl)-but-3-en-1-ol (2a)** Prepared according to the general procedure. The crude mixture was purified by flash column chromatography (gradient elution with hexane and ethyl acetate, 15:1 to 3:1) to give **2a** in 91% yield (25 mg, *E*:*Z* > 20:1) as colorless oil. ^{1}H NMR (600 MHz, $CDCl_3$) δ 7.33 – 7.38 (m, 4H), 7.26 – 7.29 (m, 1H), 6.65 (ddd, *J* = 17.9, 7.2, 6.5 Hz, 1H), 5.60 (ddd, *J* = 17.9, 2.8, 1.3 Hz, 1H), 4.78 – 4.82 (m, 1H), 2.56 – 2.65 (m, 2H), 1.98 (d, *J* = 3.2 Hz, 1H), 1.27 (s, 12H). ^{13}C NMR (151 MHz, $CDCl_3$) δ 150.0, 144.2, 128.8, 128.0, 126.1, 83.6, 73.4, 46.3, 25.13, 25.11. The data was consistent with data reported in the literature.[4]

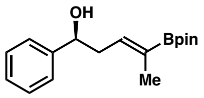

***rac*-(*Z*)-1-phenyl-4-(4,4,5,5-tetramethyl-1,3,2-dioxaborolan-2-yl) pent-3-en-1-ol** (**2b**) Prepared according to the general procedure. The crude mixture was purified by flash column chromatography (gradient elution with hexane and ethyl acetate, 15:1 to 3:1) to give compound **2b** in 80% yield (23 mg, *Z*:*E* = 11:1) as colorless oil. ^{1}H NMR (600 MHz, $CDCl_3$) δ 7.34 – 7.39 (m, 4H), 7.26 – 7.29 (m, 1H), 6.41 – 6.43 (m, 1H), 4.79 – 4.81 (m, 1H), 2.63 – 2.68 (m, 1H), 2.50 – 2.54 (m, 1H), 1.97 (d, *J* = 3.1 Hz, 1H), 1.69 (d, *J* = 0.8 Hz, 3H), 1.26 (s, 12H). ^{13}C NMR (151 MHz, CD_3CN) δ 144.6, 141.5, 128.8, 127.9, 1261, 83.6, 74.0, 39.3, 25.1, 14.6. HRMS (ESI^+): m/z for $C_{17}H_{26}BO_3$ $[M+H]^+$ calcd. 289.1975, found: 289.1979.

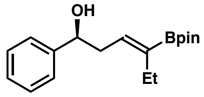

***rac*-(*Z*)-1-phenyl-4-(4,4,5,5-tetramethyl-1,3,2-dioxaborolan-2-yl)-hex-3-en-1-ol** (**2c**) Prepared according to the general procedure. The crude mixture was purified by flash column chromatography (gradient elution with hexane and ethyl acetate, 15:1 to 3:1) to give compound **2c** in 86% yield (26 mg, *Z*:*E* = 12:1) as colorless oil. ^{1}H NMR (600 MHz, $CDCl_3$) δ 7.34 – 7.39 (m, 4H), 7.27 – 7.30 (m, 1H), 6.34 – 6.37 (m, 1H), 4.77 – 4.79 (m, 1H), 2.66 (ddd, *J* = 14.8, 8.9, 7.9 Hz, 1H), 2.53 (ddd, *J* = 14.9, 6.5, 4.4 Hz, 1H), 2.12 – 2.18 (m, 2H), 1.97 (d, *J* = 3.1 Hz, 1H), 1.26 (s, 12H), 0.91 (t, *J* = 7.5 Hz, 3H). ^{13}C NMR (151 MHz, $CDCl_3$) δ 144.5, 140.3, 128.8, 127.9, 126.1, 83.5, 74.1, 38.9, 25.11, 25.08, 22.3, 14.9. HRMS (ESI^+): m/z for $C_{18}H_{28}BO_3$ $[M+H]^+$ calcd. 303.2131, found: 303.2129.

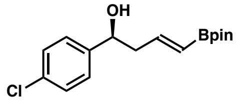

***rac*-(*E*)-1-(4-chlorophenyl)-4-(4,4,5,5-tetramethyl-1,3,2-dioxaborolan-2-yl)but-3-en-1-ol (2d)** Prepared according to the general procedure. The crude mixture was purified by flash column chromatography (gradient elution with hexane and ethyl acetate, 15:1 to 3:1) to give compound **2d** in 84% yield (26 mg, *E*:*Z* > 20:1) as colorless oil. ^{1}H NMR (600 MHz, $CDCl_3$) δ 7.29 – 7.32 (m, 4H), 6.60 (ddd, *J* = 18.0, 7.4, 6.2 Hz, 1H), 5.58 (d, *J* = 18.0 Hz, 1H), 4.78 (ddd, *J* = 7.9, 4.6, 2.8 Hz, 1H), 2.52 – 2.61 (m, 2H), 2.01 (d, *J* = 3.1 Hz, 1H), 1.26 (s, 12H). ^{13}C NMR (151 MHz, $CDCl_3$) δ 149.3, 142.6, 133.5, 128.9, 127.5, 83.6, 72.6, 46.3, 25.13, 25.11. [4]

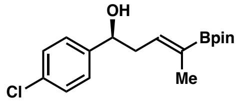

***rac*-(*Z*)-1-(4-chlorophenyl)-4-(4,4,5,5-tetramethyl-1,3,2-dioxaborolan-2-yl)pent-3-en-1-ol** (**2e**) Prepared according to the general procedure. The crude reaction mixture was purified by flash column chromatography (gradient elution with hexane and ethyl acetate, 15:1 to 3:1) to give compound **2e** in 81% yield (26 mg, *Z*:*E* = 15:1) as colorless oil. ^{1}H NMR (600 MHz, $CDCl_3$) δ 7.31 (m, 4H), 6.36 (ddd, *J* = 8.0, 6.3, 1.8 Hz, 1H), 4.76 – 4.79 (m, 1H), 2.59 – 2.64 (m, 1H), 2.47 – 2.51 (m, 1H), 1.98 (d, *J* = 3.1 Hz, 1H), 1.68 (s, 3H), 1.26 (s, 12H). ^{13}C NMR (151 MHz, $CDCl_3$) δ 142.9, 140.8, 133.5, 128.9, 127.5, 83.7, 73.3, 39.2, 25.1, 14.6. HRMS (ESI$^+$): m/z for $C_{17}H_{25}BClO_3$ $[M+H]^+$ calcd. 323.1585, found: 323.1582.

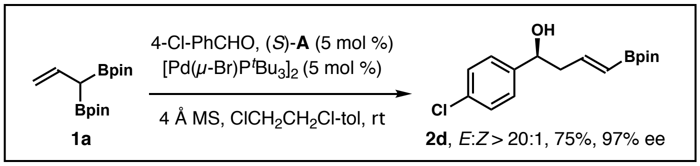

Enantioselective synthesis of alcohol 2d: In an Ar-filled glove box, $[Pd(\mu\text{-}Br)(P^tBu_3)]_2$ (4 mg, 0.005 mmol, 5 mol %), acid (*S*)-**A** (4 mg, 0.005 mol, 5 mol %), molecular sieves 4 Å (50 mg), boronate **1a** (0.13 mmol, 1.3 equiv), toluene (0.2 mL) and a stir bar were added sequentially to an oven-dried vial. The vial was sealed with a rubber septum and removed from the glove box. After stirring at ambient temperature for 10 min, *para*-Cl-PhCHO (14 mg, 0.1 mmol, 1.0 equiv) in $ClCH_2CH_2Cl$ (0.2 mL) was added. The reaction mixture was kept stirring at ambient temperature. Upon completion, saturated aqueous solution of $NaHCO_3$ (1 mL) was added. The organic layer was separated, and the aqueous layer was extracted with Et_2O (1 mL x 3). The combined organic layers were washed with brine, dried over anhydrous sodium sulfate, filtered, and the filtrate was concentrated under reduced pressure. Purification of the crude reaction product was performed by flash column chromatography (gradient elution with hexane and ethyl

acetate, 15:1 to 3:1) to give compound **2d** in 75% yield (23 mg, *E*:*Z* > 20:1) as colorless oil. Enantiomeric excess was determined by HPLC analysis to be 97% ee (254 nm, 25 °C); t_1 = 8.48 min, t_2 = 9.77 min [(Chiralpak IA) hexane/i-PrOH, 95:5, 1.0 mL/min]. $[\alpha]_D^{20}$ = − 8.0 (c 0.2, $CHCl_3$).

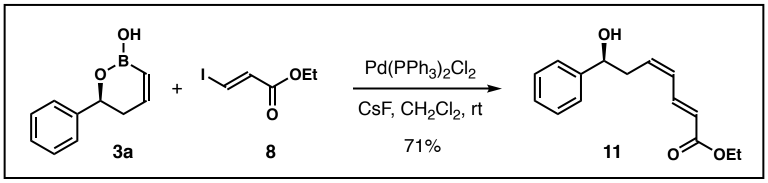

Ethyl (*S*,2*E*,4*Z*)-7-hydroxy-7-phenylhepta-2,4-dienoate (11): In an Ar-filled glove box, $Pd(PPh_3)_2Cl_2$ (0.01 mmol, 10 mol %), CsF (0.2 mmol, 2.0 equiv) and a stir bar were sequentially added into an oven-dried vial. The vial was sealed with a rubber septum and removed from the glove box. A solution of **3a** (17 mg, 0.1 mmol, 1.0 equiv) and *E*-vinyl iodide **8** (29 mg, 0.13 mmol, 1.3 equiv) in CH_2Cl_2 (1 mL) were added to the vial, and the mixture was allowed to stir at ambient temperature. After completion consumption of **3a**, Et_2O (2 mL) was added, and the resulting mixture was filtered through a short pad of silica gel. The filtrate was concentrated under reduced pressure. Purification of the crude reaction product was performed by flash column chromatography (gradient elution with hexane and ethyl acetate, 10:1 to 2:1) to give product **11** in 71% yield (17 mg) as colorless oil. ^1H NMR (600 MHz, $CDCl_3$) δ 7.54 (ddd, J = 15.3, 11.7, 1.2 Hz, 1H), 7.35 – 7.38 (m, 4H), 7.28 – 7.31 (m, 1H), 6.22 – 6.26 (m, 1H), 5.86 – 5.90 (m, 2H), 4.78 – 4.81 (m, 1H), 4.20 (q, J = 7.2 Hz, 2H), 2.80 – 2.86 (m, 1H), 2.70 – 2.76 (m, 1H), 1.97 (d, J = 3.3 Hz, 1H), 1.30 (t, J = 7.1 Hz, 3H). ^{13}C NMR (151 MHz, $CDCl_3$) δ 167.4, 143.8, 139.2, 136.3, 129.3, 128.9, 128.2, 126.1, 122.7, 74.1, 60.7, 38.1, 14.7. [5]

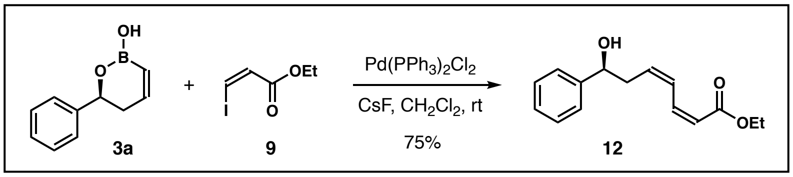

Ethyl (*S*,2*Z*,4*Z*)-7-hydroxy-7-phenylhepta-2,4-dienoate (12): In an Ar-filled glove box, $Pd(PPh_3)_2Cl_2$ (0.01 mmol, 10 mol %), CsF (0.2 mmol, 2.0 equiv) and a stir bar were sequentially added into an oven-dried vial. The vial was sealed with a rubber septum and removed from the glove box. A solution of **3a** (17 mg, 0.1 mmol, 1.0 equiv) and *Z*-vinyl iodide **9** (29 mg, 0.13 mmol, 1.3 equiv) in CH_2Cl_2 (1 mL) were added to the vial, and the

mixture was allowed to stir at ambient temperature. After completion consumption of **3a**, Et_2O (2 mL) was added, and the resulting mixture was filtered through a short pad of silica gel. The filtrate was concentrated under reduced pressure. Purification of the crude reaction product was performed by flash column chromatography (gradient elution with hexane and ethyl acetate, 10:1 to 2:1) to give product **12** in 75% yield (18 mg) as colorless oil. ^{1}H NMR (600 MHz, CDCl3) δ 7.41 – 7.43 (m, 1H), 7.34 – 7.40 (m, 4H), 7.28 – 7.31 (m, 1H), 6.87 (ddd, J = 11.7, 11.7, 1.2 Hz, 1H), 5.98 – 5.86 (m, 1H), 5.69 (d, J = 11.5 Hz, 1H), 4.78 (ddd, J = 8.1, 5.2, 3.3 Hz, 1H), 4.18 (q, J = 7.1 Hz, 2H), 2.76 – 2.82 (m, 1H), 2.73 – 2.54 (m, 1H), 1.99 (d, J = 3.3 Hz, 1H), 1.29 (t, J = 7.1 Hz, 3H). ^{13}C NMR (151 MHz, $CDCl_3$) δ 166.7, 143.9, 138.6, 136.2, 128.9, 128.2, 127.4, 126.1, 118.9, 74.0, 60.4, 37.6, 14.6. HRMS (ESI$^+$): m/z for $C_{15}H_{19}O_3$ $[M+H]^+$ calcd. 247.1334, found: 247.1338.

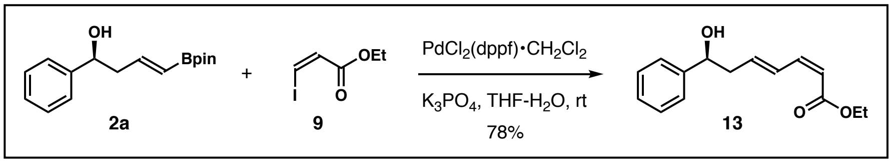

Ethyl (*S*,2*Z*,4*E*)-7-hydroxy-7-phenylhepta-2,4-dienoate (13): In an Ar-filled glove box, vinylboronate **2a** (27 mg, 0.1 mmol, 1.0 equiv), *Z*-vinyl iodide **9** (29 mg, 0.13 mmol, 1.3 equiv), $PdCl_2(dppf)•CH_2Cl_2$ (8 mg, 0.01 mmol, 10 mol %), K_3PO_4 (28 mg, 0.13 mmol, 1.3 equiv), THF (1 mL) and a stir bar were sequentially added into a reaction vial. The vial was sealed with a rubber septum and removed from the glove box. Then water (0.1 mL) was added to the vial under an argon atmosphere. The vial was then sealed with a cap containing a PTFE-lined silicone septum and stirred at ambient temperature for 12 h. After completion of the reaction, the mixture was filtered through a short pad of Celite. Brine (1 mL) was added, and the mixture was extracted with Et_2O (1 mL x 3). The combined organic layers were dried over anhydrous Na_2SO_4, filtered, and concentrated under reduced pressure. Purification of the crude reaction product was performed by flash column chromatography (gradient elution with hexane and ethyl acetate, 10:1 to 2:1) to give product **13** in 78% yield (19 mg) as colorless oil. ^{1}H NMR (500 MHz, $CDCl_3$) δ 7.49 (ddd, *J* = 15.3, 11.3, 2.5 Hz, 1H), 7.35 – 7.37 (m, 4H), 7.28 – 7.32 (m, 1H), 6.55 (ddd, *J* = 11.4, 11.3, 0.8 Hz, 1H), 6.0 (ddd, *J* = 15.3, 15.3, 7.2 Hz, 1H), 5.61 (d, *J* = 11.4 Hz, 1H), 4.79 – 4.82 (m, 1H), 4.18 (q, *J* = 7.1 Hz, 2H), 2.61 – 2.72 (m, 2H), 1.97 (dd, *J* = 3.3, 1.0 Hz, 1H), 1.29 (t, *J* = 7.1 Hz, 3H). ^{13}C NMR (126 MHz, $CDCl_3$) δ 166.8, 144.9, 144.0, 140.5, 129.9, 128.9, 128.1, 126.1, 117.1, 74.0, 60.3, 43.1, 14.6. [4]

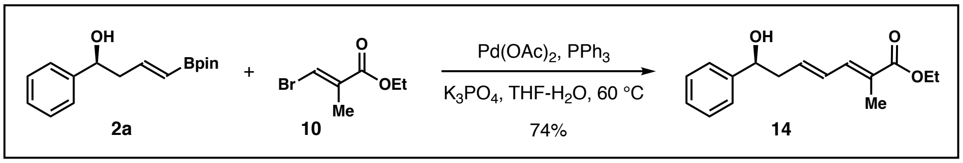

Ethyl (*S*,2*E*,4*E*)-7-hydroxy-2-methyl-7-phenylhepta-2,4-dienoate (14): In an Ar-filled glove box, $Pd(OAc)_2$ (0.01 mmol, 10 mol %), PPh_3 (0.02 mmol, 20 mol %) K_3PO_4 (0.2 mmol, 2.0 equiv) and a stir bar were sequentially added into an oven-dried vial. The vial was sealed with a rubber septum and removed from the glove box. A solution of vinylboronate **2a** (27 mg, 0.1 mmol, 1.0 equiv) and vinyl bromide **10** (25 mg, 0.13 mmol, 1.3 equiv) in THF (1 mL) were added to the vial. Then water (0.1 mL) was added under an argon atmosphere. The resulting mixture was kept stirring at 60 °C. After completion consumption of vinylboronate **2a**, Et_2O (2 mL) was added, and the mixture was filtered through a short pad of silica gel. The filtrate was concentrated under reduced pressure. Purification of the crude reaction product was performed by column chromatography (gradient elution with hexane and ethyl acetate, 10:1 to 2:1) to give product **14** in 74% yield (19 mg) as colorless oil. ^{1}H NMR (600 MHz, $CDCl_3$) δ 7.36 – 7.37 (m, 4H), 7.28 – 7.31 (m, 1H), 7.14 – 7.16 (m, 1H), 6.44 (dd, J = 15.1, 11.3 Hz, 1H), 6.02 – 6.07 (m, 1H), 4.64 – 4.67 (m, 1H), 4.20 (q, J = 7.1 Hz, 2H), 2.71 – 2.61 (m, 2H), 1.98 (d, J = 3.1 Hz, 1H), 1.92 (s, 3H), 1.30 (t, J = 7.1 Hz, 3H). ^{13}C NMR (151 MHz, $CDCl_3$) δ 168.9, 143.9, 138.1, 137.8, 129.4, 128.9, 128.1, 126.7, 126.1, 74.0, 60.9, 43.4, 14.7, 13.0. [6]

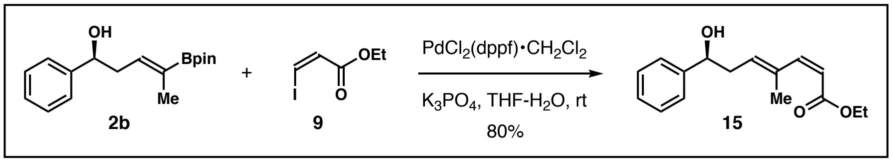

Ethyl (*S*,2*Z*,4*E*)-7-hydroxy-4-methyl-7-phenylhepta-2,4-dienoate (15): In an Ar-filled glove box, boronate **2b** (29 mg, 0.1 mmol, 1.0 equiv), vinyl iodide **9** (29 mg, 0.13 mmol, 1.3 equiv), $PdCl_2(dppf)•CH_2Cl_2$ (8 mg, 0.01 mmol, 10 mol %), K_3PO_4 (28 mg, 0.13 mmol, 1.3 equiv), THF (1 mL) and a stir bar were sequentially added into a reaction vial. The vial was sealed with a rubber septum and removed from the glove box. Then water (0.1 mL) was added to the vial under an argon atmosphere. The vial was then sealed with a cap containing a PTFE-lined silicone septum and stirred at ambient temperature. After completion of the reaction, the mixture was filtered through a short pad of Celite. Brine (1 mL) was added, and the mixture was extracted with Et_2O (1 mL x 3). The combined organic layers were dried, filtered, and concentrated under reduced pressure. Purification of the crude product was performed by flash column chromatography (gradient elution

with hexane and ethyl acetate, 10:1 to 2:1) to give product **15** in 80% yield (21 mg) as colorless oil. ^{1}H NMR (600 MHz, $CDCl_3$) δ 7.39 – 7.41 (m, 2H), 7.34 – 7.37 (m, 2H), 7.27 – 7.28 (m, 1H), 6.50 (d, J = 12.1 Hz, 1H), 5.70 (d, J = 12.2 Hz, 1H), 5.65 – 5.67 (m, 1H), 4.78 – 4.81 (m, 1H), 4.17 (q, J = 7.1 Hz, 2H), 2.92 (s, 1H), 2.52 – 2.62 (m, 2H), 1.83 (d, J = 1.5 Hz, 3H), 1.28 (t, J = 7.1 Hz, 3H). ^{13}C NMR (151 MHz, $CDCl_3$) δ 167.0, 147.5, 144.3, 135.5, 130.0, 128.7, 127.7, 126.0, 118.6, 73.7, 60.7, 39.0, 15.9, 14.6. HRMS (ESI^+): m/z for $C_{16}H_{21}O_3$ $[M+H]^+$ calcd. 261.1490, found: 261.1494.

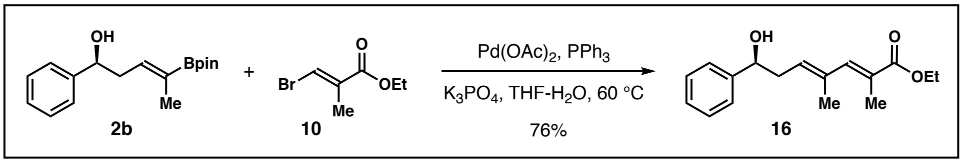

Ethyl (*S*,2*E*,4*E*)-7-hydroxy-2,4-dimethyl-7-phenylhepta-2,4-dienoate (16): In an Ar-filled glove box, $Pd(OAc)_2$ (0.01 mmol, 10 mol %), PPh_3 (0.02 mmol, 20 mol %) K_3PO_4 (0.2 mmol, 2.0 equiv) and a stir bar were sequentially added into an oven-dried vial. The vial was sealed with a rubber septum and removed from the glove box. A solution of vinylboronate **2b** (29 mg, 0.1 mmol, 1.0 equiv) and vinyl bromide **10** (25 mg, 0.13 mmol, 1.3 equiv) in THF (1 mL) were added to the vial. Then water (0.1 mL) was added to the vial under an argon atmosphere, and the mixture was stirred at 60 °C. After completion consumption of vinylboronate **2b**, Et_2O (2 mL) was added, and the mixture was filtered through a short pad of silica gel. The filtrate was concentrated under reduced pressure. Purification of the crude product was performed by column chromatography (gradient elution with hexane and ethyl acetate, 10:1 to 2:1) to give product **16** in 76% yield (21 mg) as colorless oil. ^{1}H NMR (600 MHz, $CDCl_3$) δ 7.35 – 7.38 (m, 4H), 7.28 – 7.31 (m, 1H), 7.09 (s, 1H), 5.61 – 5.64 (m, 1H), 4.78 – 4.80 (m, 1H), 4.19 (q, J = 7.1 Hz, 2H), 2.65 – 2.70 (m, 1H), 2.56 – 2.61 (m, 1H), 1.97 (s, 1H), 1.95 (s, 3H), 1.79 (s, 3H), 1.29 (t, J = 7.2 Hz, 4H). ^{13}C NMR (151 MHz, $CDCl_3$) δ 169.5, 144.1, 142.8, 135.3, 131.3, 128.8, 128.1, 126.1 (two overlapping carbon signals), 74.2, 61.0, 38.5, 16.9, 14.6, 14.4. HRMS (ESI^+): m/z for $C_{17}H_{23}O_3$ $[M+H]^+$ calcd. 275.1647, found: 275.1643.

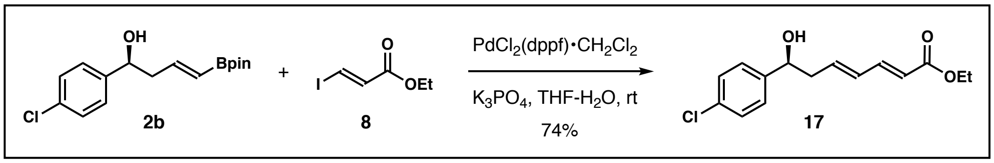

Ethyl (*S*,2*E*,4*E*)-7-(4-chlorophenyl)-7-hydroxyhepta-2,4-dienoate (17): In an Ar-filled glove box, vinylboronate **2d** (31 mg, 0.1 mmol, 1.0 equiv), vinyl iodide **8** (29 mg, 0.13

mmol, 1.3 equiv), $PdCl_2(dppf)•CH_2Cl_2$ (8 mg, 0.01 mmol, 10 mol %), K_3PO_4 (28 mg, 0.13 mmol, 1.3 equiv), THF (1 mL) and a stir bar were sequentially added into a reaction vial. The vial was sealed with a rubber septum and removed from the glove box. Then water (0.1 mL) was added to the mixture under an argon atmosphere. The vial was sealed with a cap containing a PTFE-lined silicone septum and kept stirring at ambient temperature. After completion of the reaction (~12 h), the mixture was filtered through a short pad of Celite. Brine (1 mL) was added, and the mixture was extracted with Et_2O (1 mL x 3). The combined organic layers were dried over anhydrous Na_2SO_4, filtered, and concentrated under reduced pressure. Purification of the crude reaction product was performed by flash column chromatography (gradient elution with hexane and ethyl acetate, 10:1 to 2:1) to give product **17** in 74% yield (21 mg) as white solid. ^{1}H NMR (600 MHz, $CDCl_3$) δ 7.33 (d, J = 8.5 Hz, 2H), 7.28 (d, J = 8.5 Hz, 2H), 7.23 (dd, J = 15.4, 10.9 Hz, 1H), 6.24 (dd, J = 15.2, 11.0 Hz, 1H), 6.04 – 6.09 (m, 1H), 5.82 (d, J = 15.4 Hz, 1H), 4.77 – 4.80 (m, 1H), 4.19 (q, J = 7.1 Hz, 2H), 2.58 – 2.60 (m, 2H), 1.96 (d, J = 3.3 Hz, 1H), 1.29 (t, J = 7.1 Hz, 3H). ^{13}C NMR (151 MHz, $CDCl_3$) δ 167.4, 144.4, 142.3, 138.8, 133.8, 131.9, 129.1, 127.5, 121.1, 73.2, 60.7, 43.1, 14.6. HRMS (ESI$^+$): m/z for $C_{15}H_{18}ClO_3$ $[M+H]^+$ calcd. 281.0944, found: 281.0949.

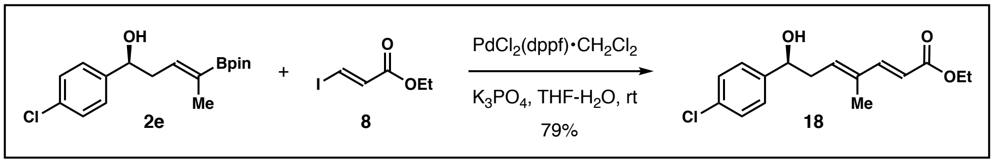

Ethyl (*S*,2*E*,4*E*)-7-(4-chlorophenyl)-7-hydroxy-2-methylhepta-2,4-dienoate (18): In an Ar-filled glove box, vinylboronate **2e** (32 mg, 0.1 mmol, 1.0 equiv), vinyl iodide **8** (29 mg, 0.13 mmol, 1.3 equiv), $PdCl_2(dppf)•CH_2Cl_2$ (8 mg, 0.01 mmol, 10 mol %), K_3PO_4 (28 mg, 0.13 mmol, 1.3 equiv), THF (1 mL) and a stir bar were sequentially added into a reaction vial. The vial was sealed with a rubber septum and removed from the glove box. Then water (0.1 mL) was added to the mixture under an argon atmosphere. The vial was sealed with a cap containing a PTFE-lined silicone septum and was kept stirring at ambient temperature. After completion of the reaction (~12 h), the mixture was filtered through a short pad of Celite. Brine (1 mL) was added, and the mixture was extracted with Et_2O (1 mL x 3). The combined organic layers were dried over anhydrous Na_2SO_4, filtered, and concentrated under reduced pressure. Purification of the crude reaction product was performed by flash column chromatography (gradient elution with hexane and ethyl acetate, 10:1 to 2:1) to give product **18** in 79% yield (23 mg) as white solid. ^{1}H NMR (600 MHz, $CDCl_3$) δ 7.27 – 7.37 (m, 5H), 5.87 – 5.89 (m, 1H), 5.81 (d, J = 15.7 Hz,

1H), 4.78 – 4.80 (m, 1H), 4.20 (q, *J* = 7.1 Hz, 2H), 2.66 – 2.72 (m, 1H), 2.58 – 2.63 (dt, *J* = 14.5, 6.5 Hz, 1H), 1.96 (d, *J* = 3.2 Hz, 1H), 1.72 (s, 3H), 1.30 (t, *J* = 7.1 Hz, 3H). ^{13}C NMR (151 MHz, $CDCl_3$) δ 167.8, 149.1, 142.5, 136.2, 135.9, 133.8, 129.0, 127.5, 117.0, 73.4, 60.6, 38.9, 14.7, 12.7. HRMS (ESI^+): m/z for $C_{16}H_{20}ClO_3$ $[M+H]^+$ calcd. 295.1101, found: 295.1100.

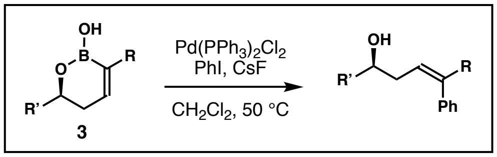

General procedure for the Suzuki Coupling with PhI for the determination of the enantiopurity: In an Ar-filled glove box, $Pd(PPh_3)_2Cl_2$ (2 mg, 0.0025 mmol, 5.0 mol %), CsF (15 mg, 0.1 mmol, 2.0 equiv) and a magnetic stirring bar were sequentially added to an oven-dried reaction tube. The tube was sealed with rubber septum and taken outside the glove box. In another reaction tube, boronate **3** (0.05 mmol, 1.0 equiv), iodobenzene (20 mg, 0.05 mmol, 1.0 equiv), and CH_2Cl_2 (1.0 mL) were sequentially added to the tube under an Ar atmosphere. The solution was transferred into the reaction tube charged with the catalyst and CsF via a syringe under an Ar atmosphere. The resulting reaction mixture was allowed to stir at 50 °C for 12 h. Upon completion of the reaction, the mixture was filtered through a pad of Celite. The filtrate was concentrated under reduced pressure and the crude mixture was purified by flash column chromatography to give the phenylation product, which was used for the HPLC analysis.

References:

1. (a) Hwang, C.; Lee, Y.; Kim, M.; Seo, Y.; Cho, S. H. *Angew. Chem., Int. Ed.* **2022**, e202209079. (b) Fang, T.; Xu, L.; Qin, Y.; Jiang, N.; Liu, C. *Chin. J. Org. Chem.* **2023**, *43*, 777-780.
2. Shin, M.; Kim, M.; Hwang, C.; Lee, H.; Kwon, H.; Park, J.; Lee, E.; Cho, S. H. *Org. Lett.* **2020**, *22*, 2476-2480.
3. McNulty, L.; Kohlbacher, K.; Borin, K.; Dodd, B.; Bishop, J.; Fuller, L.; Wright, Z. *J. Org. Chem.* **2020**, 75, 6001-6004.
4. Miura, T.; Oku, N.; Murakami, M. *Angew. Chem., Int. Ed.* **2019**, *58*, 14620-14624.
5. Woody, D.; Padarti, A.; Han, H. *Org. Lett.* **2018**, *20*, 2472-2476.
6. Eggert, A.; Etling, C.; Millbrodt, L.; Schulz, G.; Kalesse, M. *Org. Lett.* **2021**, *23*, 8722-8726.

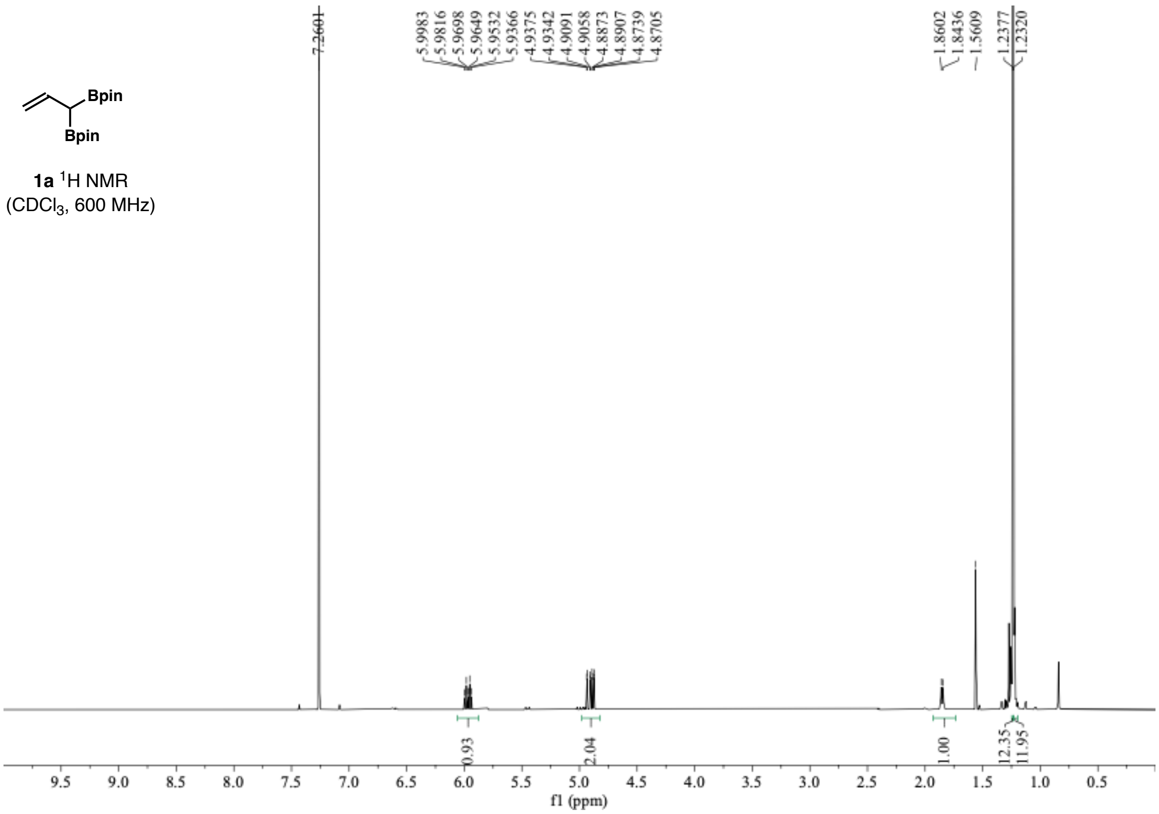
Bpin
Bpin
1a 1H NMR
(CDCl3, 600 MHz)
7.2601
5.9983
5.9816
5.9698
5.9649
5.9532
5.9366
4.9375
4.9342
4.9091
4.9058
4.8873
4.8907
4.8739
4.8705
1.8602
1.8436
1.5609
1.2377
1.2320
0.93
2.04
1.00
12.35
11.95
9.5 9.0 8.5 8.0 7.5 7.0 6.5 6.0 5.5 5.0 4.5 4.0 3.5 3.0 2.5 2.0 1.5 1.0 0.5
f1 (ppm)

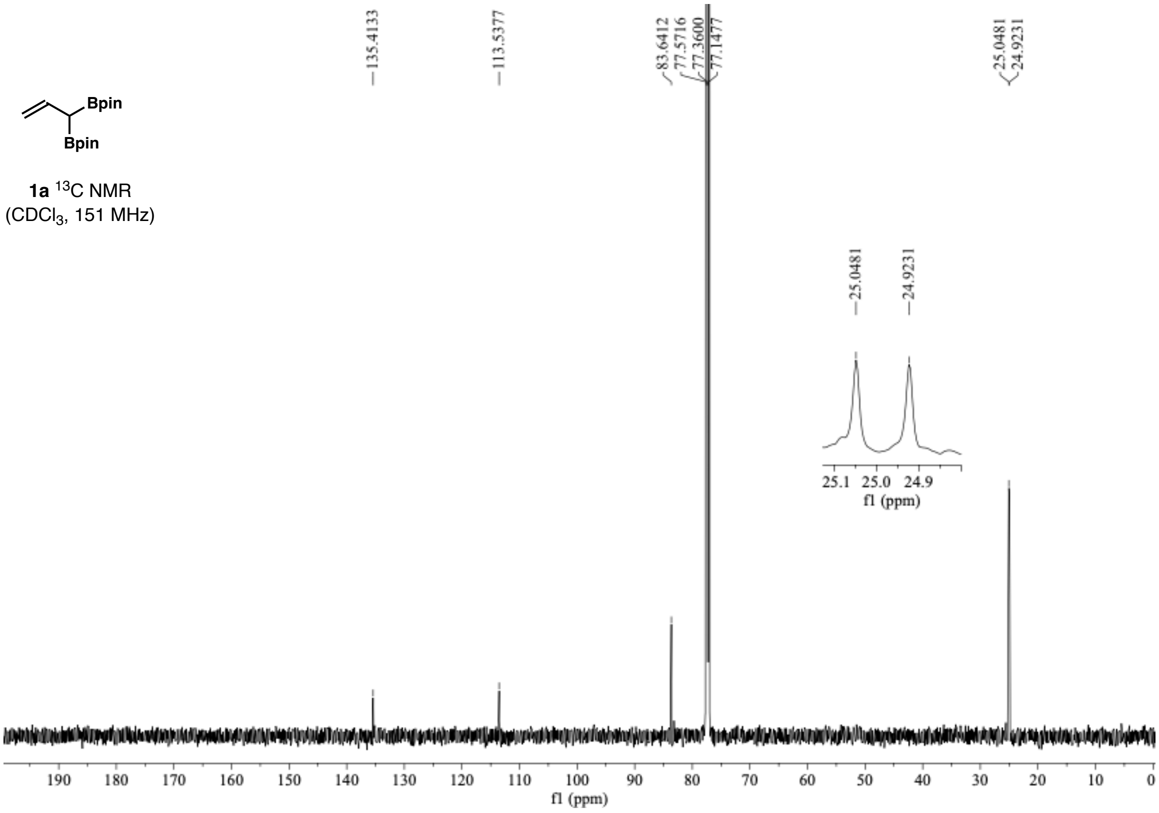
Bpin
Bpin
1a 13C NMR
(CDCl3, 151 MHz)
135.4133
113.5377
83.6412
77.5716
77.3600
77.1477
25.0481
24.9231
25.0481
24.9231
25.1 25.0 24.9
f1 (ppm)
190 180 170 160 150 140 130 120 110 100 90 80 70 60 50 40 30 20 10 0
f1 (ppm)

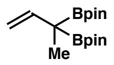

1b ^{1}H NMR
($CDCl_3$, 600 MHz)

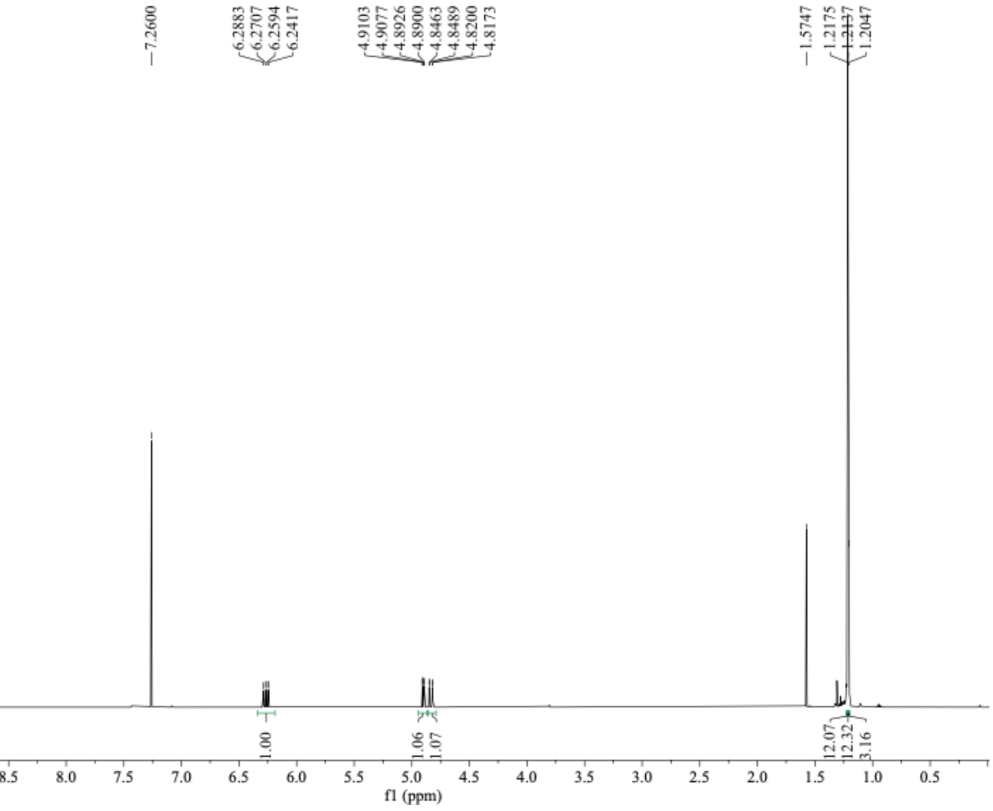

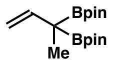

1b ^{13}C NMR
($CDCl_3$, 151 MHz)

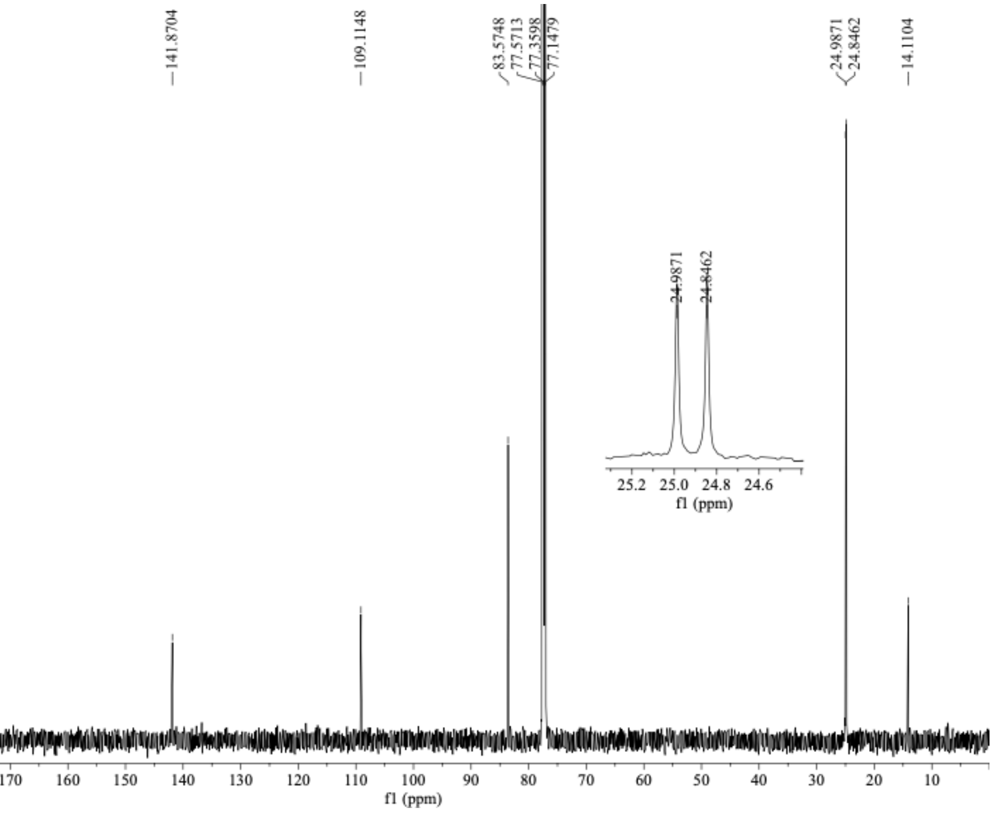

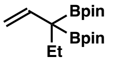

1c ^{1}H NMR
($CDCl_3$, 600 MHz)

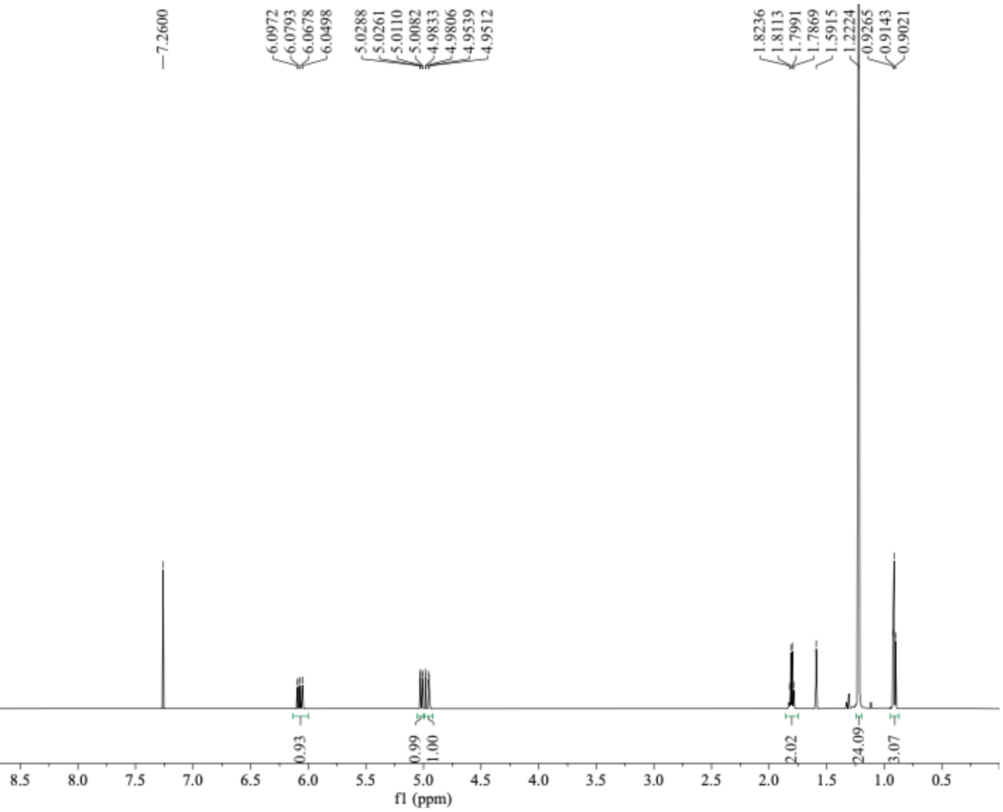

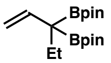

1c ^{13}C NMR
($CDCl_3$, 151 MHz)

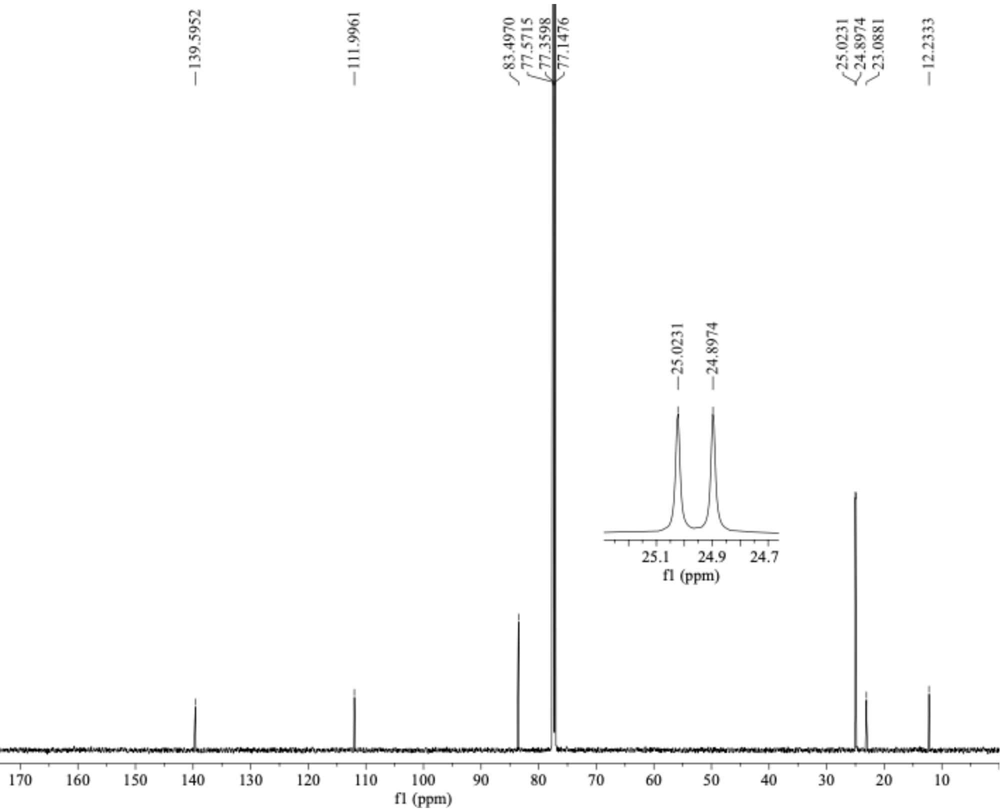

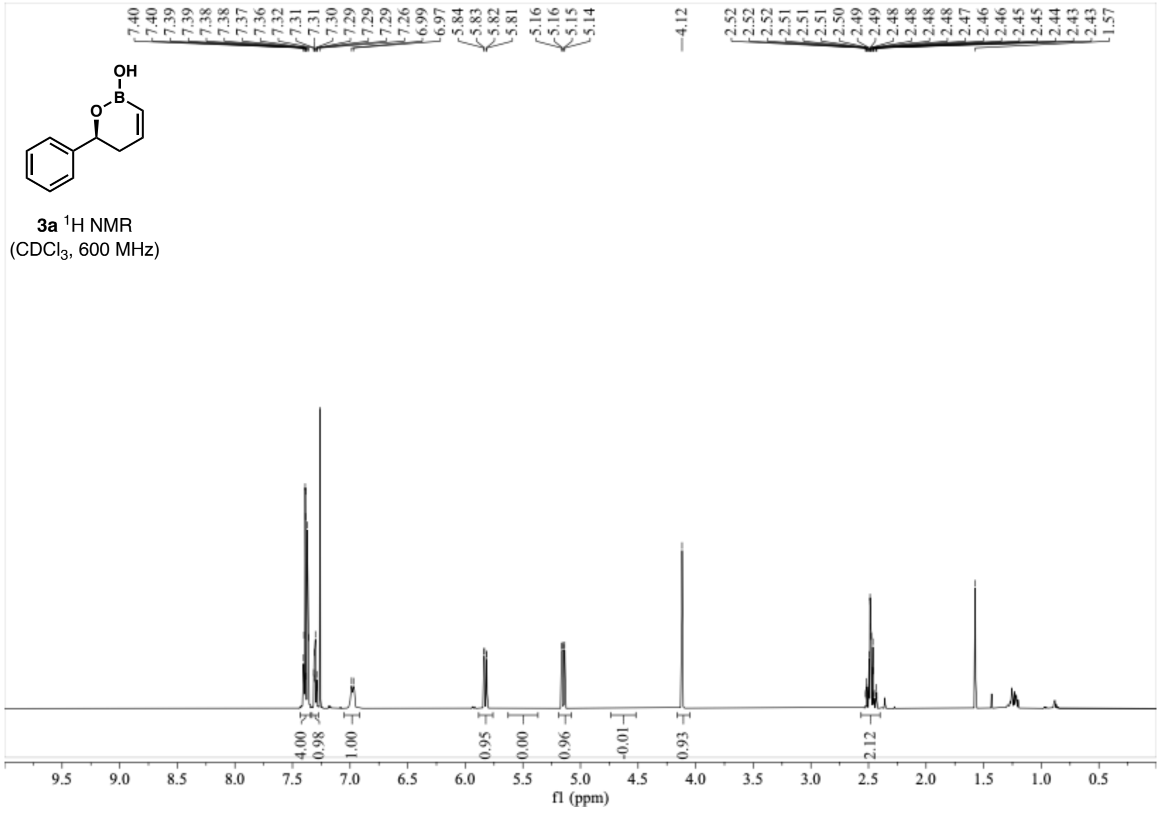
3a ^{1}H NMR
($CDCl_3$, 600 MHz)
f1 (ppm)

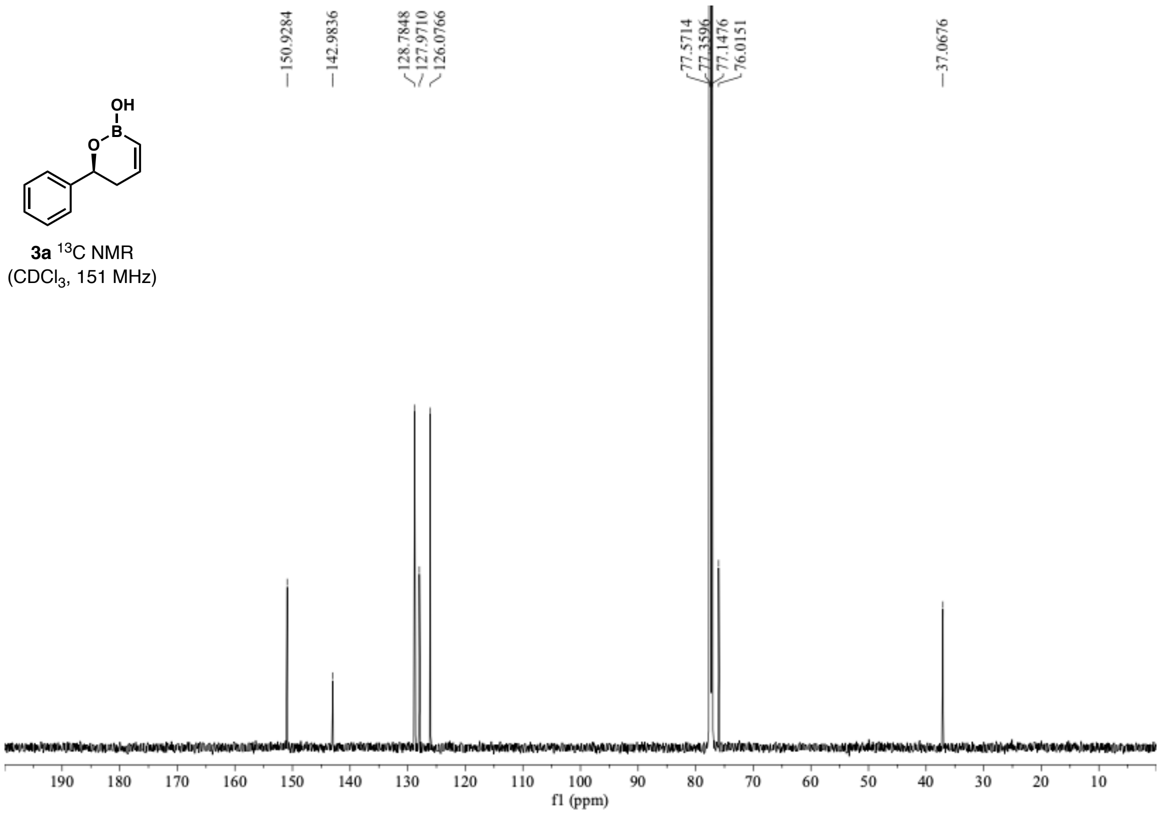
3a ^{13}C NMR
($CDCl_3$, 151 MHz)
f1 (ppm)

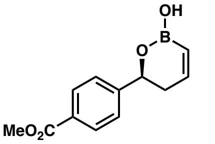

3b ^{1}H NMR
($CDCl_3$, 600 MHz)

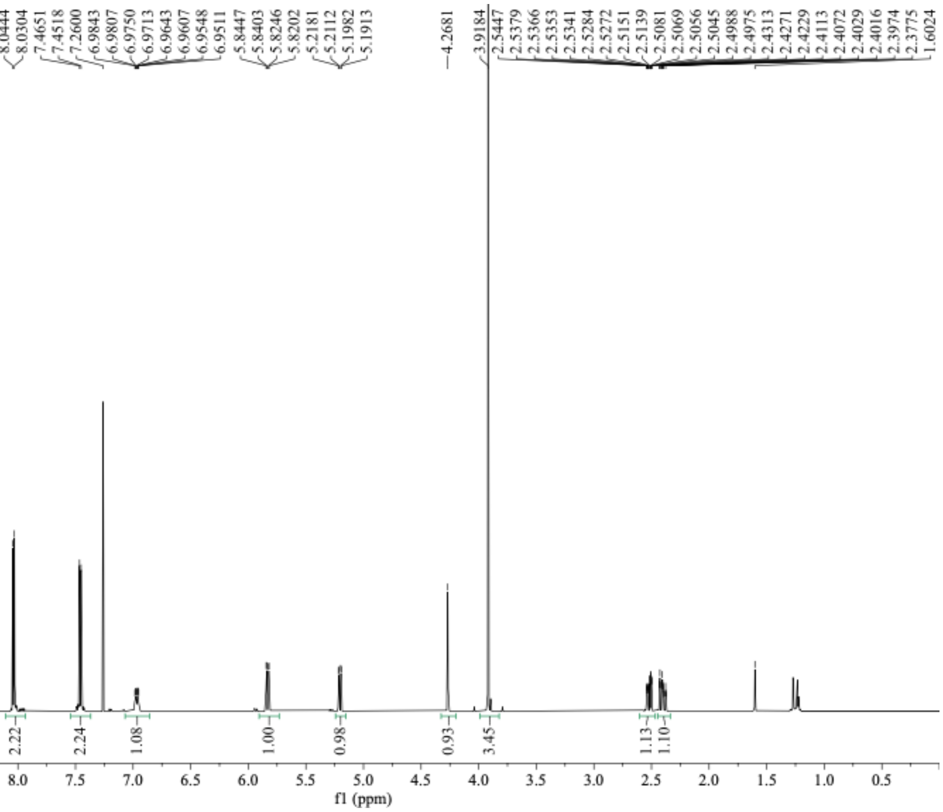

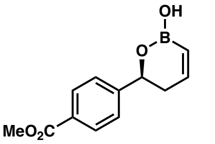

3b ^{13}C NMR
($CDCl_3$, 151 MHz)

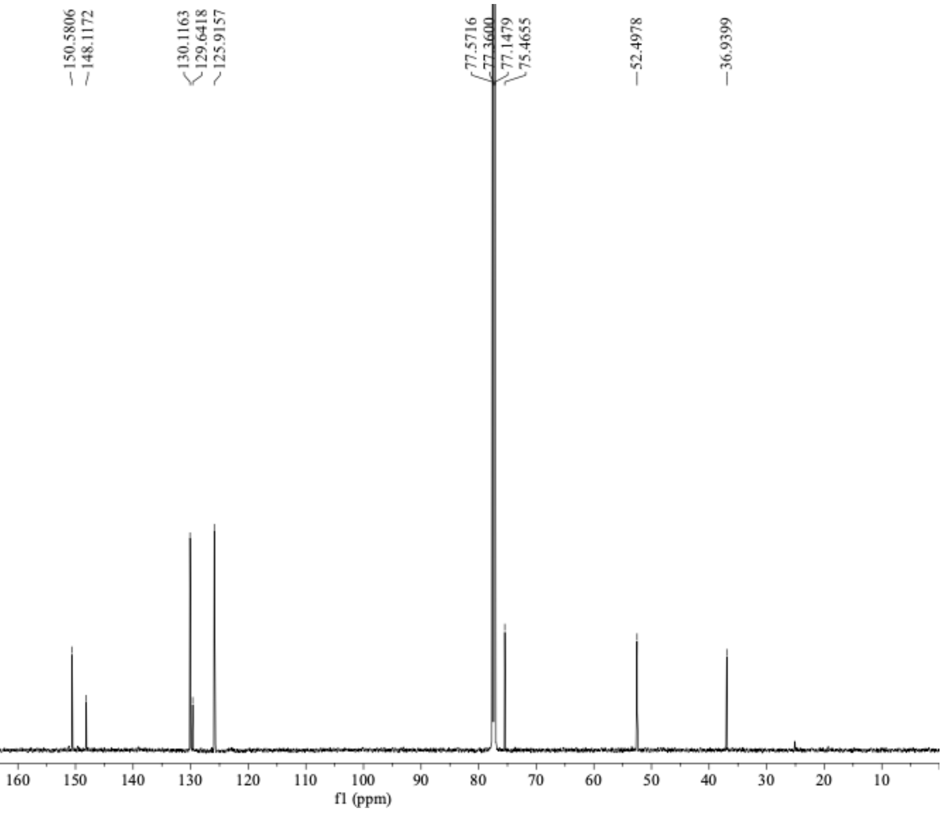

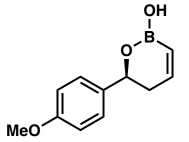

3c ^{1}H NMR
($CDCl_3$, 600 MHz)

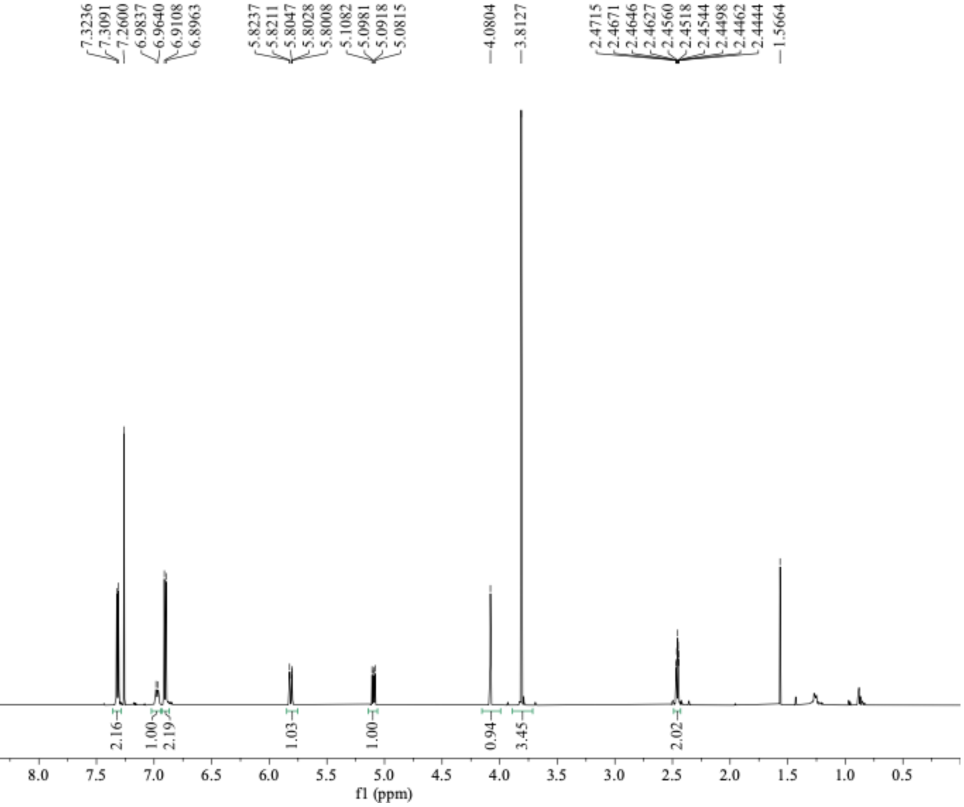

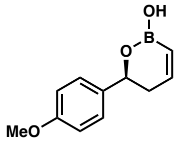

3c ^{13}C NMR
($CDCl_3$, 151 MHz)

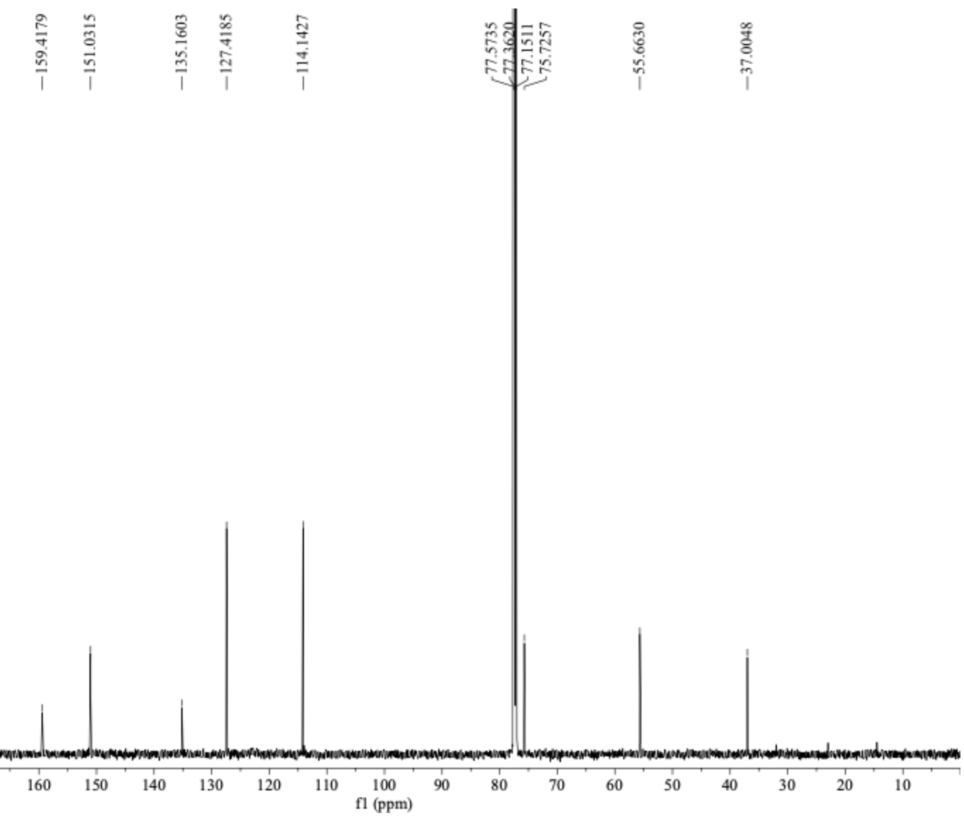

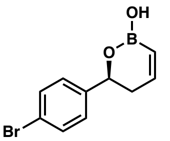

3d ^{1}H NMR
($CDCl_3$, 400 MHz)

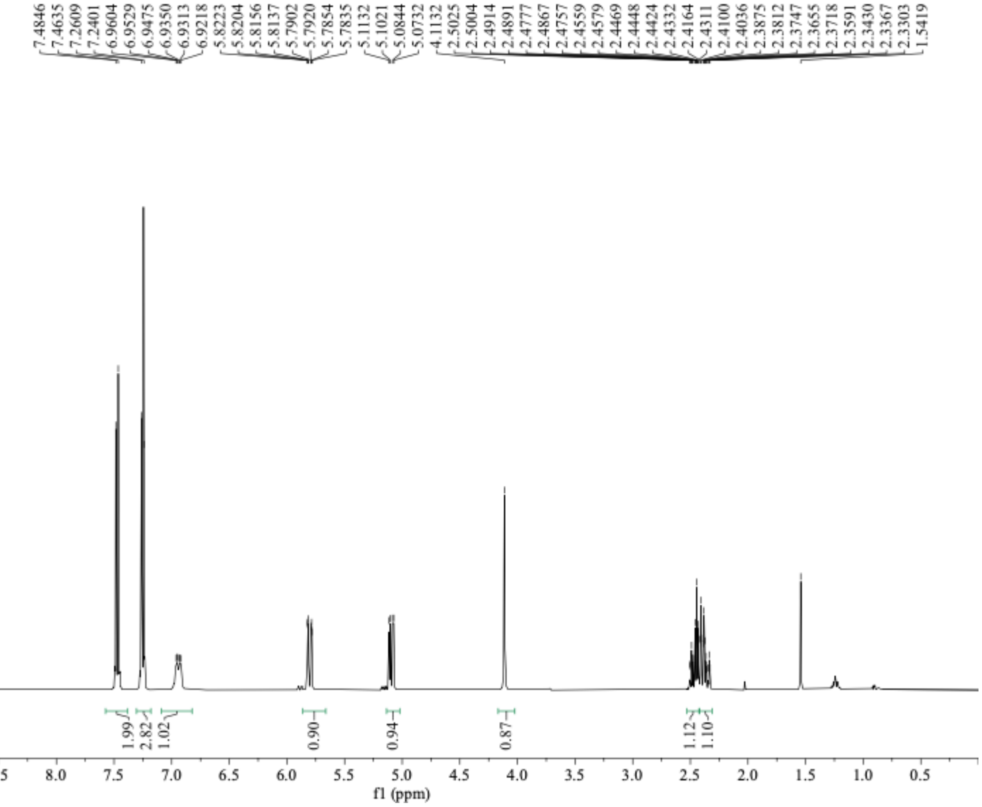

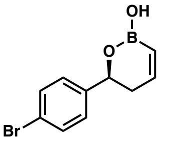

3d ^{13}C NMR
($CDCl_3$, 151 MHz)

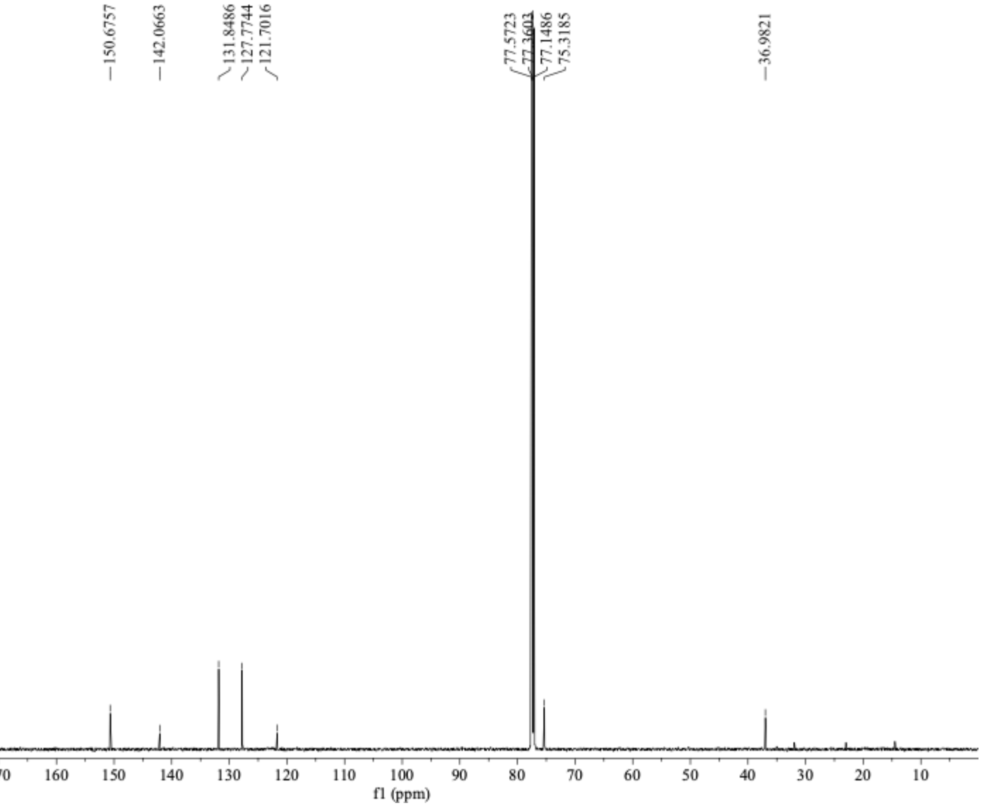

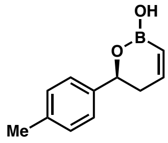

3e ^{1}H NMR
($CDCl_3$, 600 MHz)

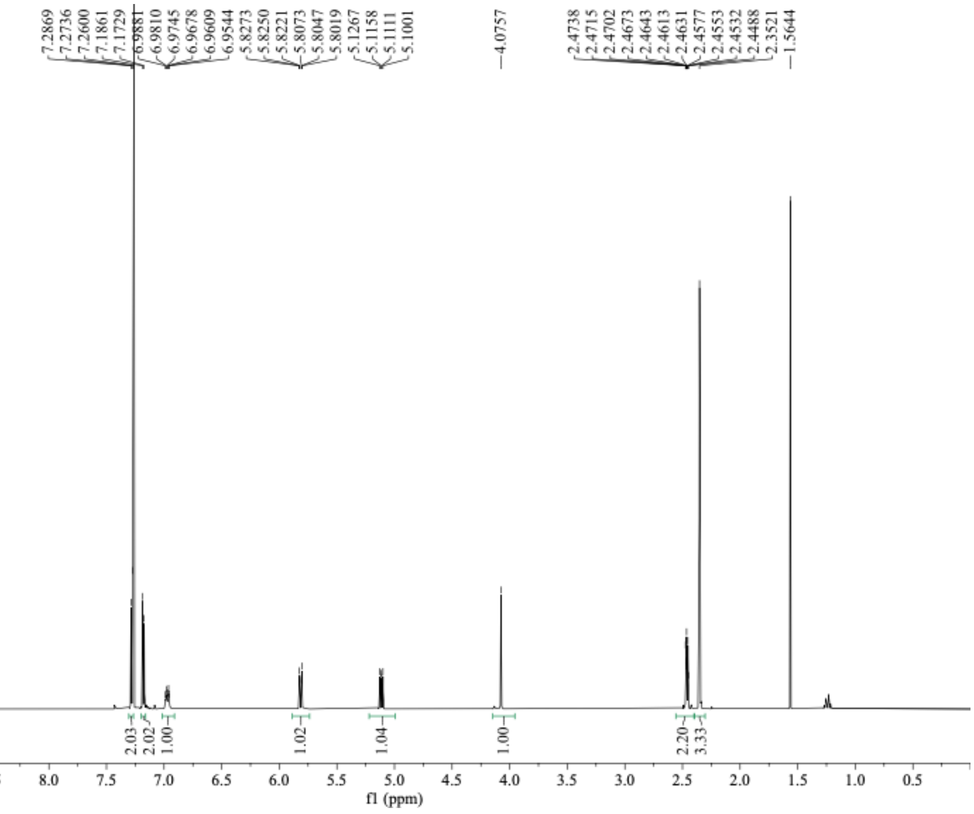

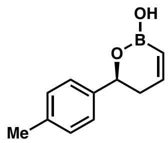

3e ^{13}C NMR
($CDCl_3$, 151 MHz)

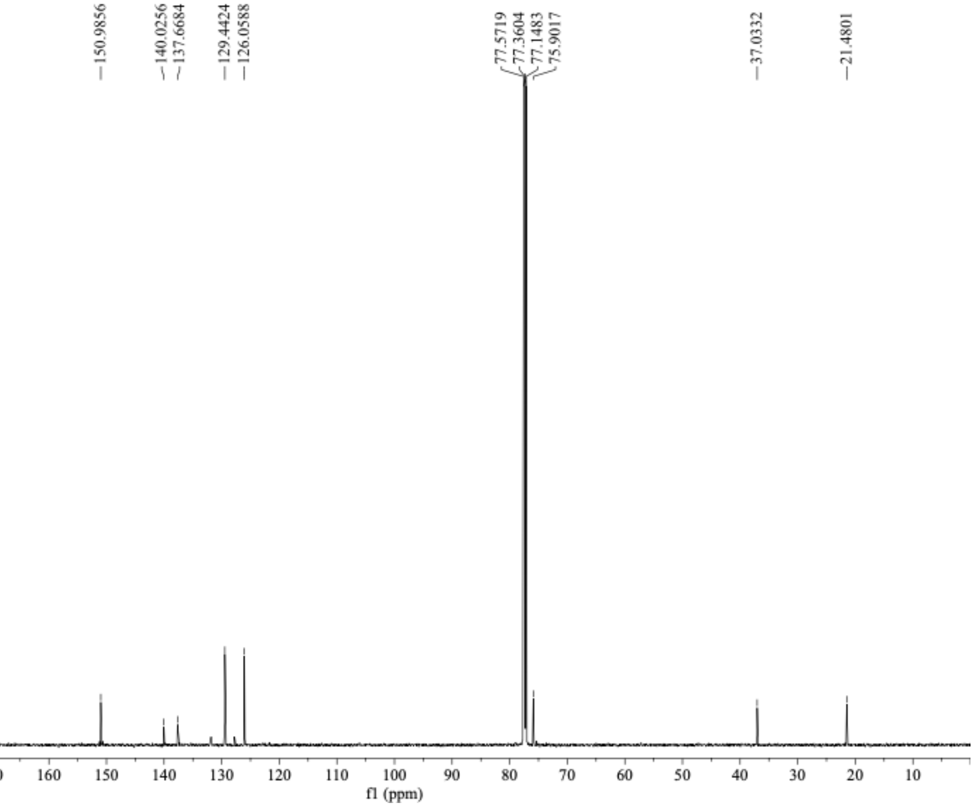

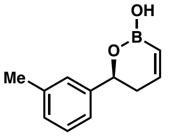

3f ^{1}H NMR
($CDCl_3$, 600 MHz)

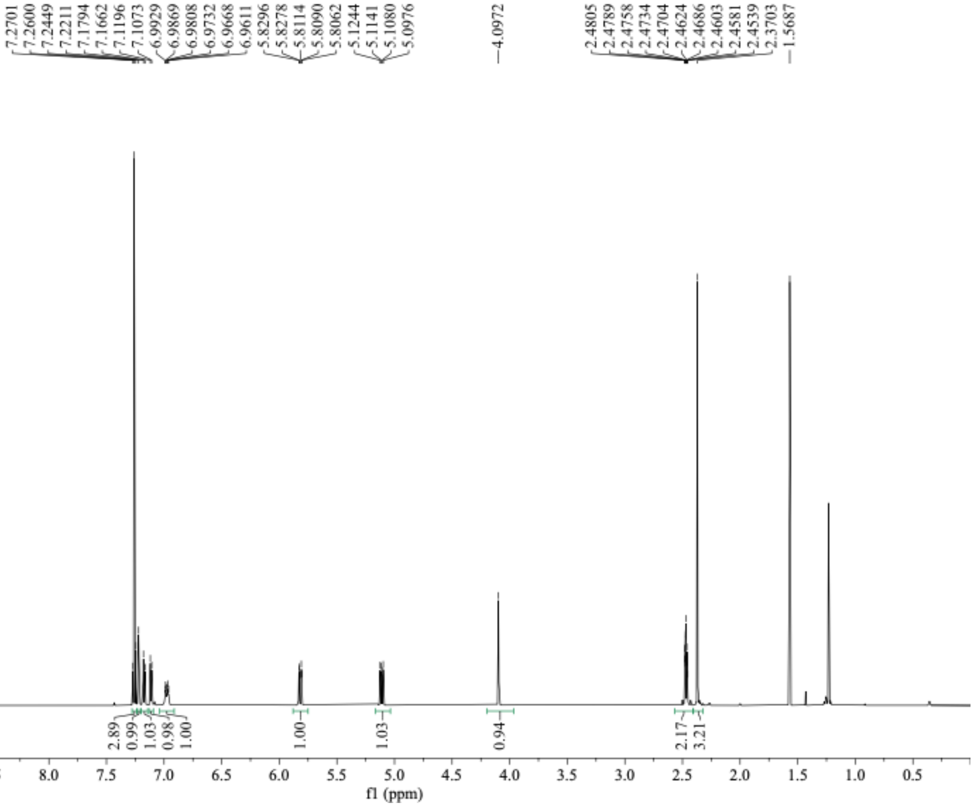

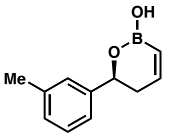

3f ^{13}C NMR
($CDCl_3$, 151 MHz)

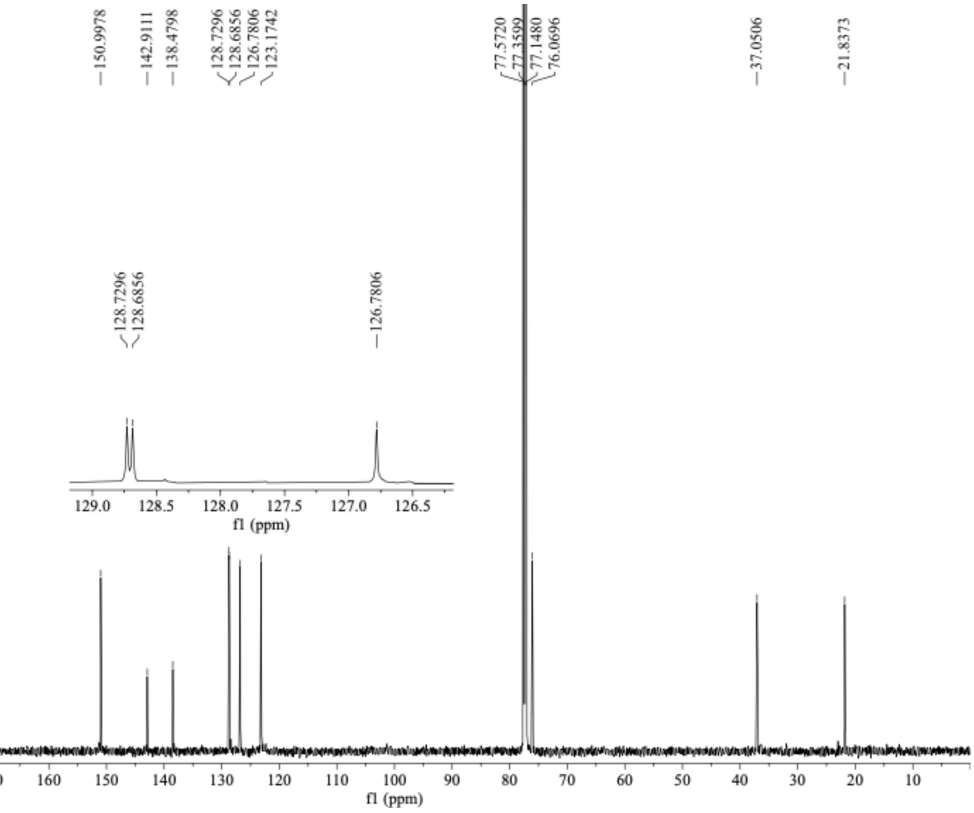

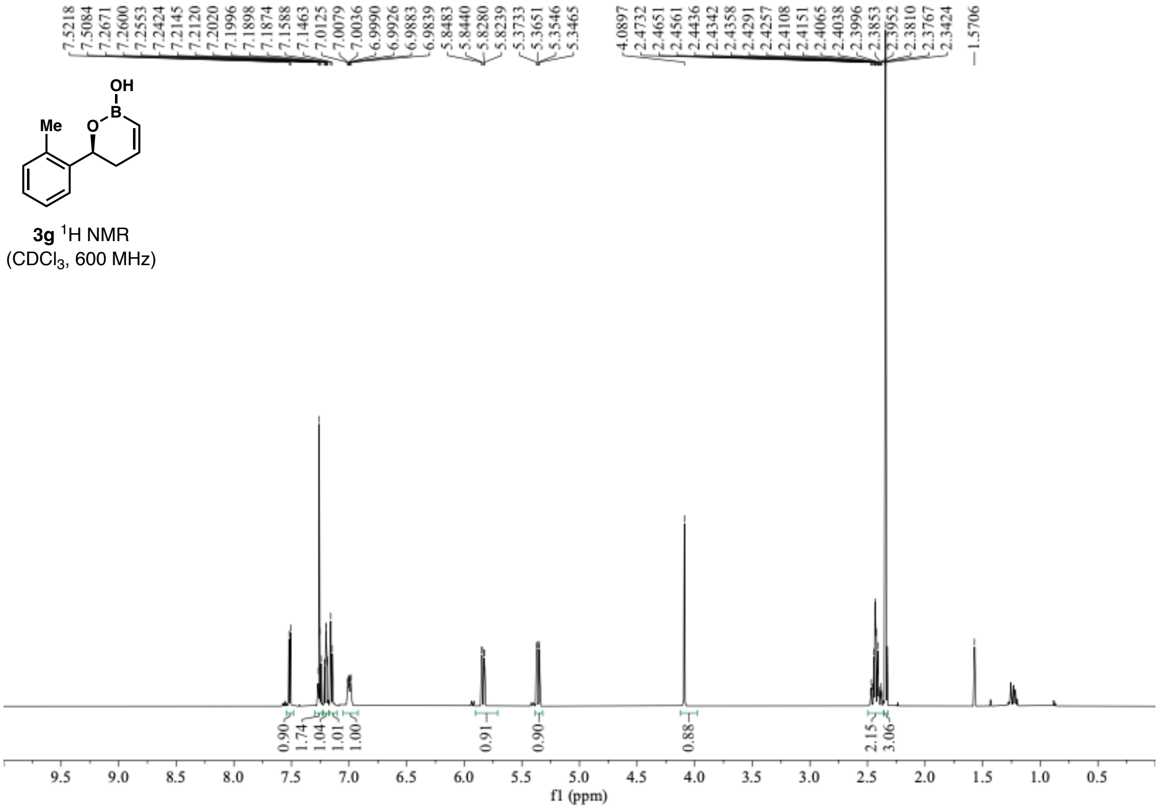

7.5218
7.5084
7.2671
7.2600
7.2553
7.2424
7.2145
7.2120
7.2020
7.1996
7.1898
7.1874
7.1588
7.1463
7.0125
7.0079
7.0036
6.9990
6.9926
6.9883
6.9839
5.8483
5.8440
5.8280
5.8239
5.3733
5.3651
5.3546
5.3465
4.0897
2.4732
2.4651
2.4561
2.4436
2.4358
2.4342
2.4291
2.4257
2.4151
2.4108
2.4065
2.4038
2.3996
2.3952
2.3853
2.3810
2.3767
2.3424
1.5706
OH
B
O
Me
3g ^{1}H NMR
($CDCl_3$, 600 MHz)
0.90
1.74
1.04
1.01
1.00
0.91
0.90
0.88
2.15
3.06
9.5 9.0 8.5 8.0 7.5 7.0 6.5 6.0 5.5 5.0 4.5 4.0 3.5 3.0 2.5 2.0 1.5 1.0 0.5
f1 (ppm)

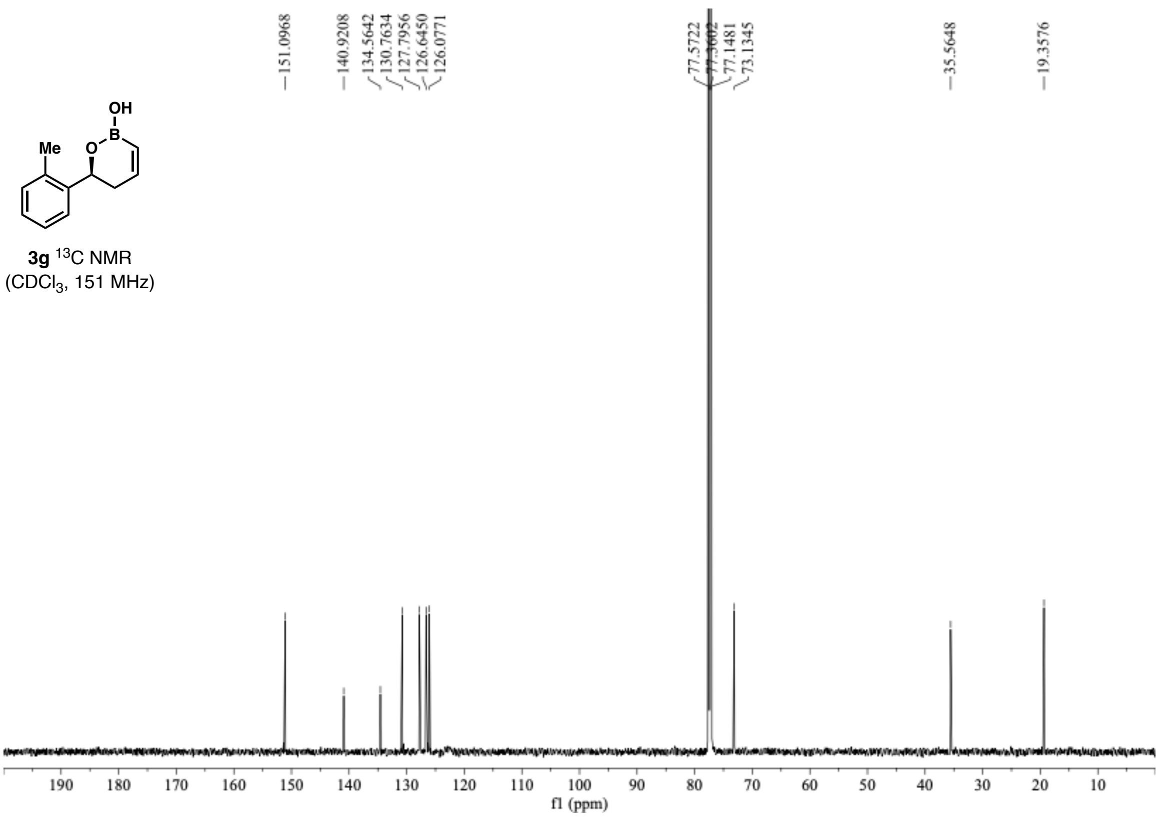

151.0968
140.9208
134.5642
130.7634
127.7956
126.6450
126.0771
77.5722
77.3602
77.1481
73.1345
35.5648
19.3576
OH
B
O
Me
3g ^{13}C NMR
($CDCl_3$, 151 MHz)
190 180 170 160 150 140 130 120 110 100 90 80 70 60 50 40 30 20 10
f1 (ppm)

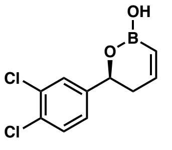

3h ^{1}H NMR
($CDCl_3$, 600 MHz)

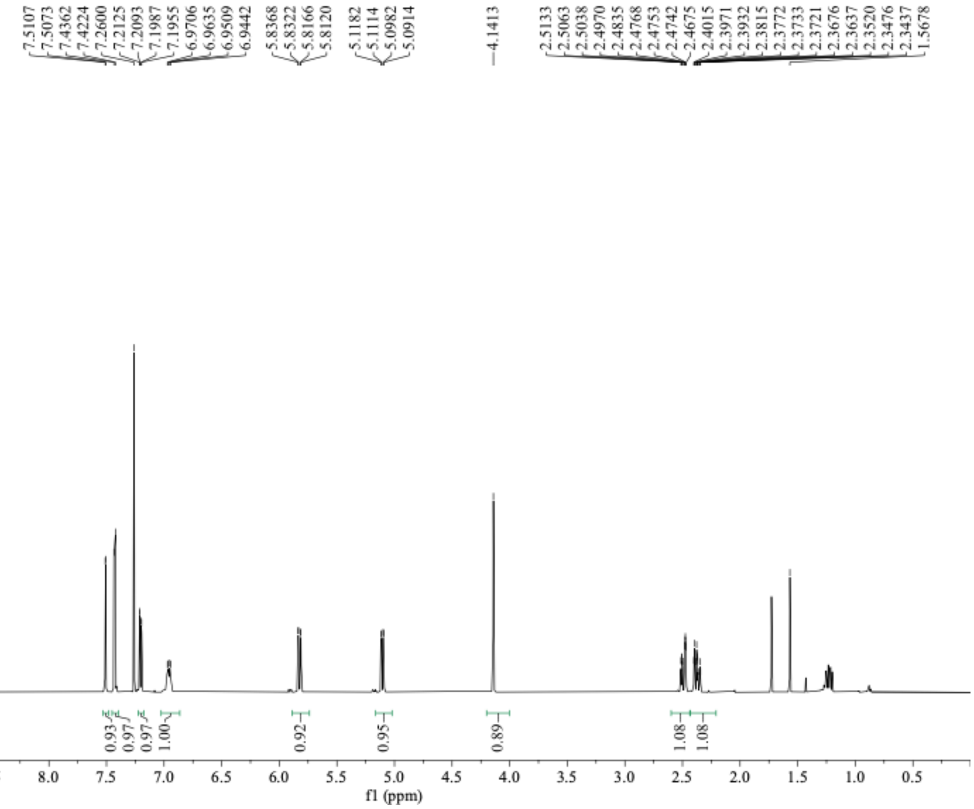

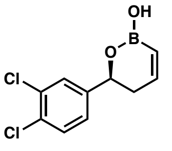

3h ^{13}C NMR
($CDCl_3$, 151 MHz)

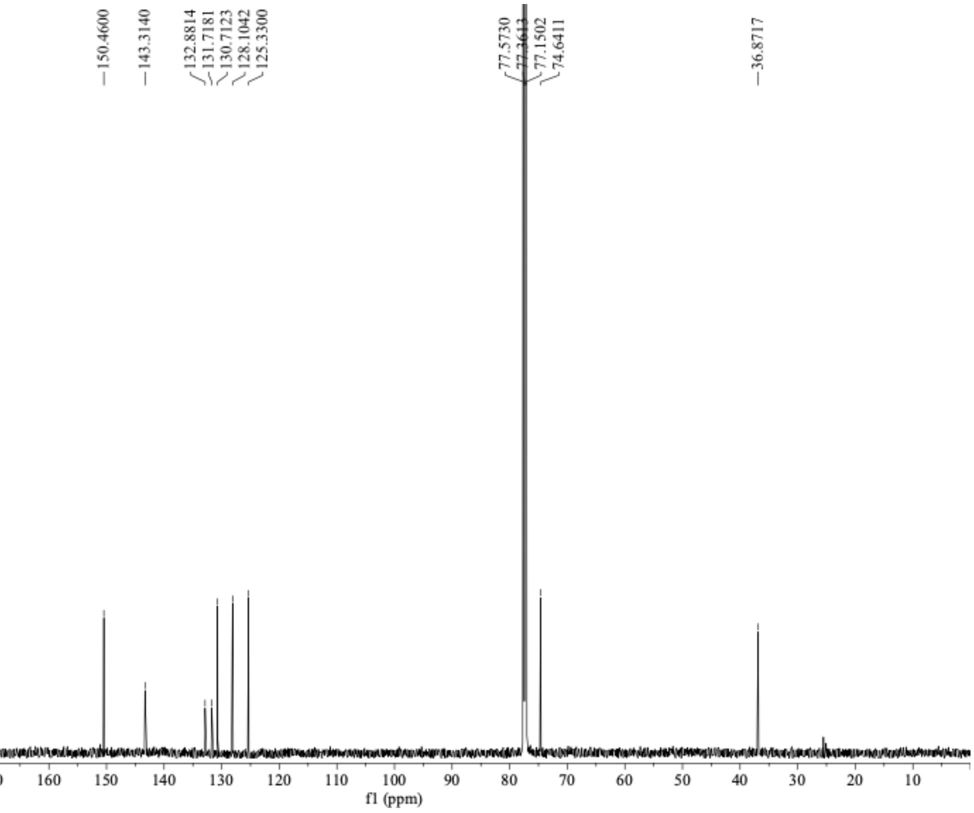

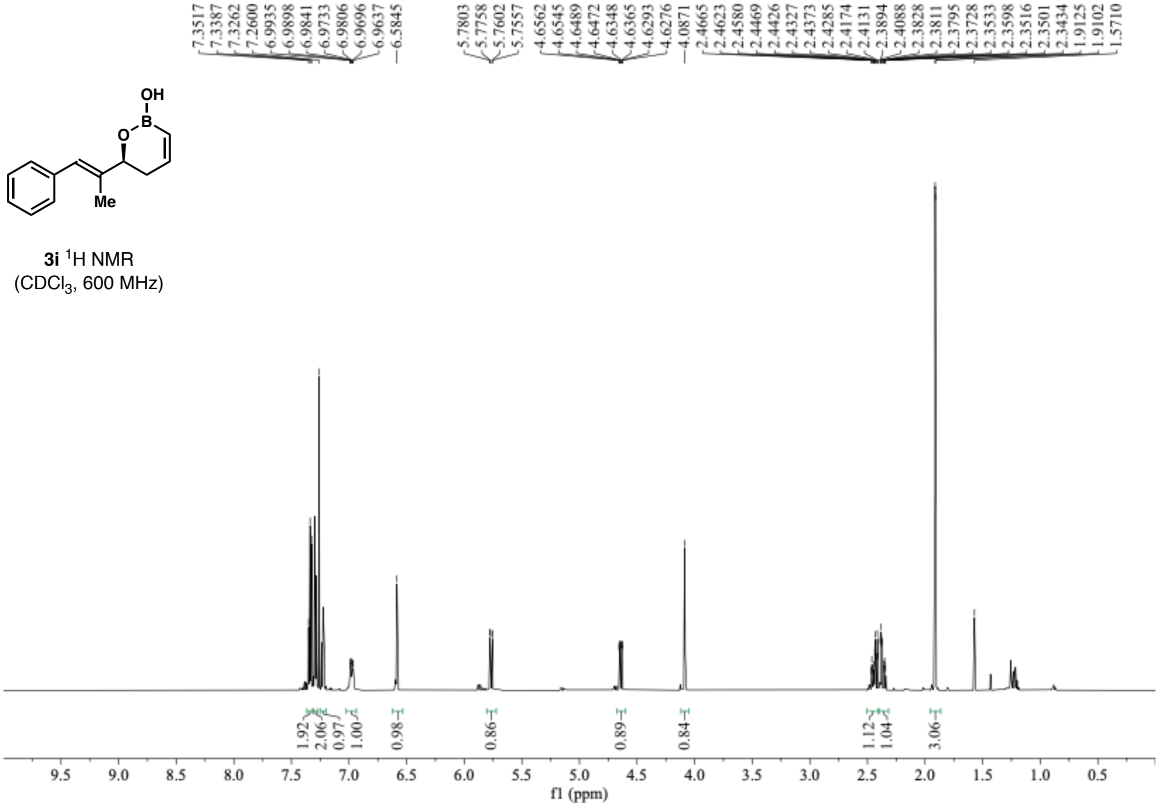

3i ^{1}H NMR
($CDCl_3$, 600 MHz)

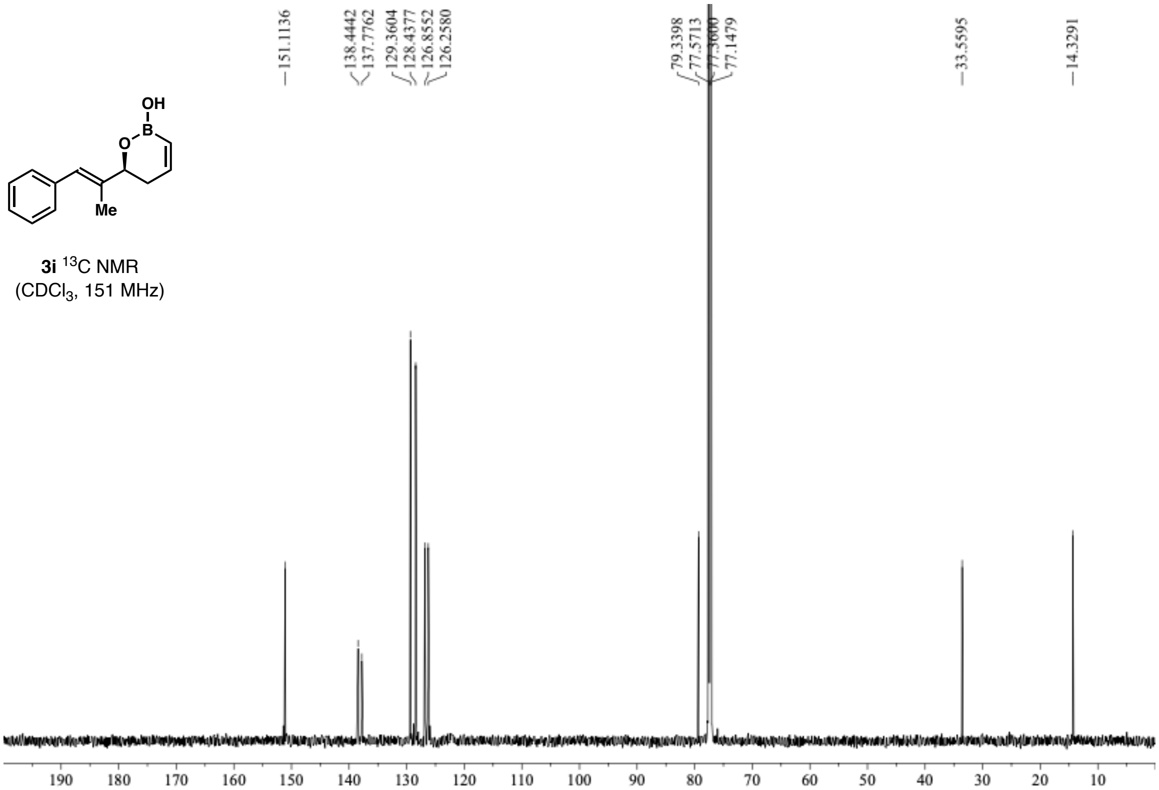

3i ^{13}C NMR
($CDCl_3$, 151 MHz)

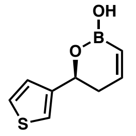

3j ^{1}H NMR
($CDCl_3$, 600 MHz)

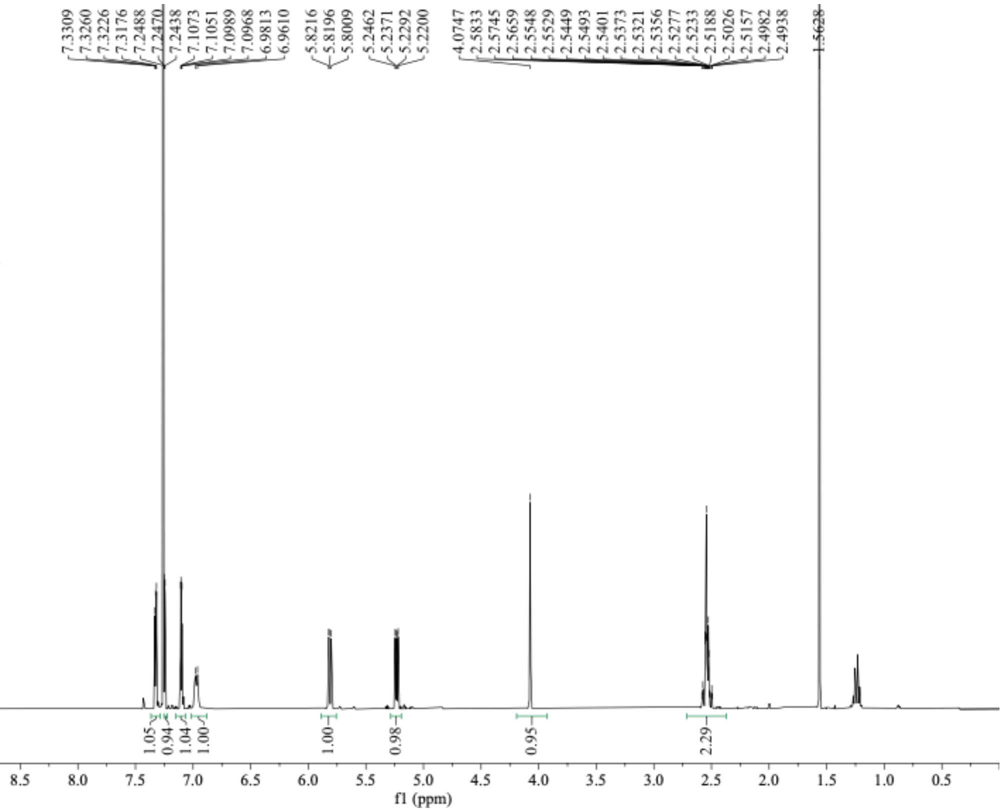

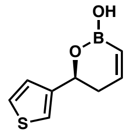

3j ^{13}C NMR
($CDCl_3$, 151 MHz)

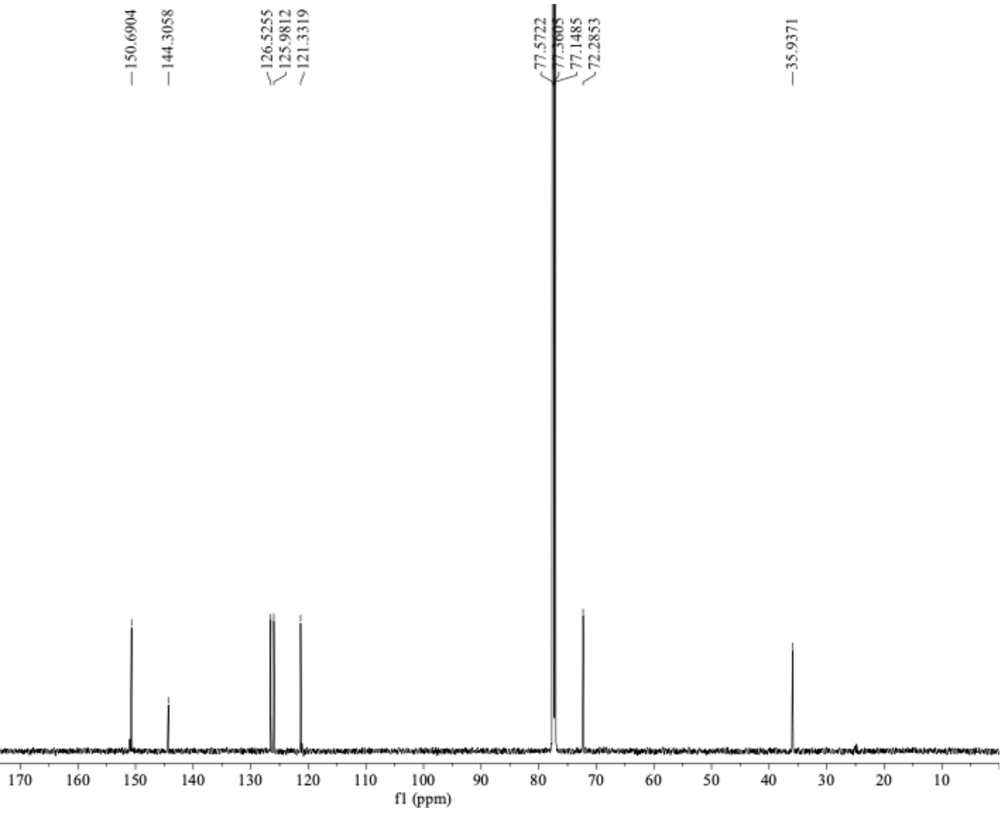

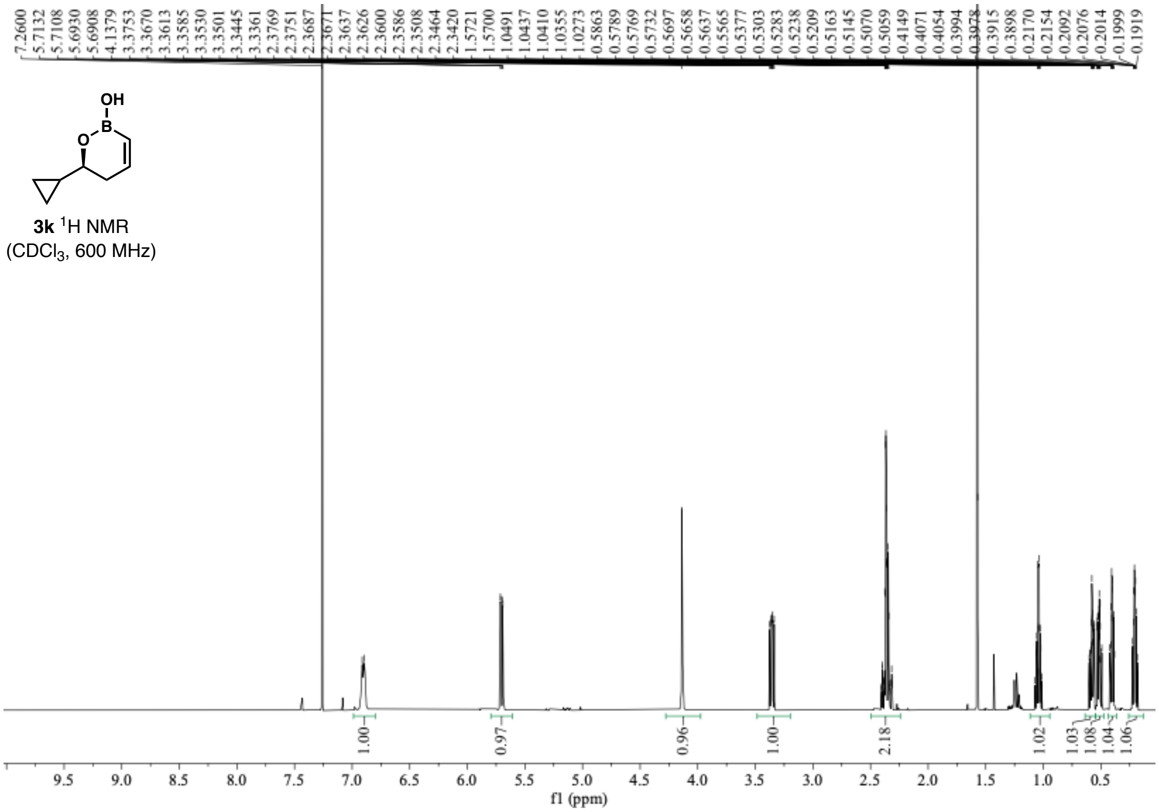

7.2600
5.7132
5.7108
5.6930
5.6908
4.1379
3.3753
3.3670
3.3613
3.3585
3.3530
3.3501
3.3445
3.3361
2.3769
2.3751
2.3687
2.3671
2.3637
2.3626
2.3600
2.3586
2.3508
2.3464
2.3420
1.5721
1.5700
1.0491
1.0437
1.0410
1.0355
1.0273
0.5863
0.5789
0.5769
0.5732
0.5697
0.5658
0.5637
0.5565
0.5377
0.5303
0.5283
0.5238
0.5209
0.5163
0.5145
0.5070
0.5059
0.4149
0.4071
0.4054
0.3994
0.3978
0.3915
0.3898
0.2170
0.2154
0.2092
0.2076
0.2014
0.1999
0.1919
OH
B
O
3k ¹H NMR
(CDCl3, 600 MHz)
1.00
0.97
0.96
1.00
2.18
1.02
1.03
1.08
1.04
1.06
9.5 9.0 8.5 8.0 7.5 7.0 6.5 6.0 5.5 5.0 4.5 4.0 3.5 3.0 2.5 2.0 1.5 1.0 0.5
f1 (ppm)

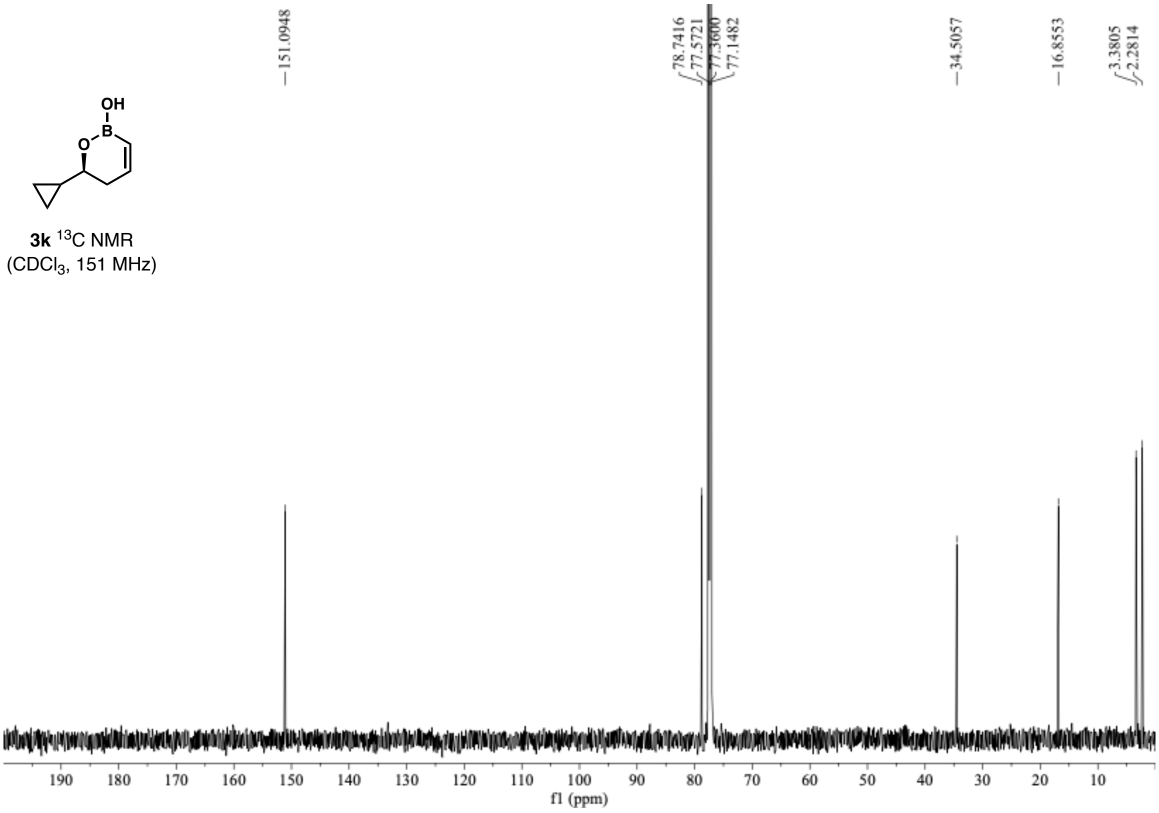

151.0948
78.7416
77.5721
77.3600
77.1482
34.5057
16.8553
3.3805
2.2814
OH
B
O
3k ¹³C NMR
(CDCl3, 151 MHz)
190 180 170 160 150 140 130 120 110 100 90 80 70 60 50 40 30 20 10
f1 (ppm)

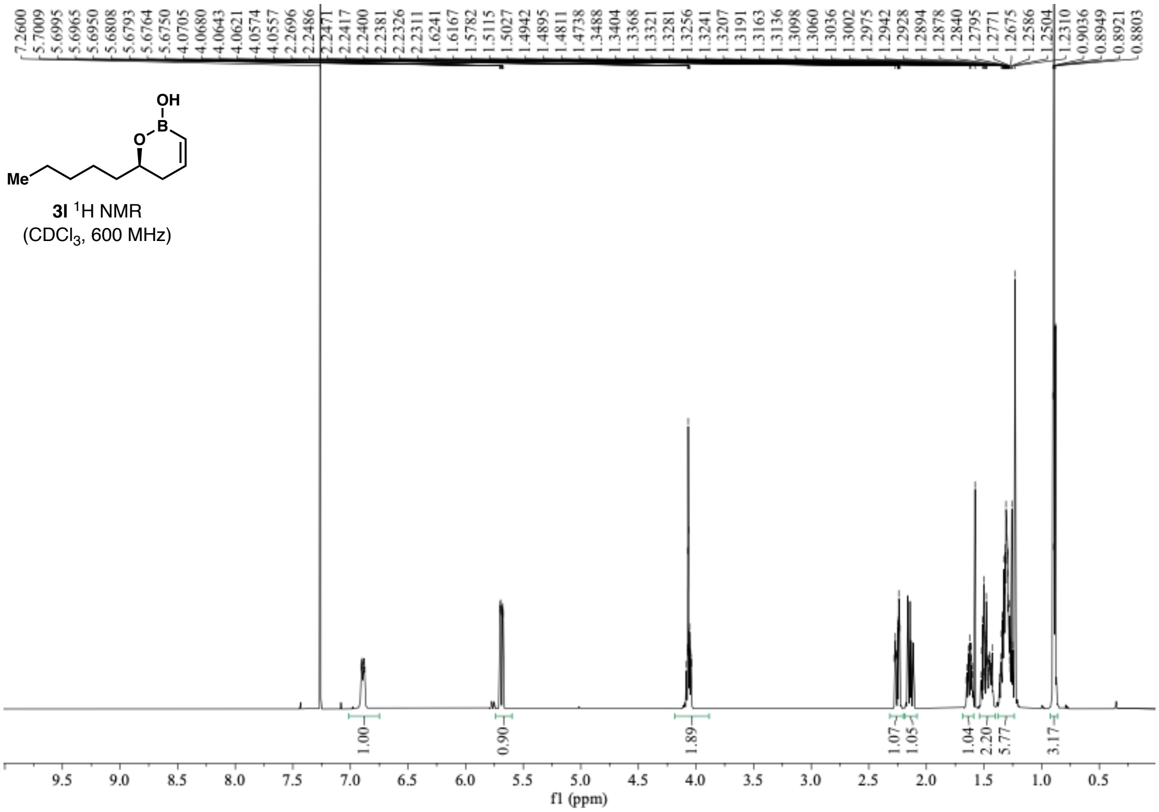

3l ^{1}H NMR
($CDCl_3$, 600 MHz)

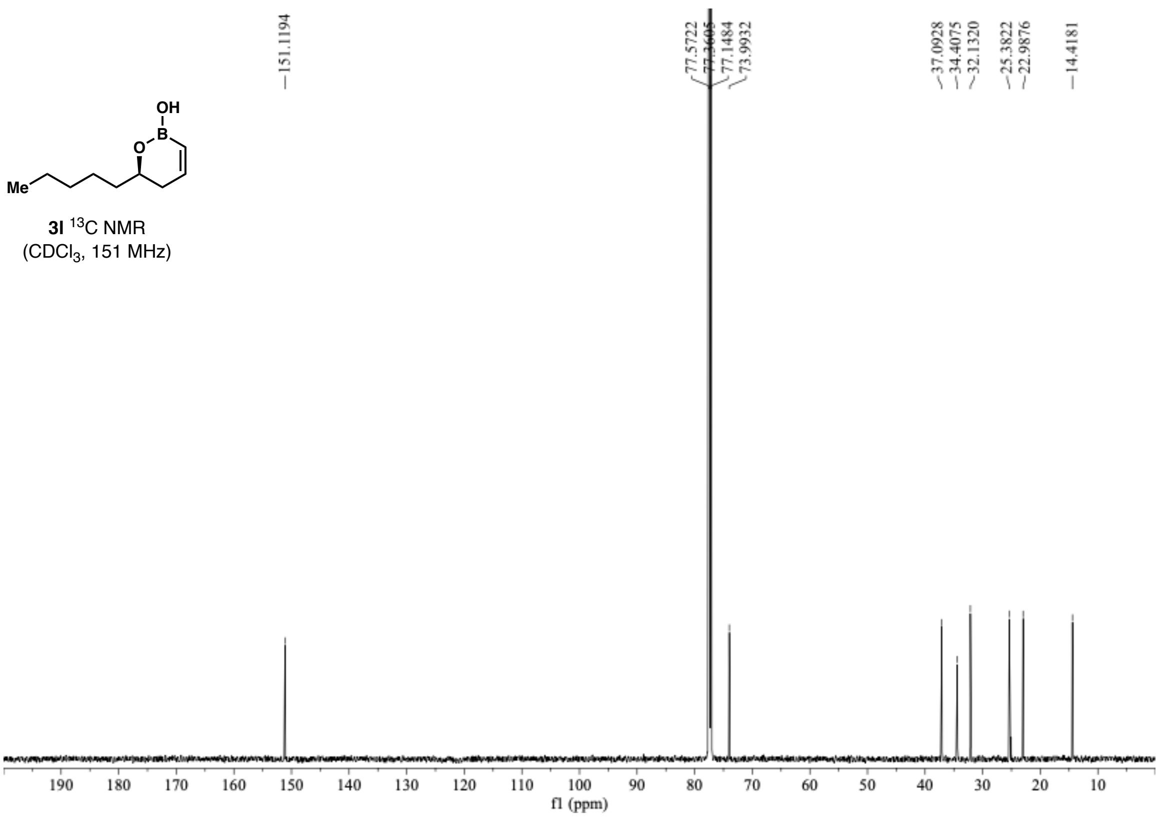

3l ^{13}C NMR
($CDCl_3$, 151 MHz)

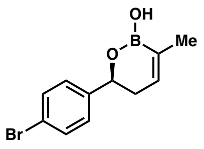

3m ^{1}H NMR
($CDCl_3$, 500 MHz)

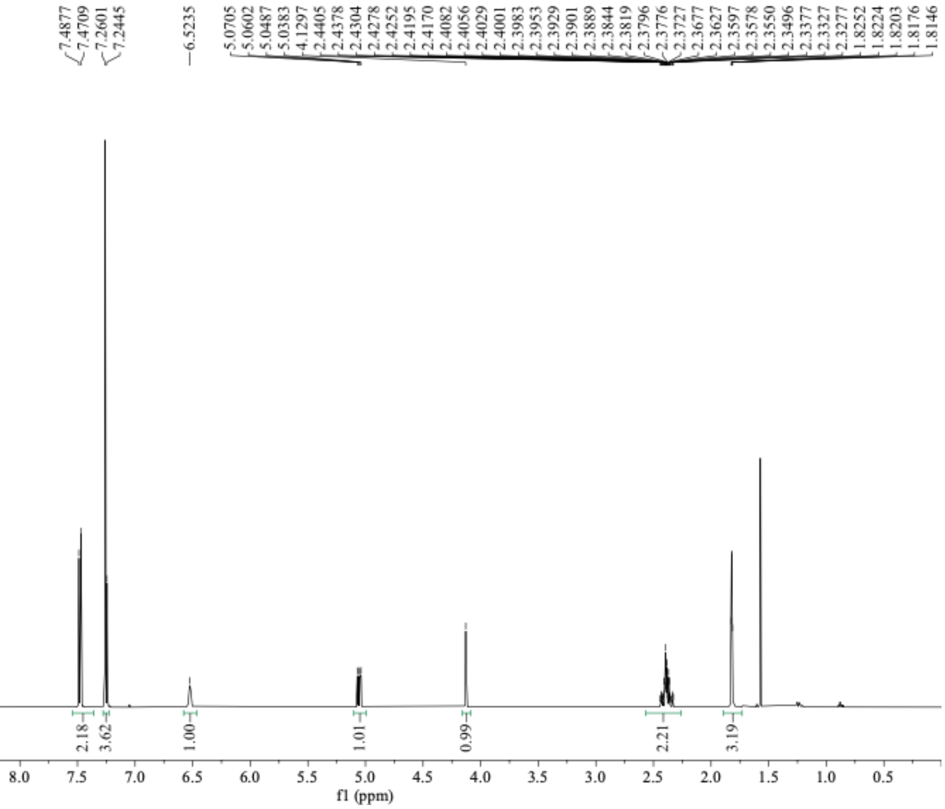

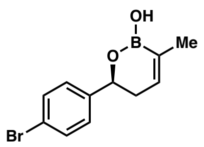

3m ^{13}C NMR
($CDCl_3$, 126 MHz)

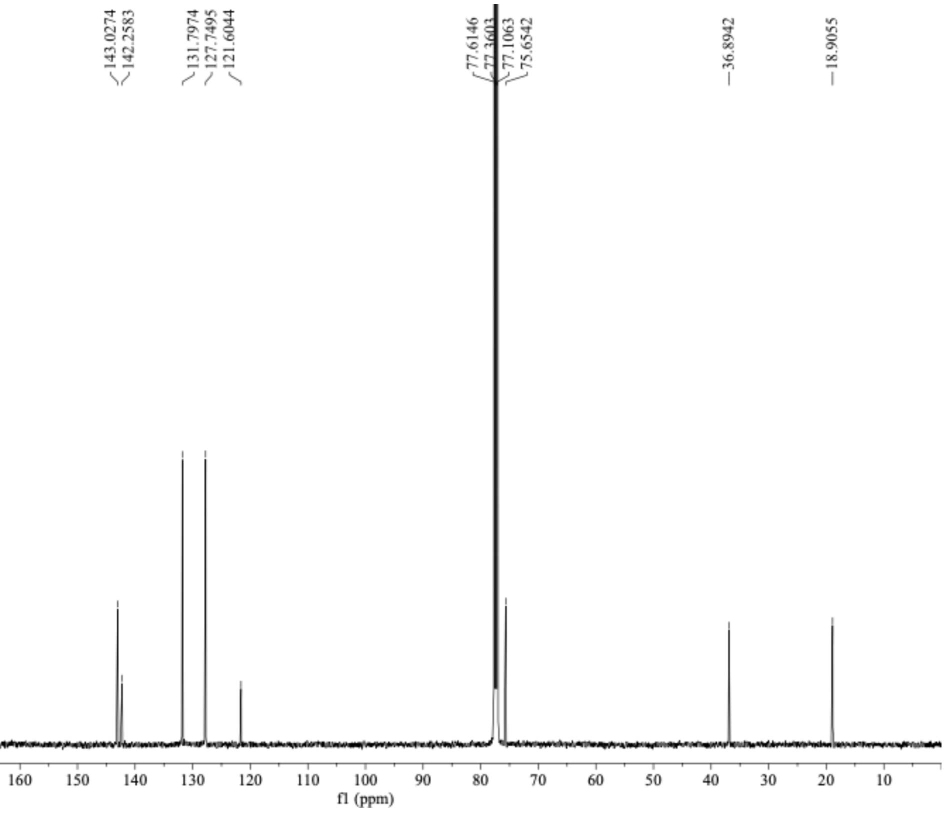

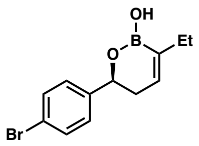

3n ^{1}H NMR
($CDCl_3$, 600 MHz)

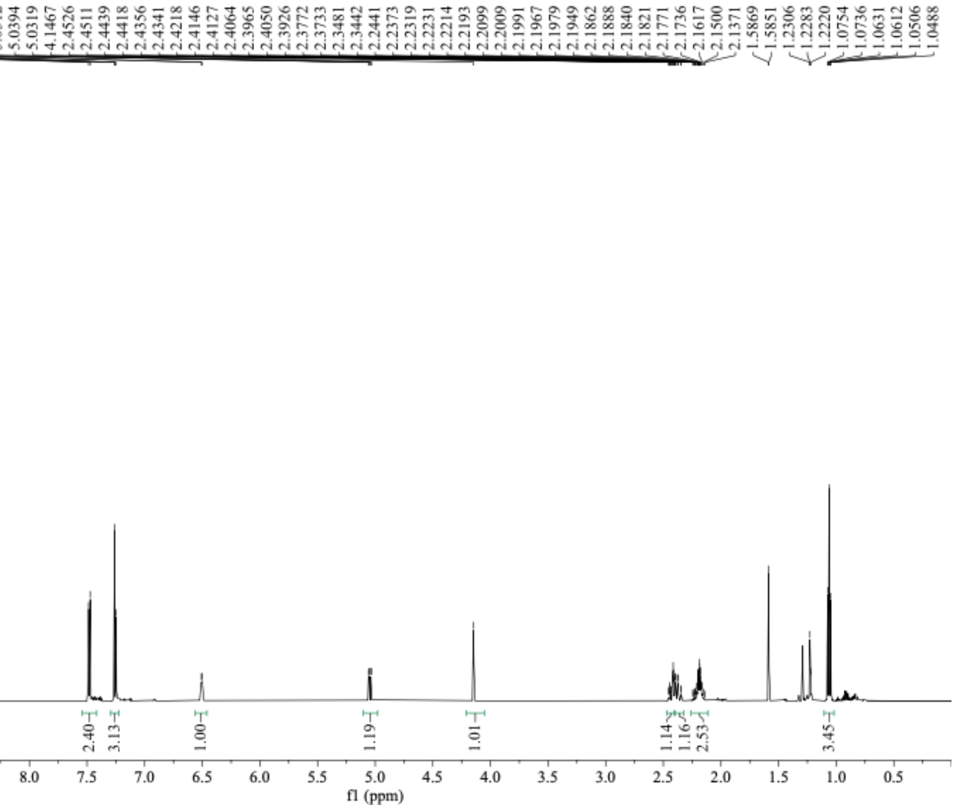

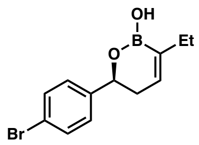

3n ^{13}C NMR
($CDCl_3$, 151 MHz)

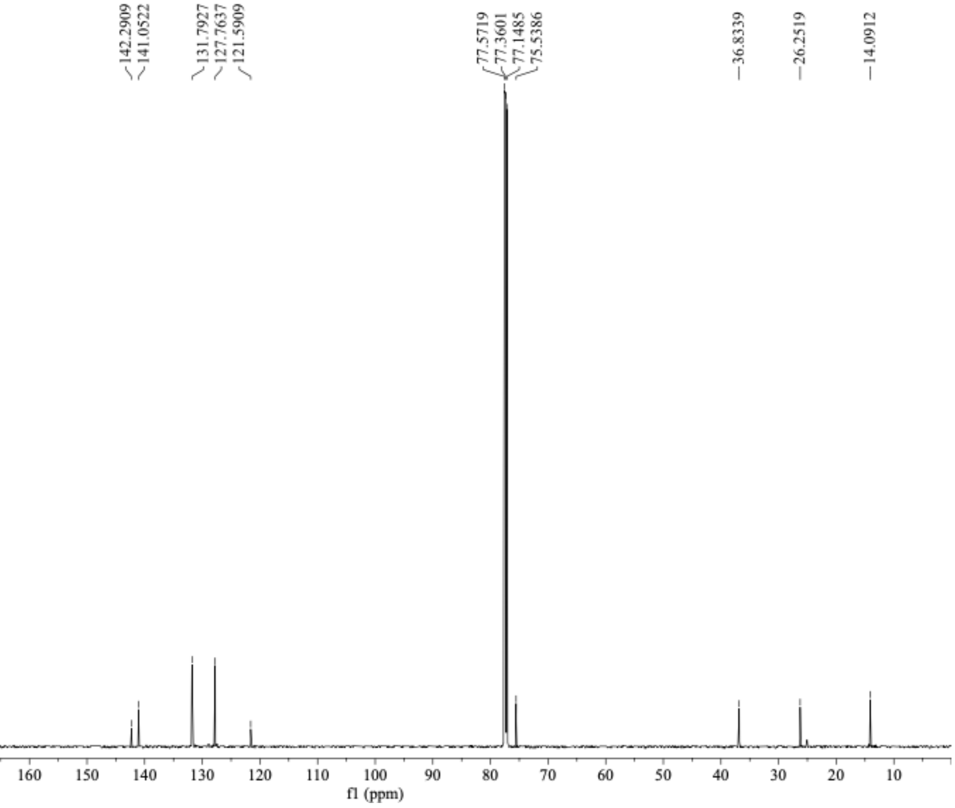

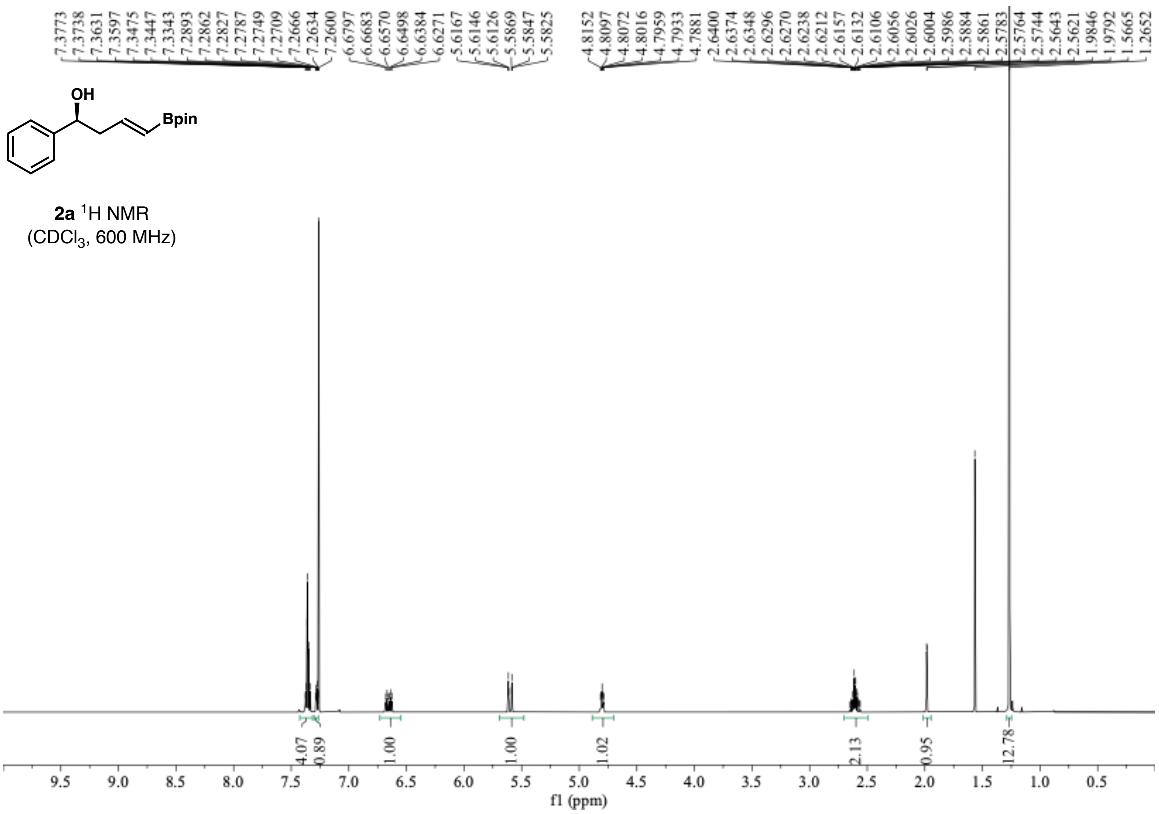
7.3773 7.3738 7.3631 7.3597 7.3475 7.3447 7.3343 7.2893 7.2862 7.2827 7.2787 7.2749 7.2709 7.2666 7.2634 7.2600 6.6797 6.6683 6.6570 6.6498 6.6384 6.6271 5.6167 5.6146 5.6126 5.5869 5.5847 5.5825 4.8152 4.8097 4.8072 4.8016 4.7959 4.7933 4.7881 2.6400 2.6374 2.6348 2.6296 2.6270 2.6238 2.6212 2.6157 2.6132 2.6106 2.6056 2.6026 2.6004 2.5986 2.5884 2.5861 2.5783 2.5764 2.5744 2.5643 2.5621 1.9846 1.9792 1.5665 1.2652
OH
Bpin
2a 1H NMR
(CDCl3, 600 MHz)
4.07 0.89 1.00 1.00 1.02 2.13 0.95 12.78
f1 (ppm)

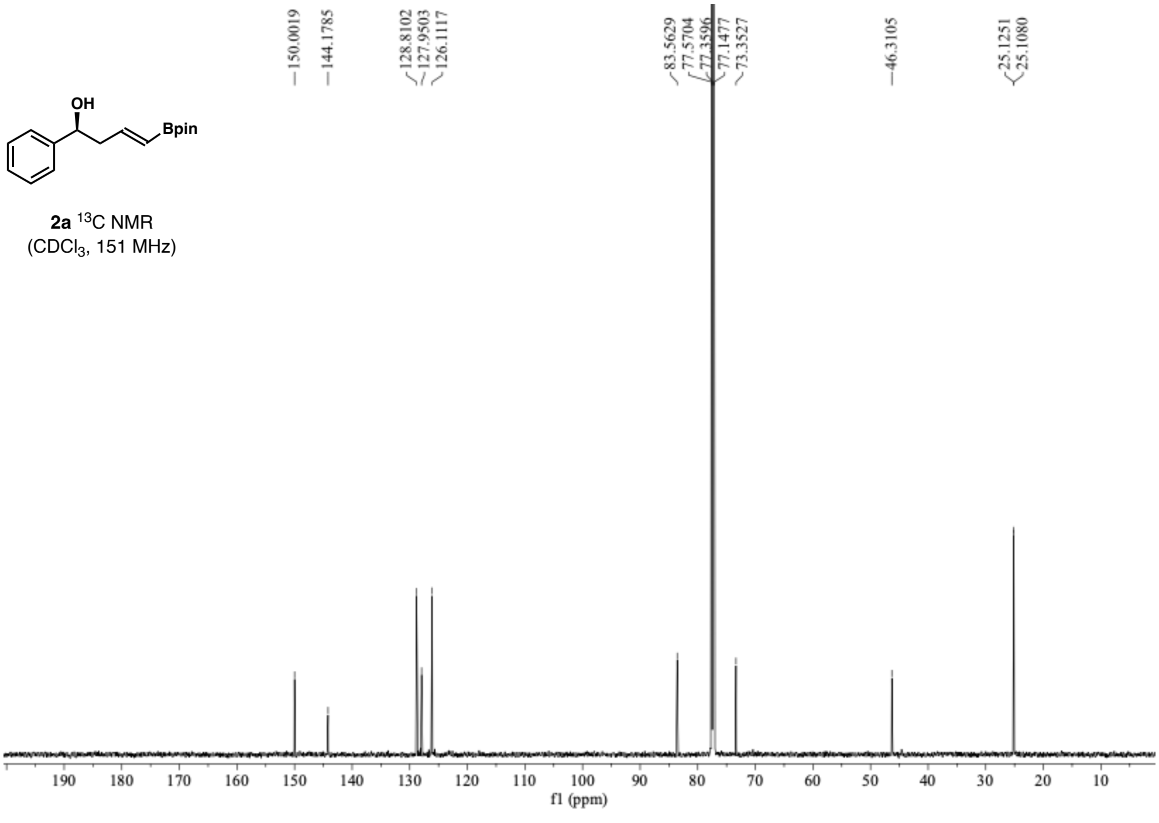
150.0019 144.1785 128.8102 127.9503 126.1117 83.5629 77.5704 77.3596 77.1477 73.3527 46.3105 25.1251 25.1080
OH
Bpin
2a 13C NMR
(CDCl3, 151 MHz)
f1 (ppm)

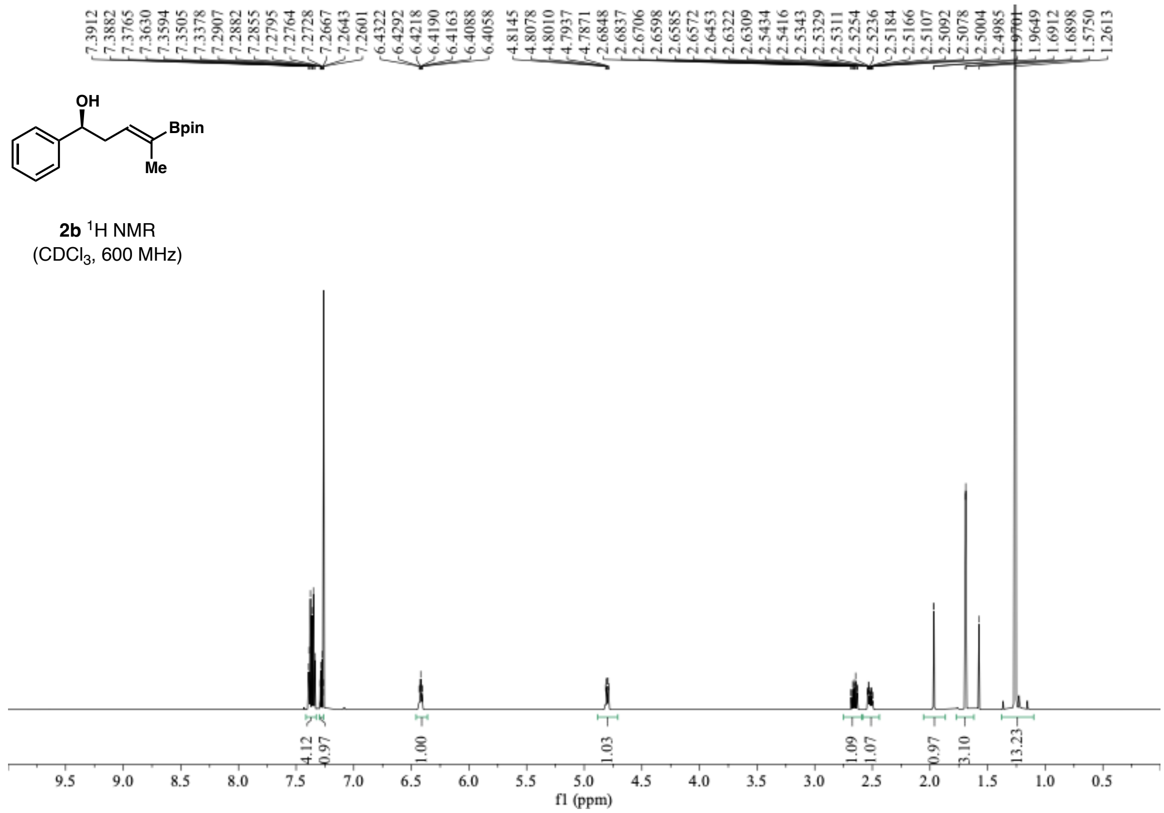

OH
Bpin
Me
2b ¹H NMR
(CDCl₃, 600 MHz)
7.3912 7.3882 7.3765 7.3630 7.3594 7.3505 7.3378 7.2907 7.2882 7.2855 7.2795 7.2764 7.2728 7.2667 7.2643 7.2601 6.4322 6.4292 6.4218 6.4190 6.4163 6.4088 6.4058 4.8145 4.8078 4.8010 4.7937 4.7871 2.6848 2.6837 2.6706 2.6598 2.6585 2.6572 2.6453 2.6322 2.6309 2.5434 2.5416 2.5343 2.5329 2.5311 2.5254 2.5236 2.5184 2.5166 2.5107 2.5092 2.5078 2.5004 2.4985 1.9701 1.9649 1.6912 1.6898 1.5750 1.2613
4.12 0.97 1.00 1.03 1.09 1.07 0.97 3.10 13.23
9.5 9.0 8.5 8.0 7.5 7.0 6.5 6.0 5.5 5.0 4.5 4.0 3.5 3.0 2.5 2.0 1.5 1.0 0.5
f1 (ppm)

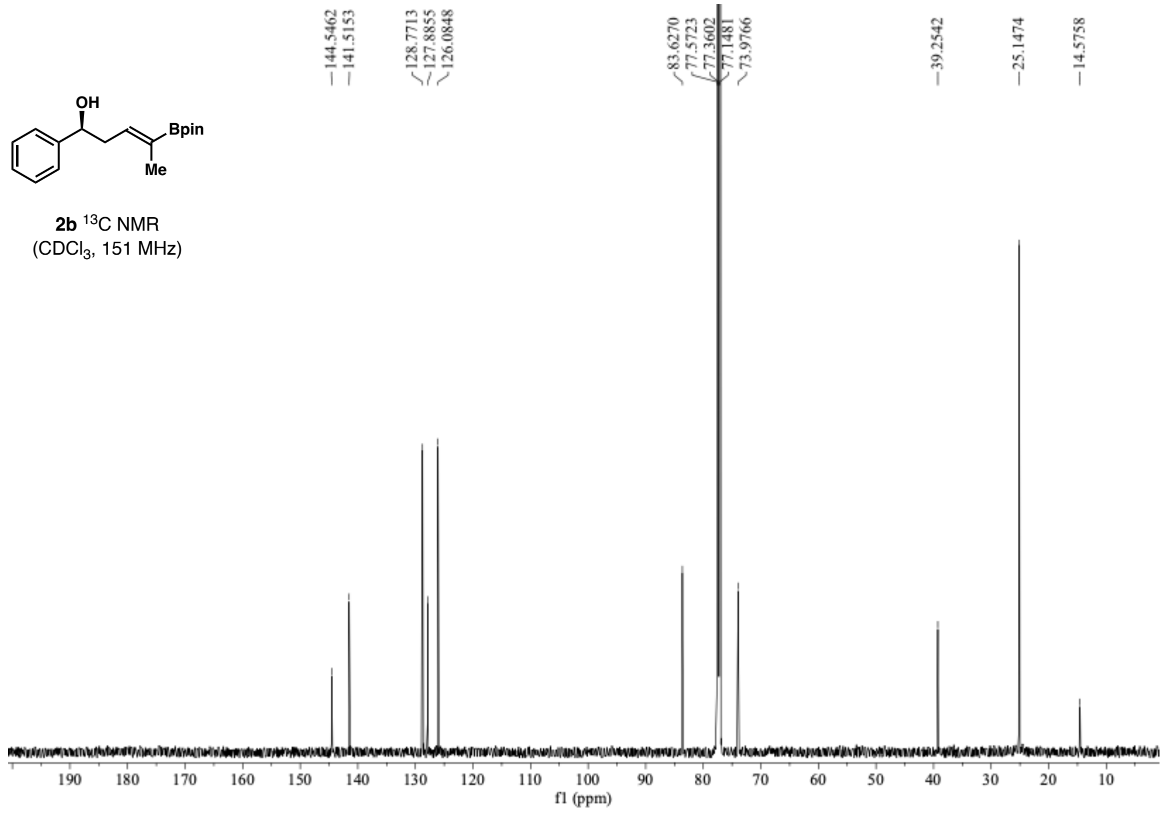

OH
Bpin
Me
2b ¹³C NMR
(CDCl₃, 151 MHz)
144.5462 141.5153 128.7713 127.8855 126.0848 83.6270 77.5723 77.3602 77.1481 73.9766 39.2542 25.1474 14.5758
190 180 170 160 150 140 130 120 110 100 90 80 70 60 50 40 30 20 10
f1 (ppm)

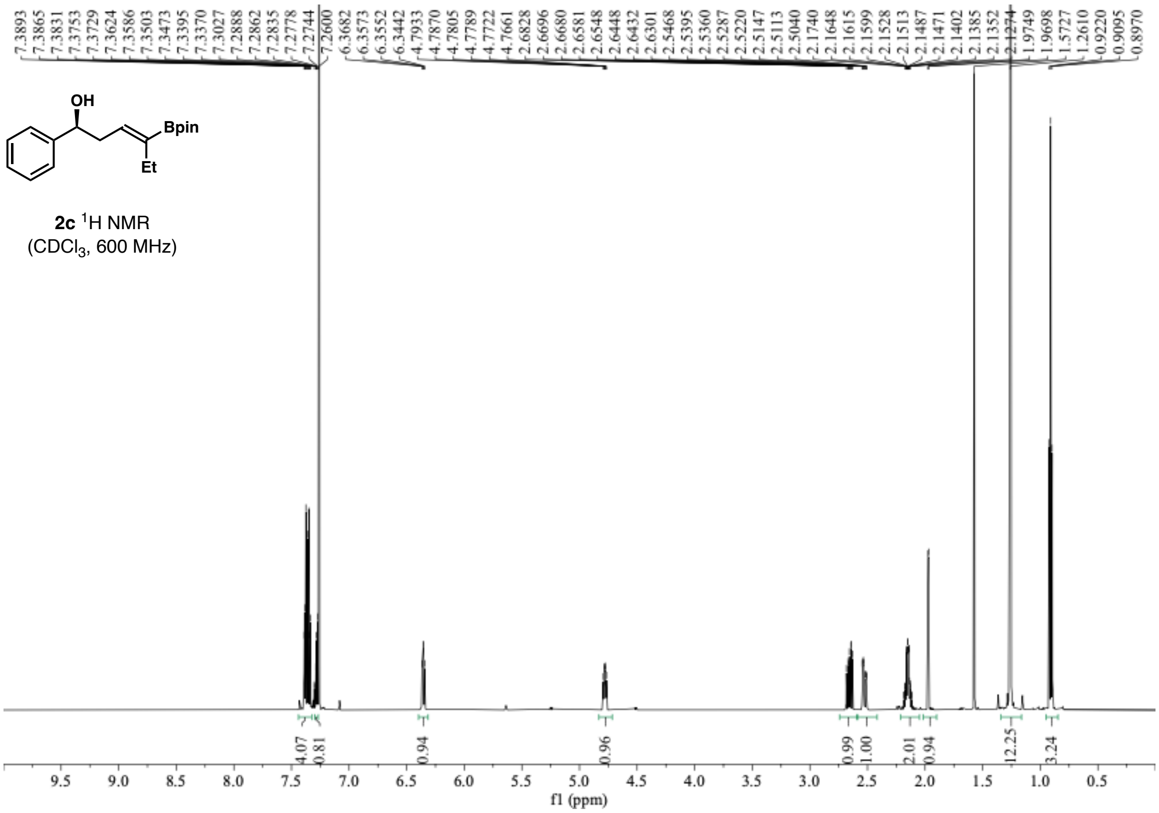
7.3893
7.3865
7.3831
7.3753
7.3729
7.3624
7.3586
7.3503
7.3473
7.3395
7.3370
7.3027
7.2888
7.2862
7.2835
7.2778
7.2744
7.2600
6.3682
6.3573
6.3552
6.3442
4.7933
4.7870
4.7805
4.7789
4.7722
4.7661
2.6828
2.6696
2.6680
2.6581
2.6548
2.6448
2.6432
2.6301
2.5468
2.5395
2.5360
2.5287
2.5220
2.5147
2.5113
2.5040
2.1740
2.1648
2.1615
2.1599
2.1528
2.1513
2.1487
2.1471
2.1402
2.1385
2.1352
2.1274
1.9749
1.9698
1.5727
1.2610
0.9220
0.9095
0.8970
OH
Bpin
Et
2c ¹H NMR
(CDCl3, 600 MHz)
4.07
0.81
0.94
0.96
0.99
1.00
2.01
0.94
12.25
3.24
9.5 9.0 8.5 8.0 7.5 7.0 6.5 6.0 5.5 5.0 4.5 4.0 3.5 3.0 2.5 2.0 1.5 1.0 0.5
f1 (ppm)

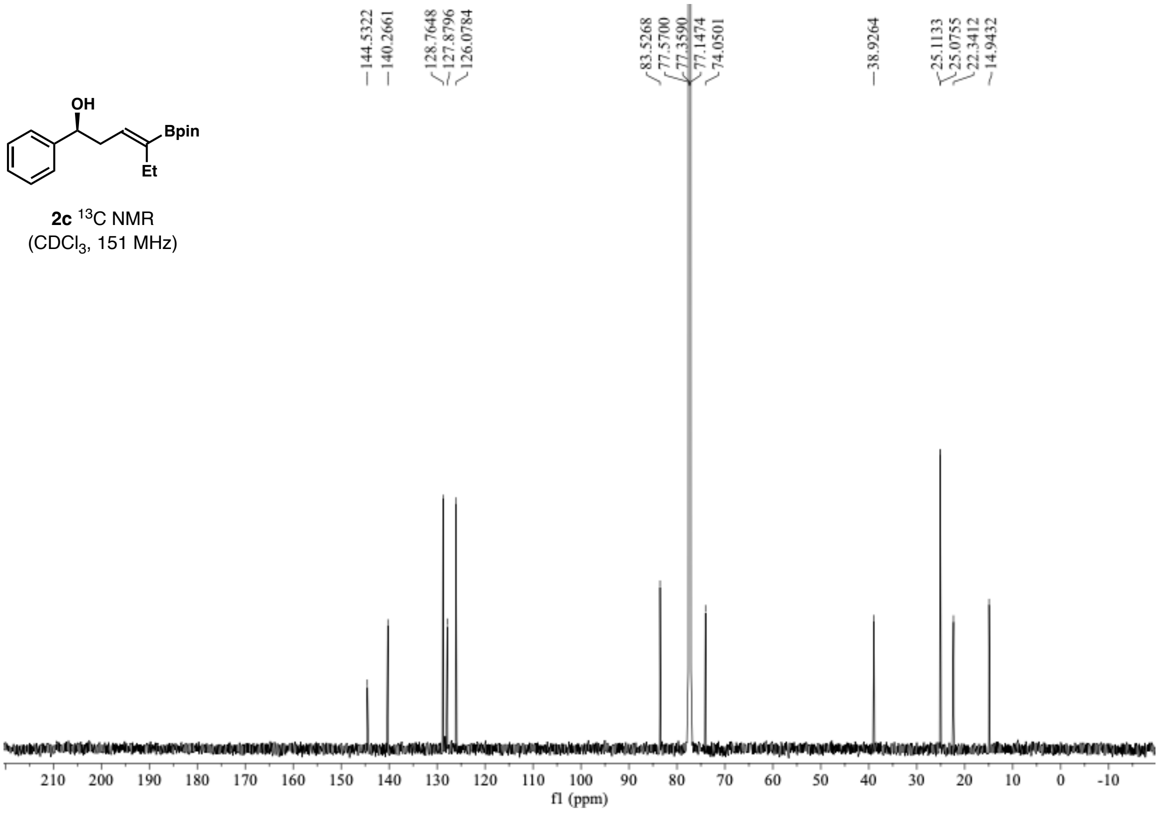
144.5322
140.2661
128.7648
127.8796
126.0784
83.5268
77.5700
77.3590
77.1474
74.0501
38.9264
25.1133
25.0755
22.3412
14.9432
OH
Bpin
Et
2c ¹³C NMR
(CDCl3, 151 MHz)
210 200 190 180 170 160 150 140 130 120 110 100 90 80 70 60 50 40 30 20 10 0 -10
f1 (ppm)

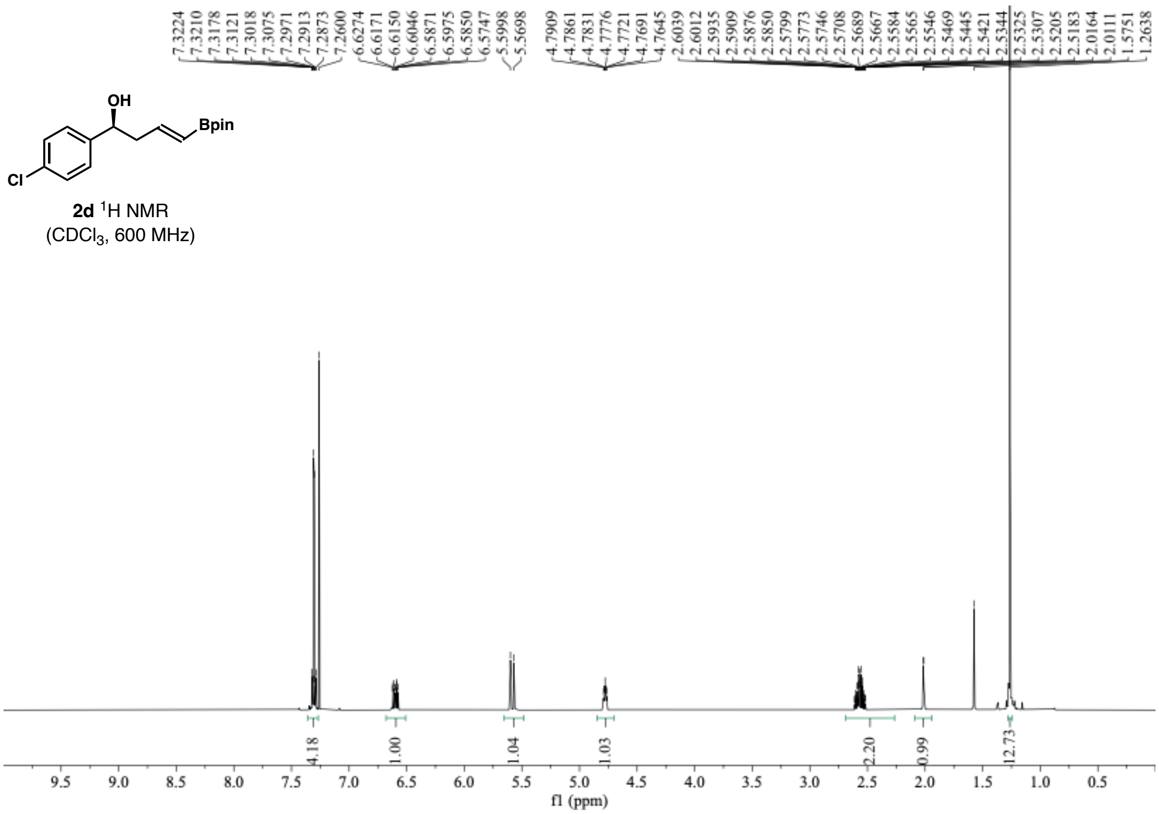

OH
Bpin
Cl
2d ^{1}H NMR
($CDCl_3$, 600 MHz)
7.3224 7.3210 7.3178 7.3121 7.3018 7.3075 7.2971 7.2913 7.2873 7.2600 6.6274 6.6171 6.6150 6.6046 6.5871 6.5975 6.5850 6.5747 5.5998 5.5698 4.7909 4.7861 4.7831 4.7776 4.7721 4.7691 4.7645 2.6039 2.6012 2.5935 2.5909 2.5876 2.5850 2.5799 2.5773 2.5746 2.5708 2.5689 2.5667 2.5584 2.5565 2.5546 2.5469 2.5445 2.5421 2.5344 2.5325 2.5307 2.5205 2.5183 2.0164 2.0111 1.5751 1.2638
4.18
1.00
1.04
1.03
2.20
0.99
12.73
9.5 9.0 8.5 8.0 7.5 7.0 6.5 6.0 5.5 5.0 4.5 4.0 3.5 3.0 2.5 2.0 1.5 1.0 0.5
f1 (ppm)

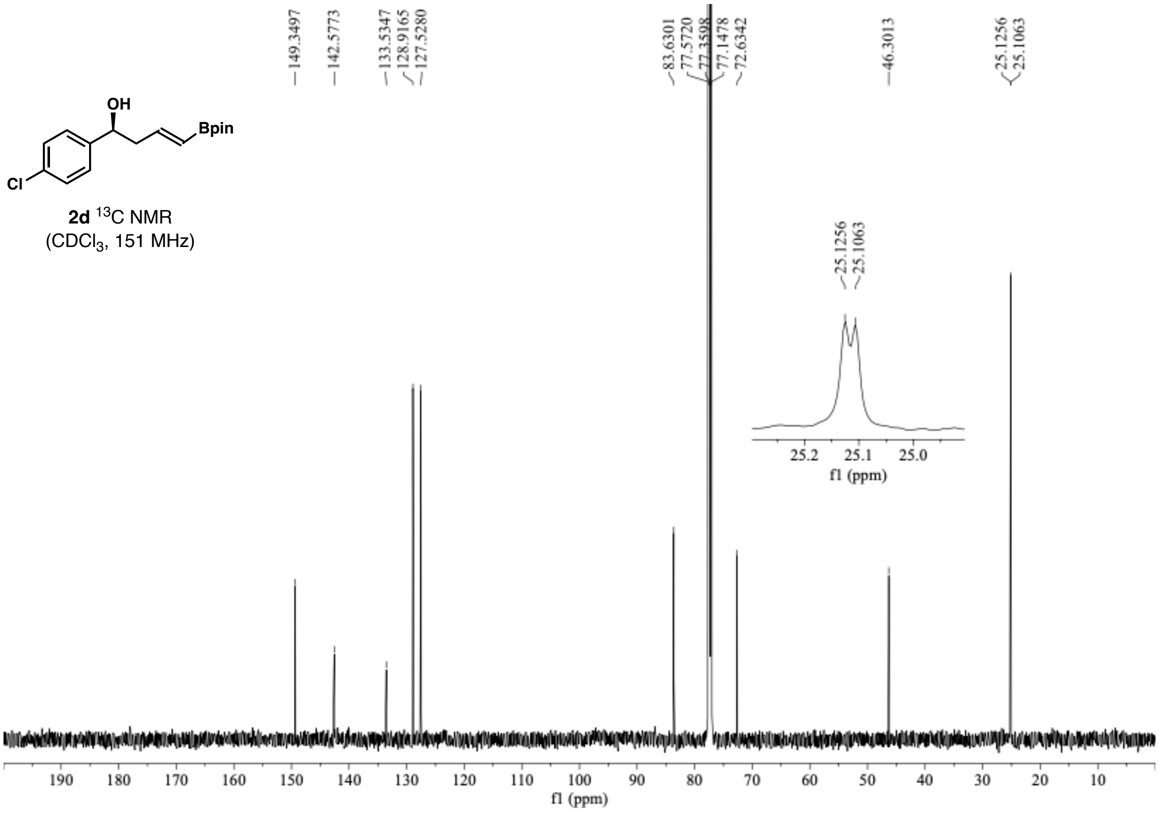

OH
Bpin
Cl
2d ^{13}C NMR
($CDCl_3$, 151 MHz)
149.3497
142.5773
133.5347
128.9165
127.5280
83.6301
77.5720
77.3598
77.1478
72.6342
46.3013
25.1256
25.1063
25.1256
25.1063
25.2 25.1 25.0
f1 (ppm)
190 180 170 160 150 140 130 120 110 100 90 80 70 60 50 40 30 20 10
f1 (ppm)

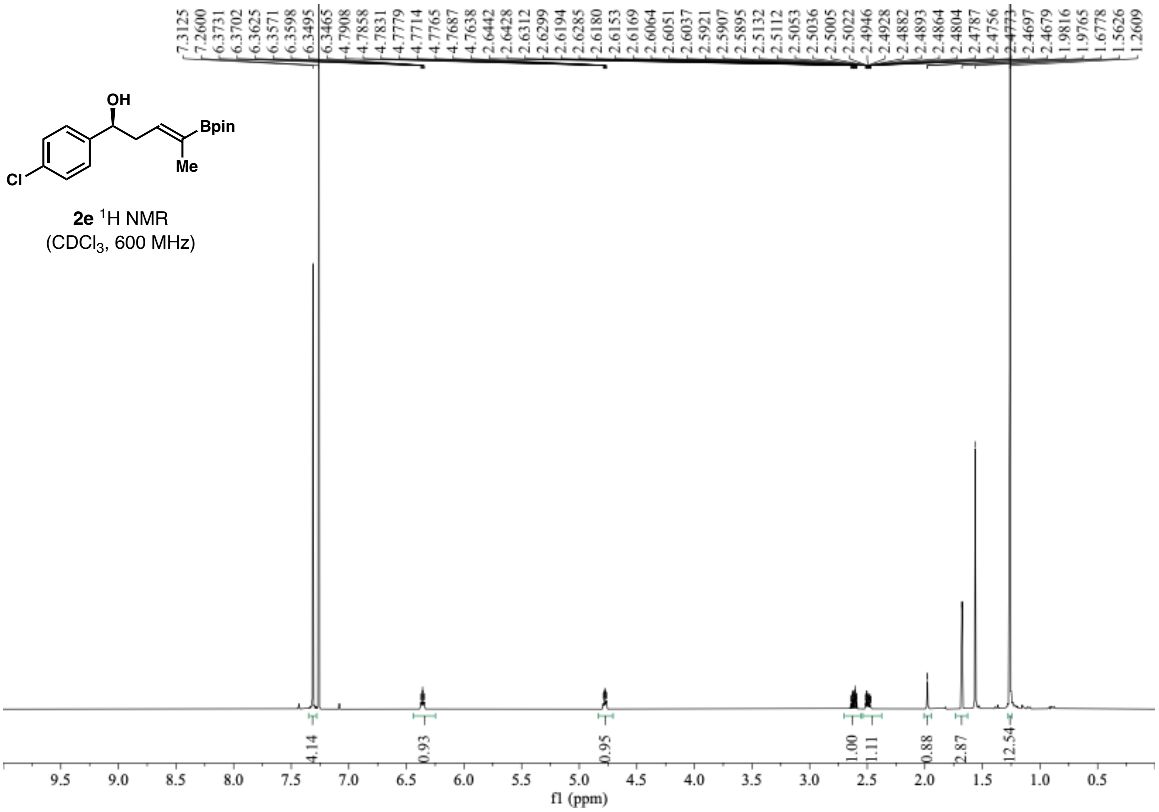

OH
Bpin
Me
Cl
2e ^{1}H NMR
($CDCl_3$, 600 MHz)
7.3125 7.2600 6.3731 6.3702 6.3625 6.3571 6.3598 6.3495 6.3465 4.7908 4.7858 4.7831 4.7779 4.7714 4.7765 4.7687 4.7638 2.6442 2.6428 2.6312 2.6299 2.6194 2.6285 2.6180 2.6153 2.6169 2.6064 2.6051 2.6037 2.5921 2.5907 2.5895 2.5132 2.5112 2.5053 2.5036 2.5005 2.5022 2.4946 2.4928 2.4882 2.4893 2.4864 2.4804 2.4787 2.4756 2.4773 2.4697 2.4679 1.9816 1.9765 1.6778 1.5626 1.2609
4.14 0.93 0.95 1.00 1.11 0.88 2.87 12.54
9.5 9.0 8.5 8.0 7.5 7.0 6.5 6.0 5.5 5.0 4.5 4.0 3.5 3.0 2.5 2.0 1.5 1.0 0.5
f1 (ppm)

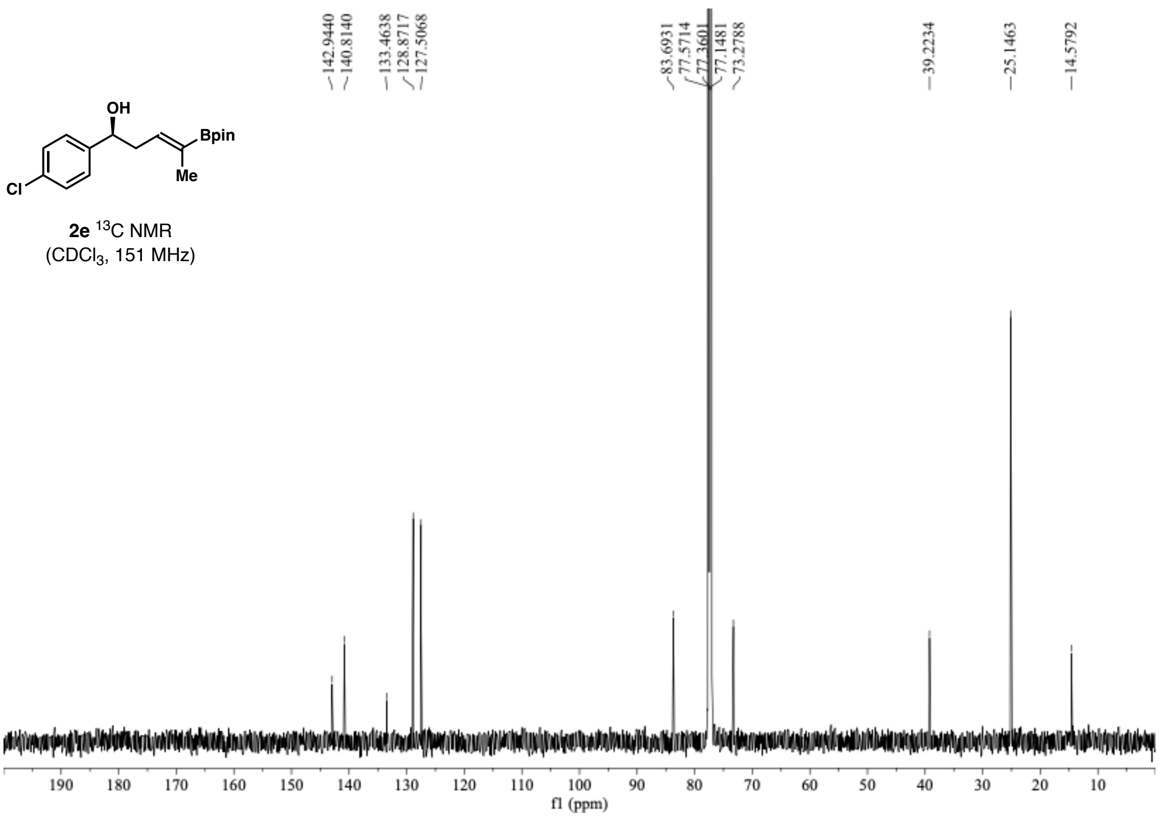

OH
Bpin
Me
Cl
2e ^{13}C NMR
($CDCl_3$, 151 MHz)
142.9440 140.8140 133.4638 128.8717 127.5068 83.6931 77.5714 77.3601 77.1481 73.2788 39.2234 25.1463 14.5792
190 180 170 160 150 140 130 120 110 100 90 80 70 60 50 40 30 20 10
f1 (ppm)

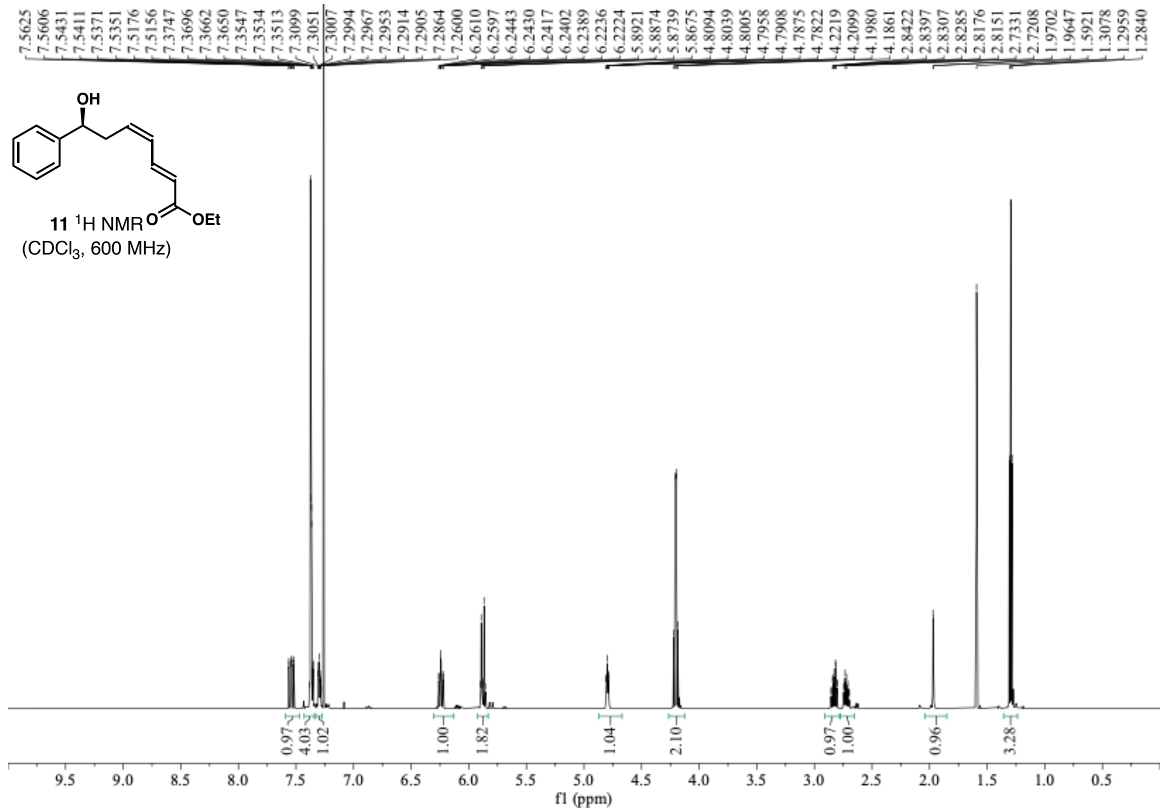

11 ^{1}H NMR (CDCl$_3$, 600 MHz)

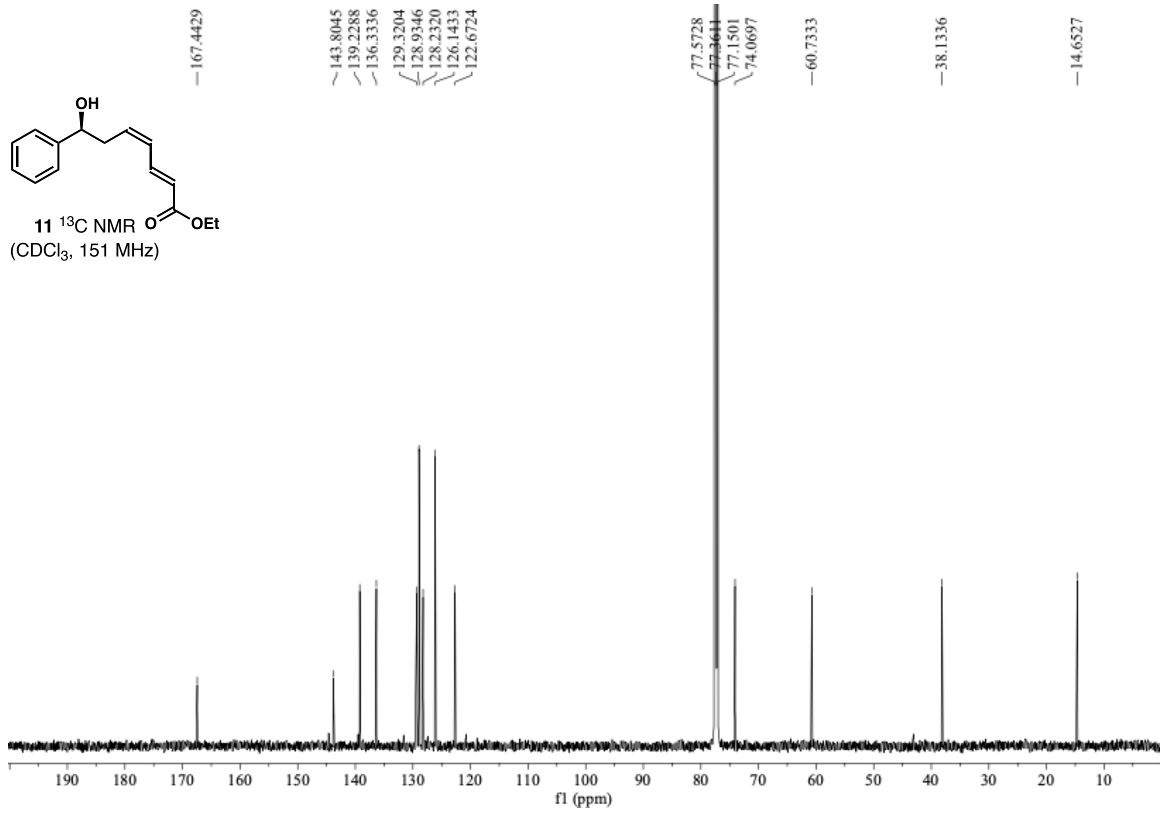

11 ^{13}C NMR (CDCl$_3$, 151 MHz)

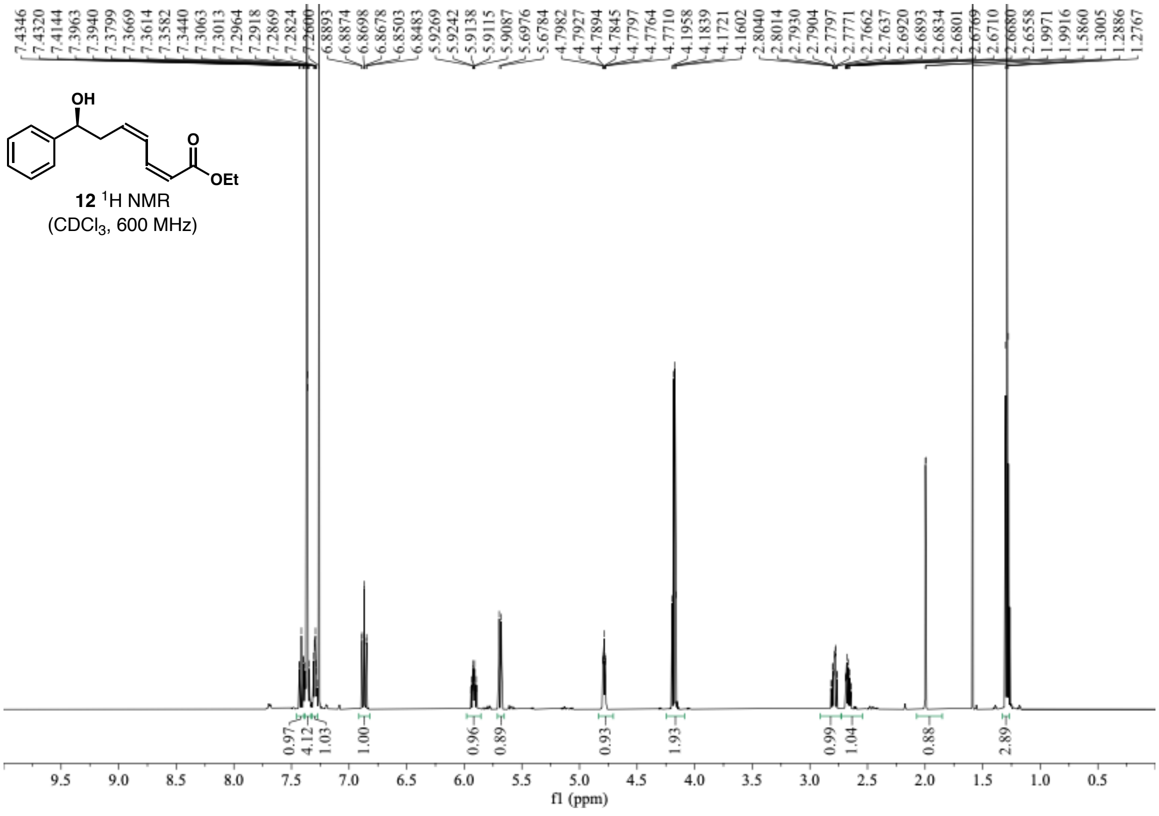
OH
OEt
12 1H NMR
(CDCl3, 600 MHz)
f1 (ppm)

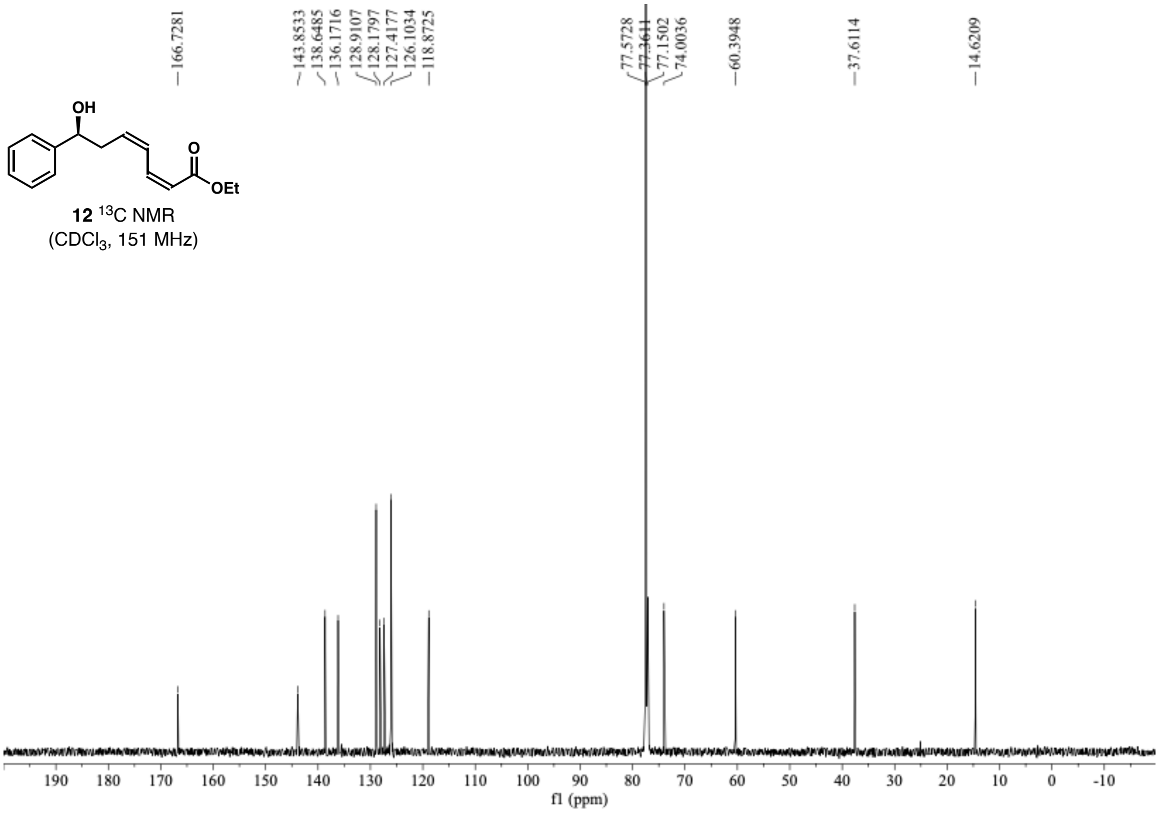
OH
OEt
12 13C NMR
(CDCl3, 151 MHz)
f1 (ppm)

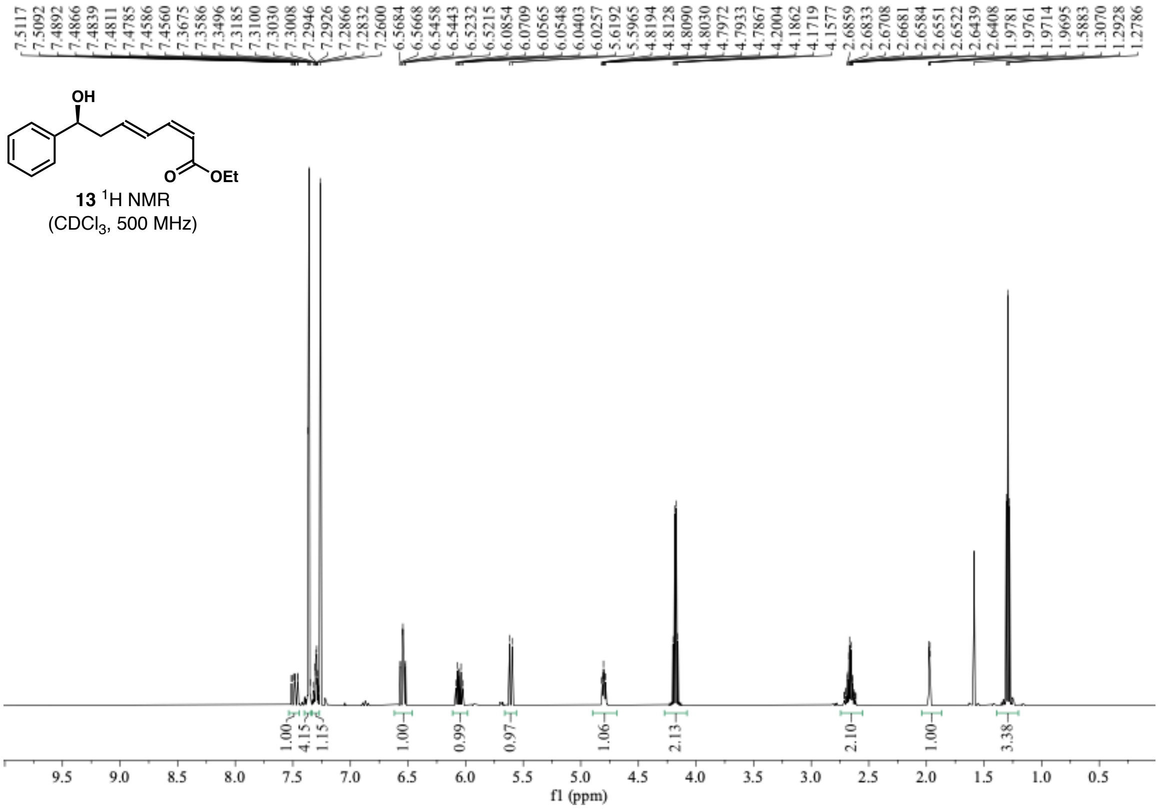
OH
O
OEt
13 1H NMR
(CDCl3, 500 MHz)
f1 (ppm)

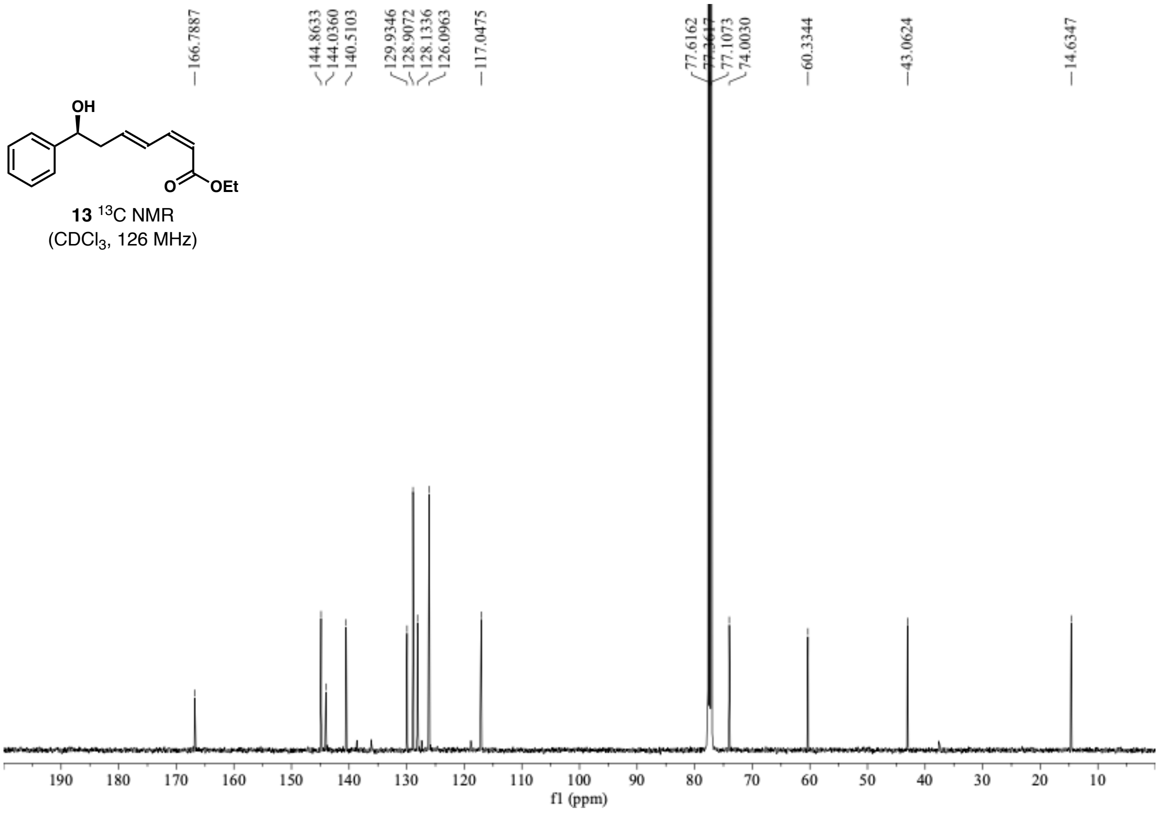
166.7887
144.8633
144.0360
140.5103
129.9346
128.9072
128.1336
126.0963
117.0475
77.6162
77.1073
74.0030
60.3344
43.0624
14.6347
OH
O
OEt
13 13C NMR
(CDCl3, 126 MHz)
f1 (ppm)

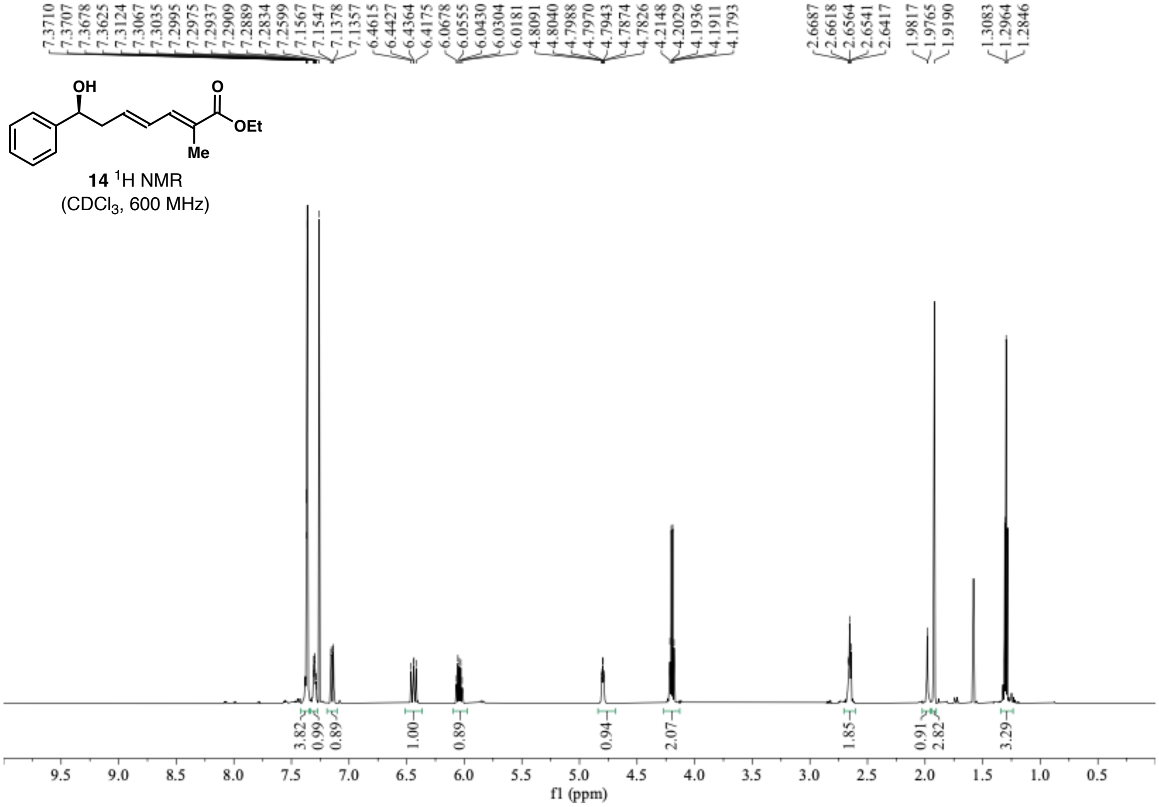
7.3710 7.3707 7.3678 7.3625 7.3124 7.3067 7.3035 7.2995 7.2975 7.2937 7.2909 7.2889 7.2834 7.2599 7.1567 7.1547 7.1378 7.1357 6.4615 6.4427 6.4364 6.4175 6.0678 6.0555 6.0430 6.0304 6.0181 4.8091 4.8040 4.7988 4.7970 4.7943 4.7874 4.7826 4.2148 4.2029 4.1936 4.1911 4.1793 2.6687 2.6618 2.6564 2.6541 2.6417 1.9817 1.9765 1.9190 1.3083 1.2964 1.2846
OH
O
OEt
Me
14 ¹H NMR
(CDCl3, 600 MHz)
3.82 0.99 0.89 1.00 0.89 0.94 2.07 1.85 0.91 2.82 3.29
f1 (ppm)

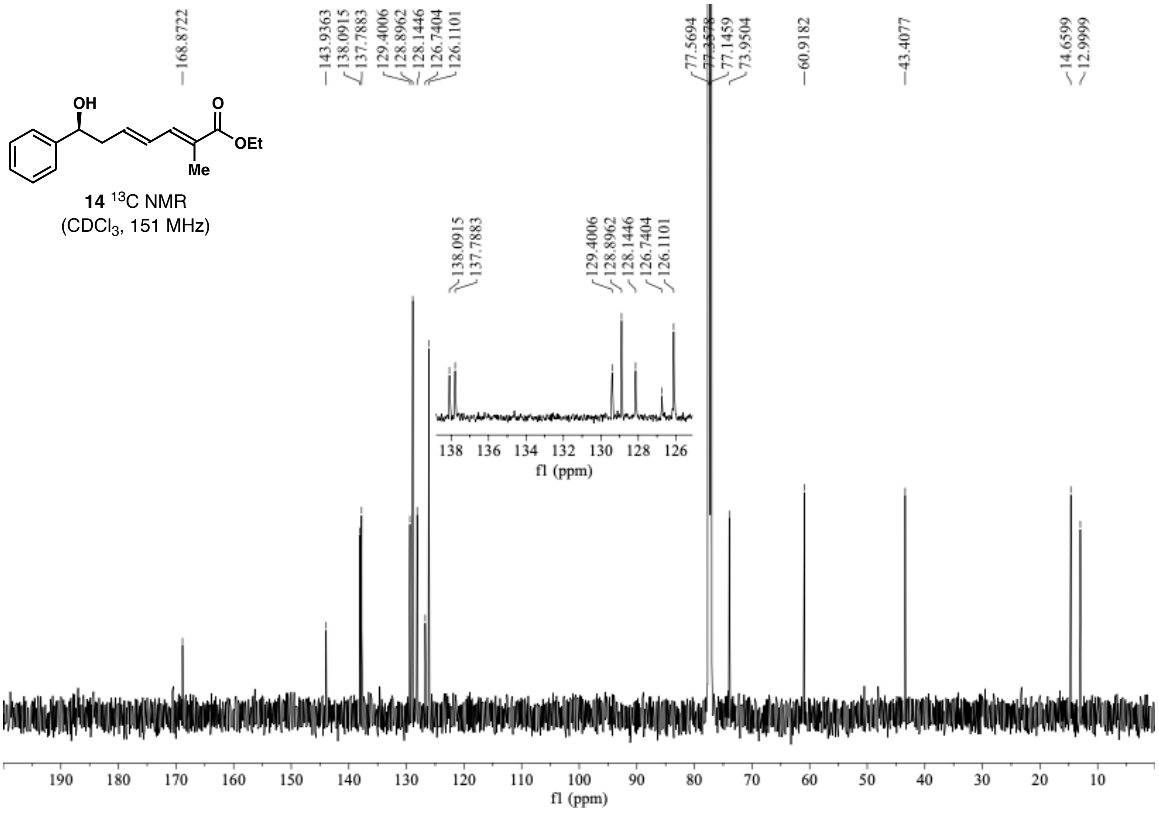
168.8722 143.9363 138.0915 137.7883 129.4006 128.8962 128.1446 126.7404 126.1101 77.5694 77.3578 77.1459 73.9504 60.9182 43.4077 14.6599 12.9999
OH
O
OEt
Me
14 ¹³C NMR
(CDCl3, 151 MHz)
138.0915 137.7883 129.4006 128.8962 128.1446 126.7404 126.1101
f1 (ppm)

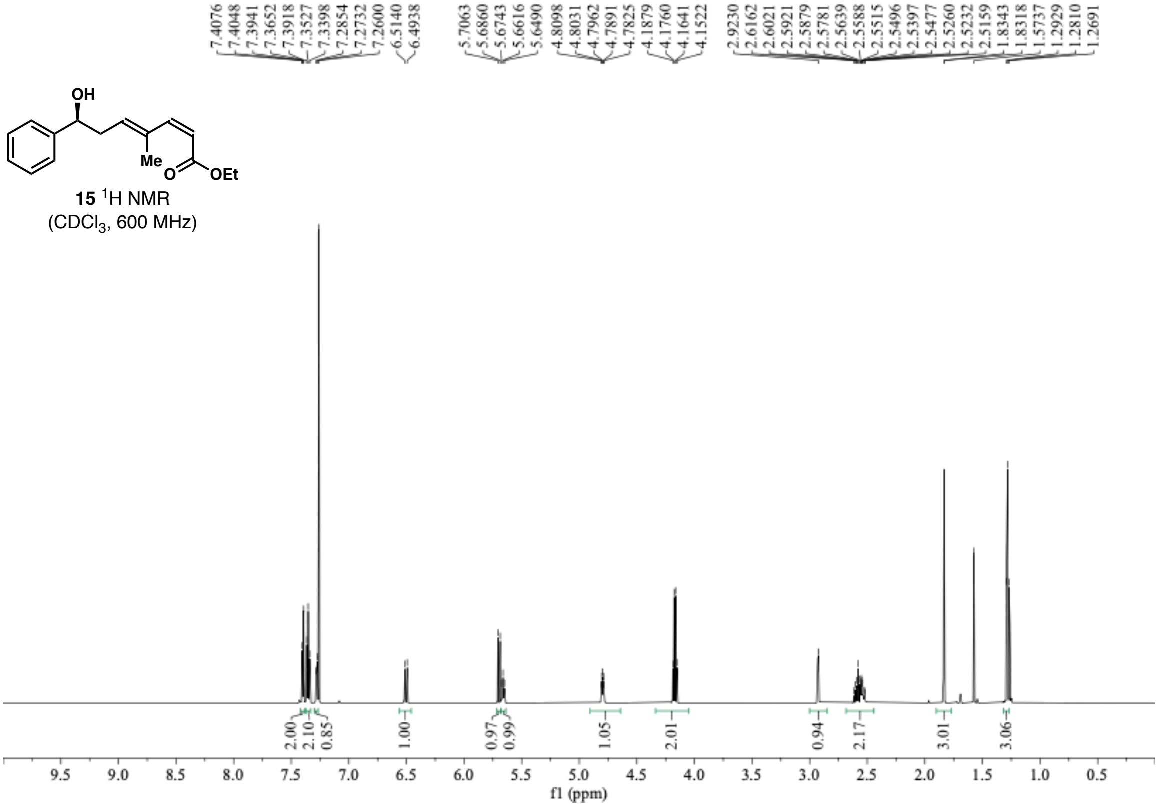

15 ^{1}H NMR
($CDCl_3$, 600 MHz)

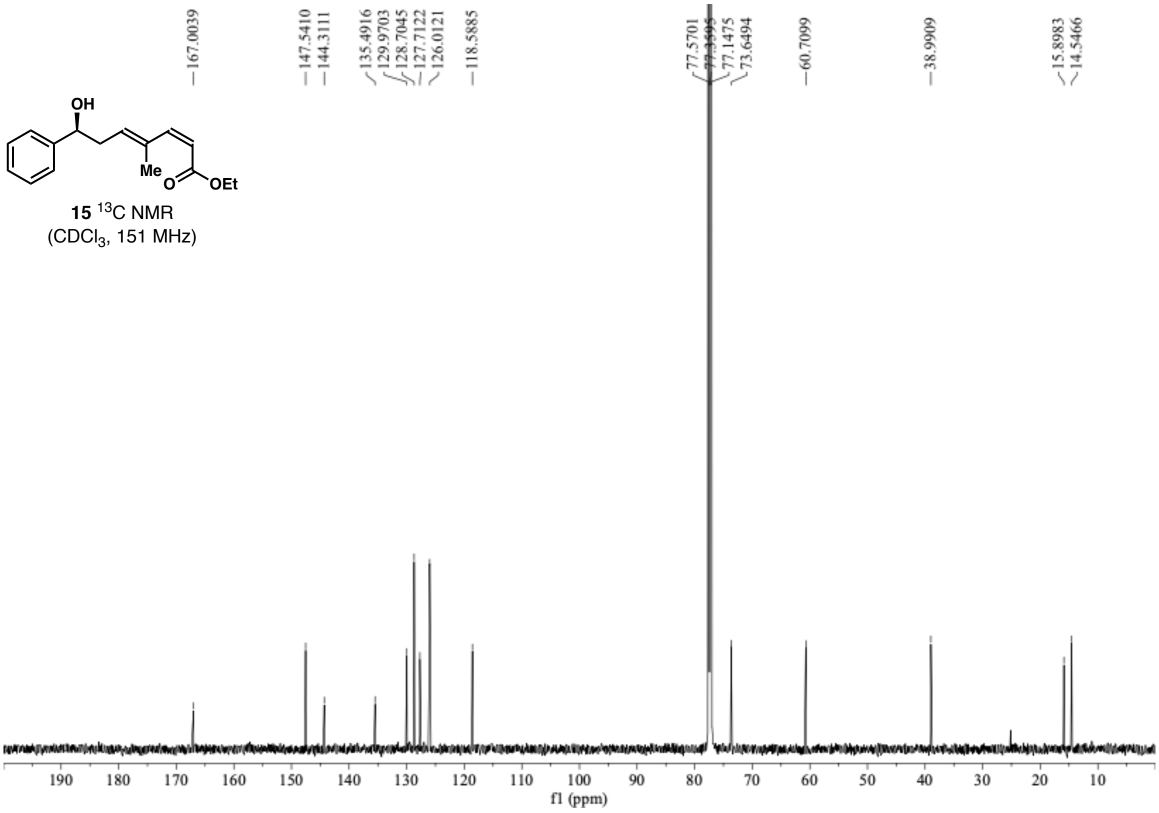

15 ^{13}C NMR
($CDCl_3$, 151 MHz)

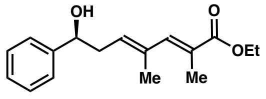

16 ^{1}H NMR
($CDCl_3$, 600 MHz)

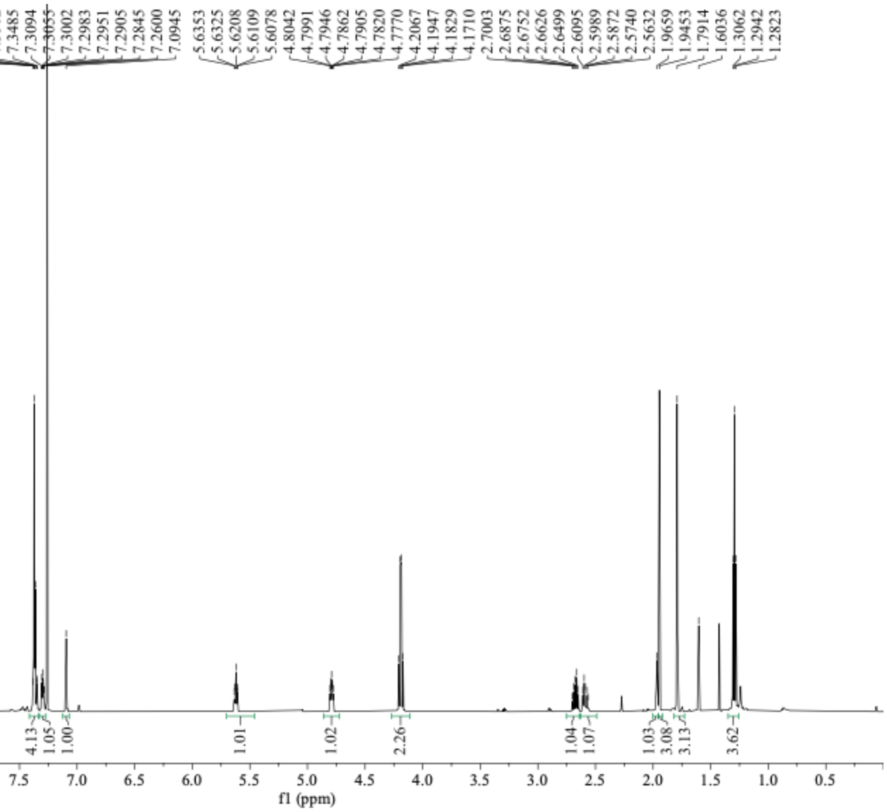

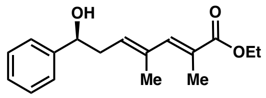

16 ^{13}C NMR
($CDCl_3$, 151 MHz)

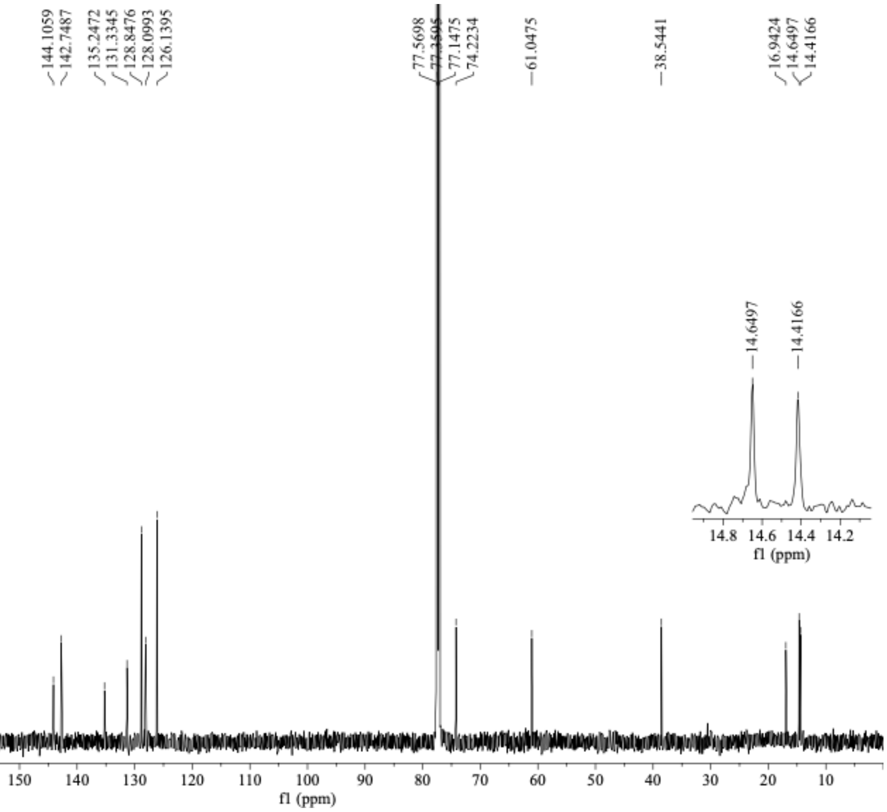

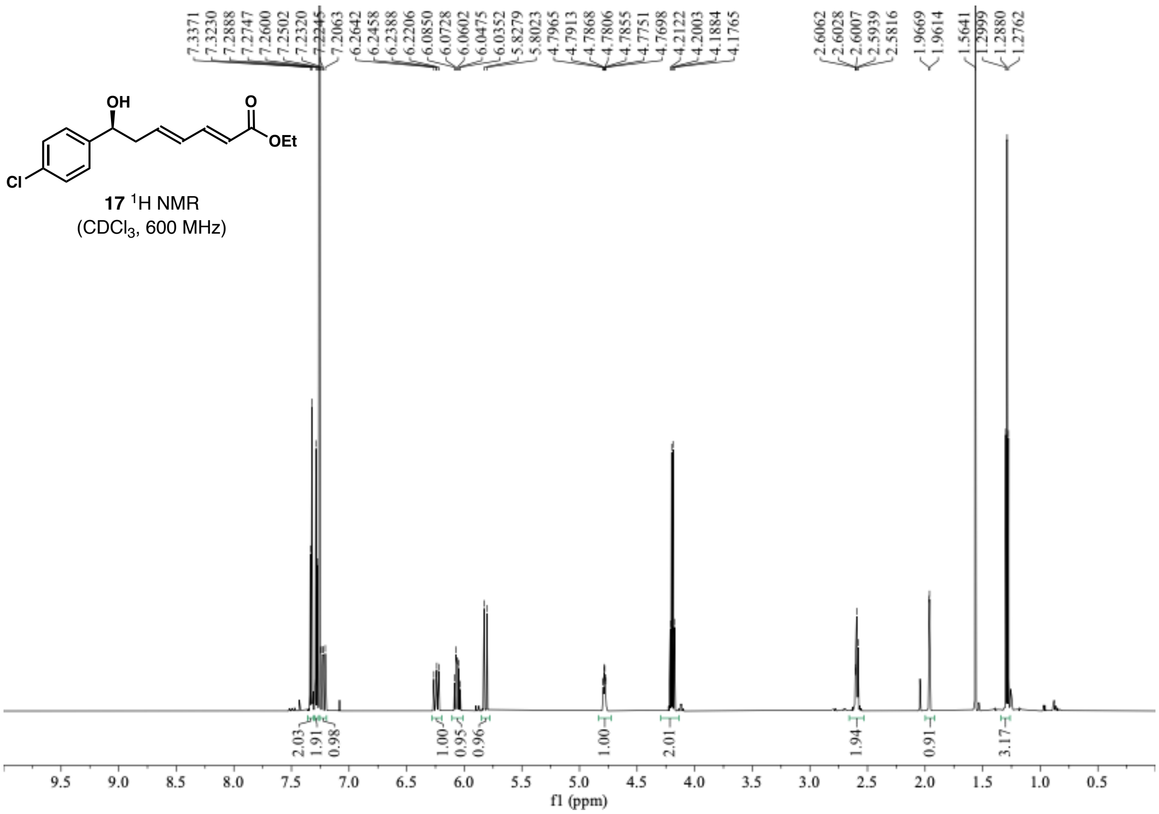
OH
O
OEt
Cl
17 1H NMR
(CDCl3, 600 MHz)
f1 (ppm)

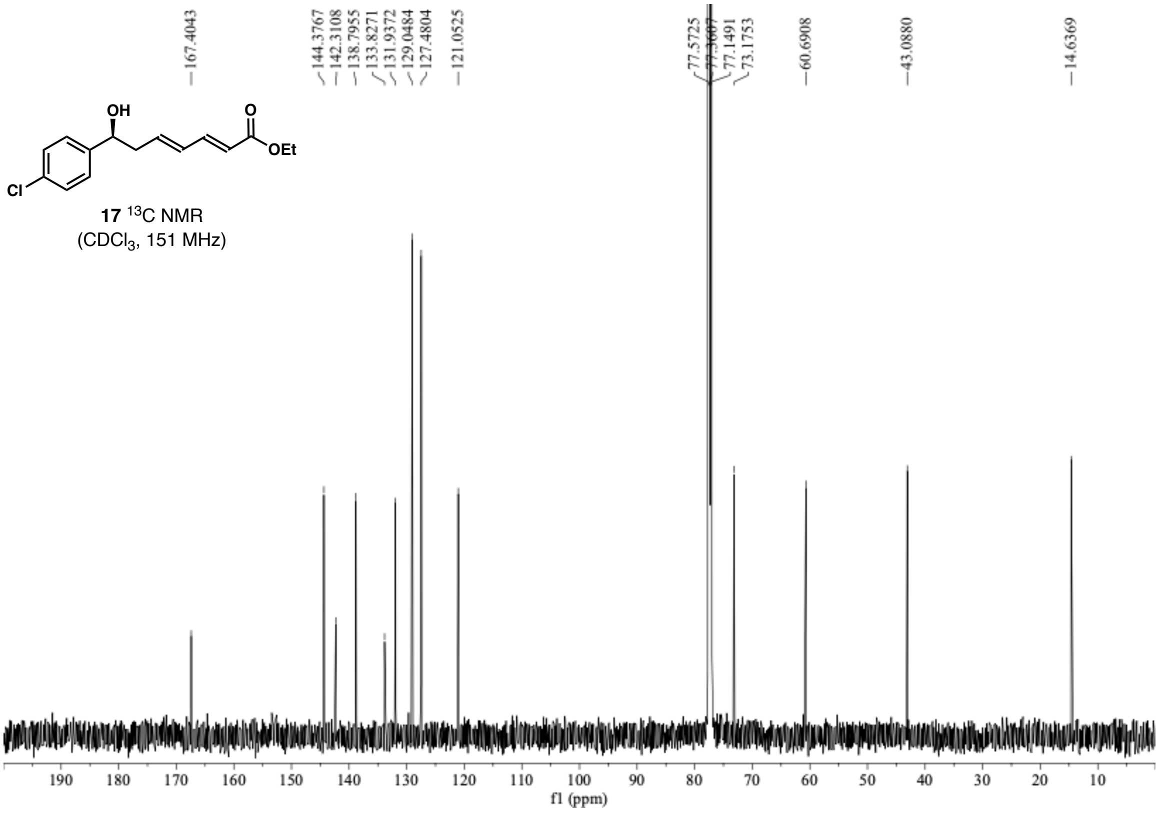
OH
O
OEt
Cl
17 13C NMR
(CDCl3, 151 MHz)
f1 (ppm)

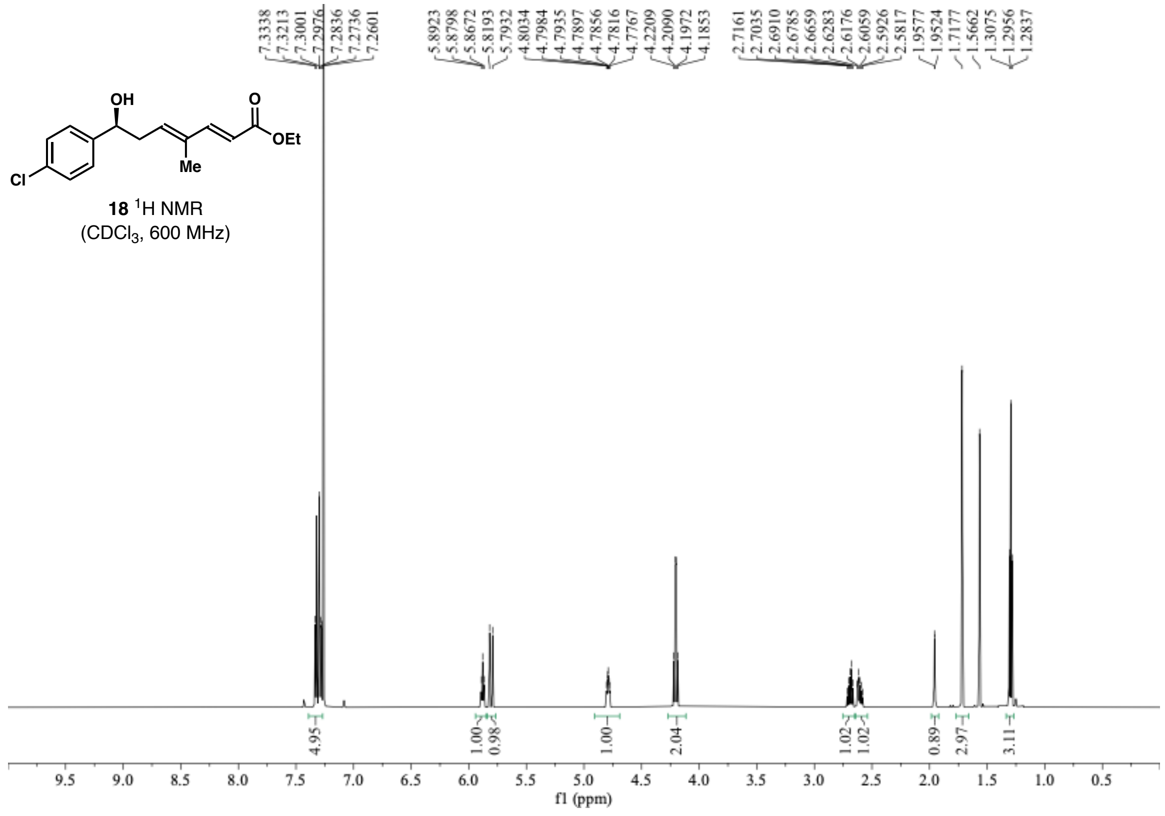

18 ^{1}H NMR
(CDCl$_3$, 600 MHz)
7.3338
7.3213
7.3001
7.2976
7.2836
7.2736
7.2601
5.8923
5.8798
5.8672
5.8193
5.7932
4.8034
4.7984
4.7935
4.7897
4.7856
4.7816
4.7767
4.2209
4.2090
4.1972
4.1853
2.7161
2.7035
2.6910
2.6785
2.6659
2.6283
2.6176
2.6059
2.5926
2.5817
1.9577
1.9524
1.7177
1.5662
1.3075
1.2956
1.2837
4.95
1.00
0.98
1.00
2.04
1.02
1.02
0.89
2.97
3.11
f1 (ppm)

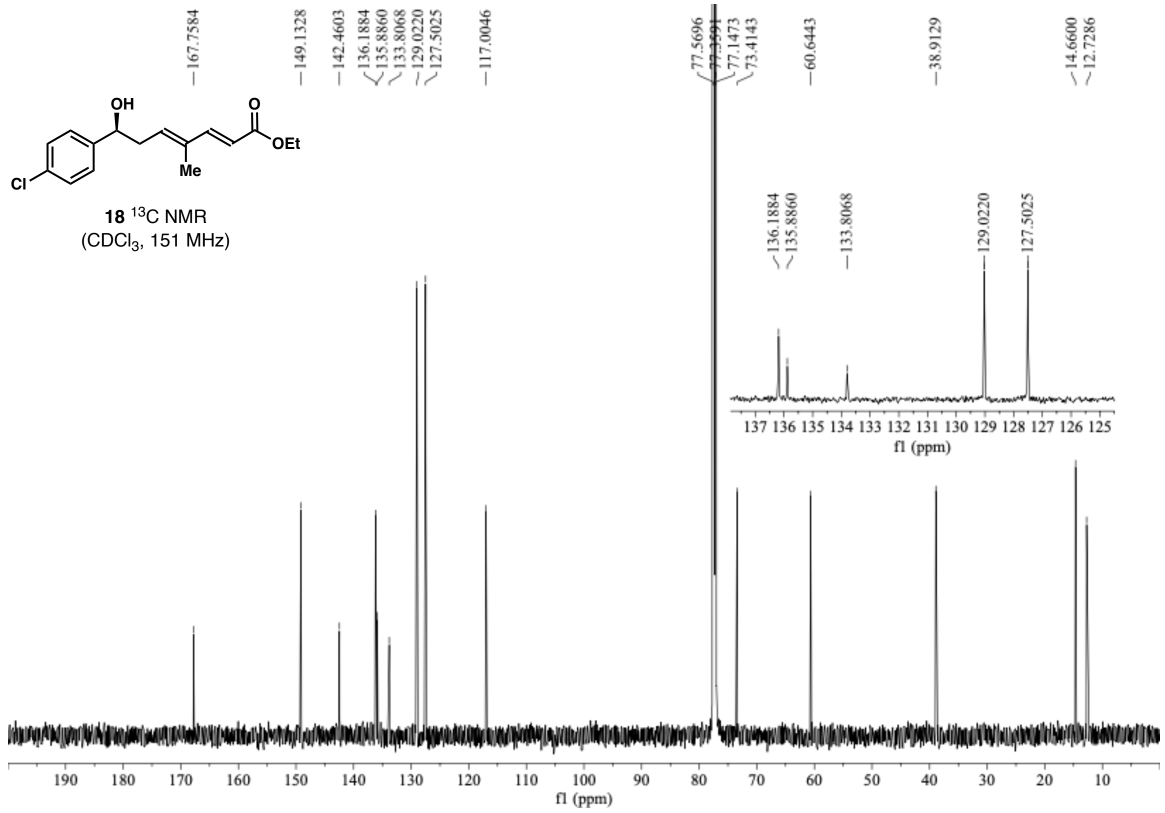

18 ^{13}C NMR
(CDCl$_3$, 151 MHz)
167.7584
149.1328
142.4603
136.1884
135.8860
133.8068
129.0220
127.5025
117.0046
77.5696
77.3591
77.1473
73.4143
60.6443
38.9129
14.6600
12.7286
f1 (ppm)

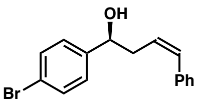

This compound was prepared via Suzuki coupling of **3d** with PhI. Enantiomeric excess was determined by HPLC analysis to be 98% ee (254 nm, 25 ˚C); t_1 = 6.48 min, t_2 = 6.84 min [(Chiralpak IB) hexane/*i*-PrOH, 90:10, 1.0 mL/min]

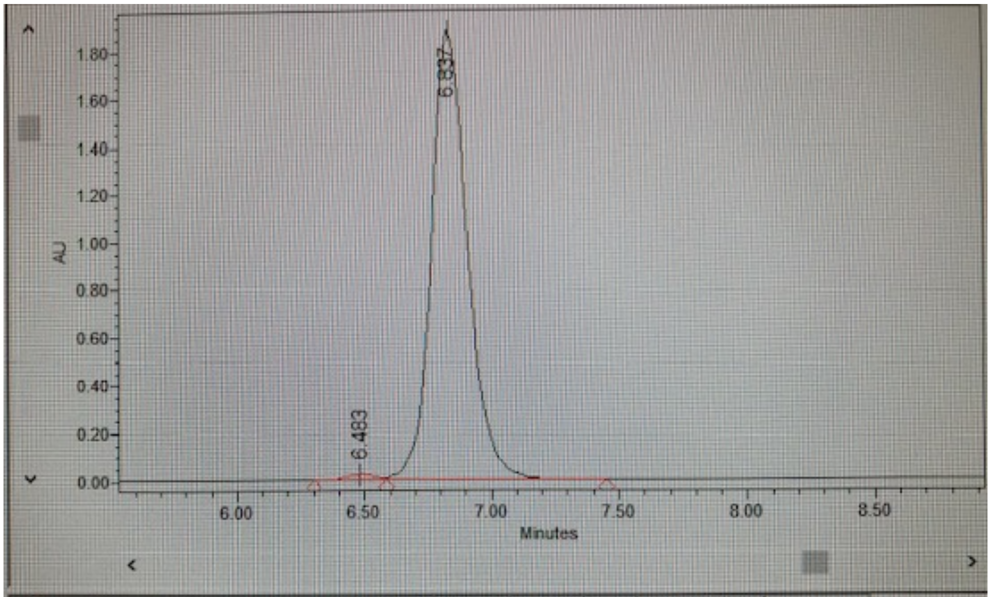

	Name	Retention Time (min)	Area (µV*sec)	% Area	Height (µV)	Int Type	Amount	Units	Peak Type	Peak Codes
1		6.483	223525	1.17	26259	VV			Unknown	
2		6.837	18870676	98.83	1885716	VV			Unknown	

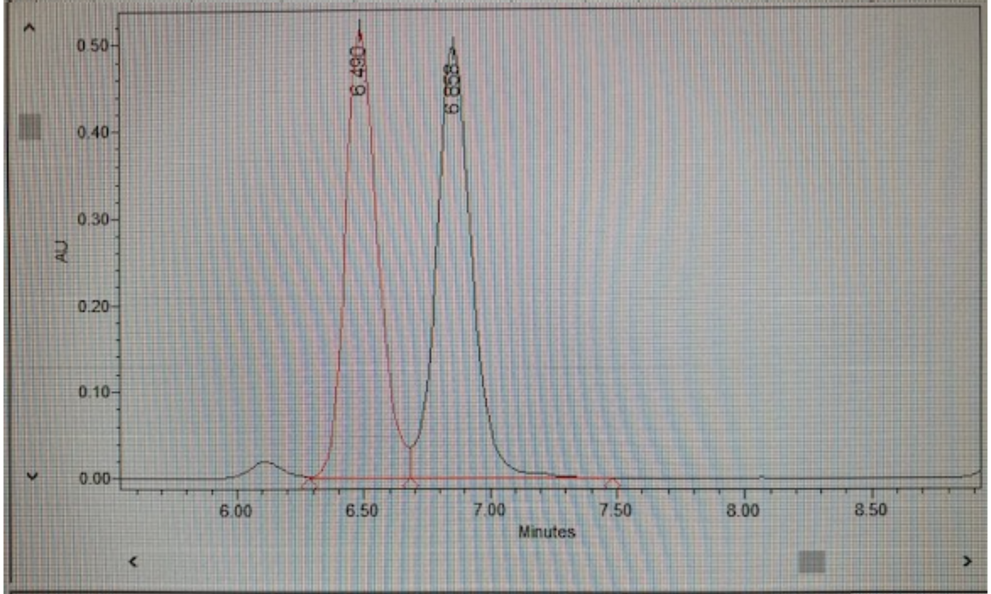

	Name	Retention Time (min)	Area (µV*sec)	% Area	Height (µV)	Int Type	Amount	Units	Peak Type	Peak Codes
1		6.490	4623207	48.69	518144	VV			Unknown	
2		6.858	4871383	51.31	495987	VV			Unknown	

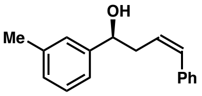

This compound was prepared via Suzuki coupling of **3f** with PhI. Enantiomeric excess was determined by HPLC analysis to be > 99% ee (254 nm, 25 ˚C); t_1 = 10.1 min, t_2 = 11.3 min [(Chiralpak IG) hexane/*i*-PrOH, 95:5, 1.0 mL/min]

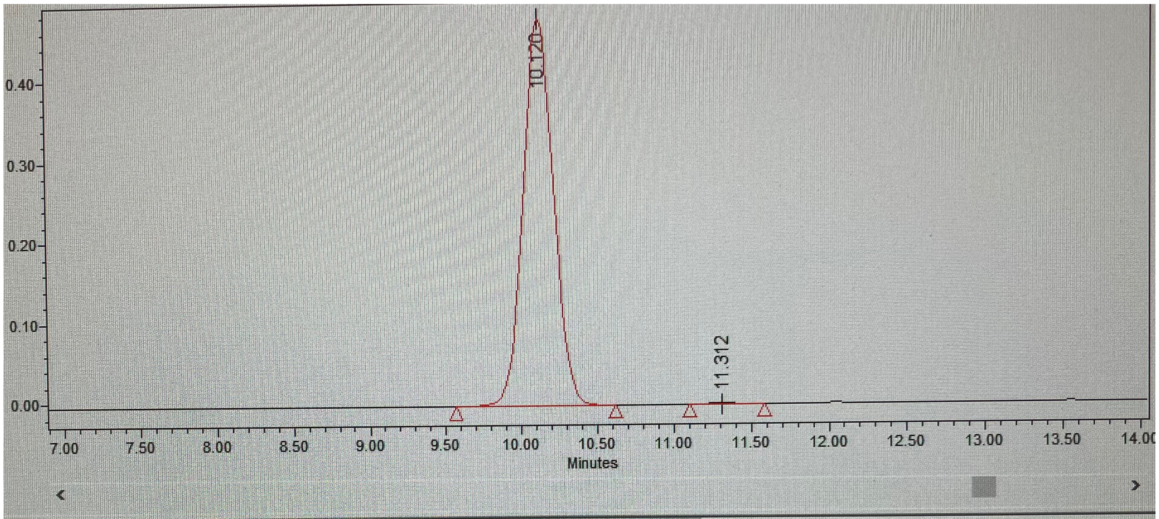

ne	Retention Time (min)	Area (µV*sec)	% Area	Height (µV)	Int Type	Amount	Units	Peak Type	Peak Codes
	10.120	6617374	99.85	476918	BB			Unknown	
	11.312	10000	0.15	636	BB			Unknown	

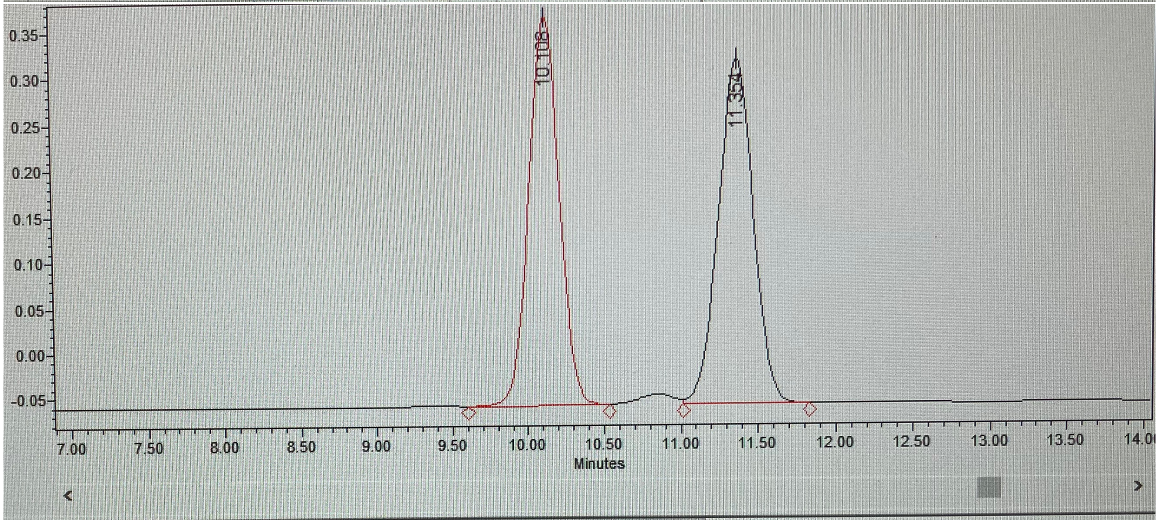

ne	Retention Time (min)	Area (µV*sec)	% Area	Height (µV)	Int Type	Amount	Units	Peak Type	Peak Codes
	10.108	5903020	49.98	423476	VV			Unknown	
	11.354	5907699	50.02	375150	VV			Unknown	

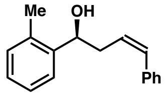

This compound was prepared via Suzuki coupling of **3g** with PhI. Enantiomeric excess was determined by HPLC analysis to be 98% ee (254 nm, 25 ˚C); t_1 = 9.00 min, t_2 = 9.74 min [(Chiralpak IA) hexane/*i*-PrOH, 95:5, 1.0 mL/min];

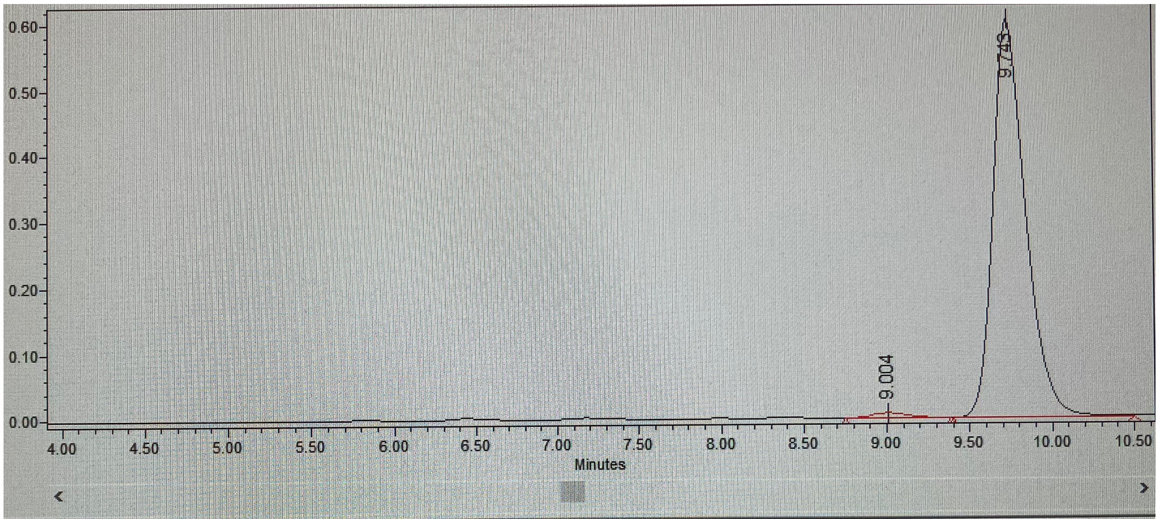

ie	Retention Time (min)	Area (µV*sec)	% Area	Height (µV)	Int Type	Amount	Units	Peak Type	Peak Codes
	9.004	92478	1.10	7078	BB			Unknown	
	9.743	8342150	98.90	604746	BV			Unknown	

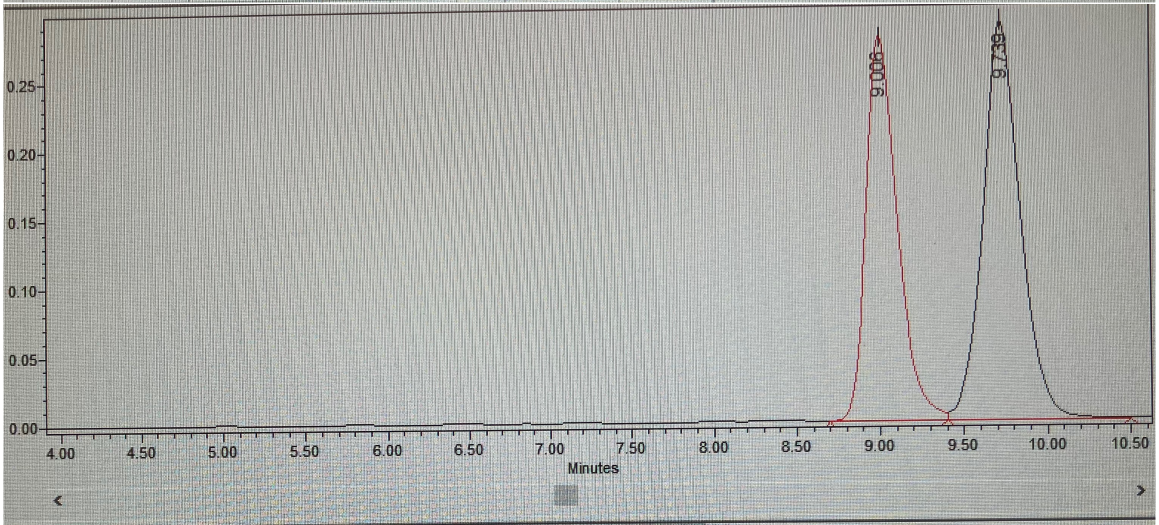

ie	Retention Time (min)	Area (µV*sec)	% Area	Height (µV)	Int Type	Amount	Units	Peak Type	Peak Codes
	9.006	3583455	44.42	277415	BV			Unknown	
	9.739	4483043	55.58	288651	VV			Unknown	

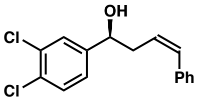

This compound was prepared via Suzuki coupling of **3h** with PhI. Enantiomeric excess was determined by HPLC analysis to be > 99% ee (254 nm, 25 ˚C); t_1 = 6.42 min, t_2 = 6.79 min [(Chiralpak IB) hexane/i-PrOH, 90:10, 1.0 mL/min];

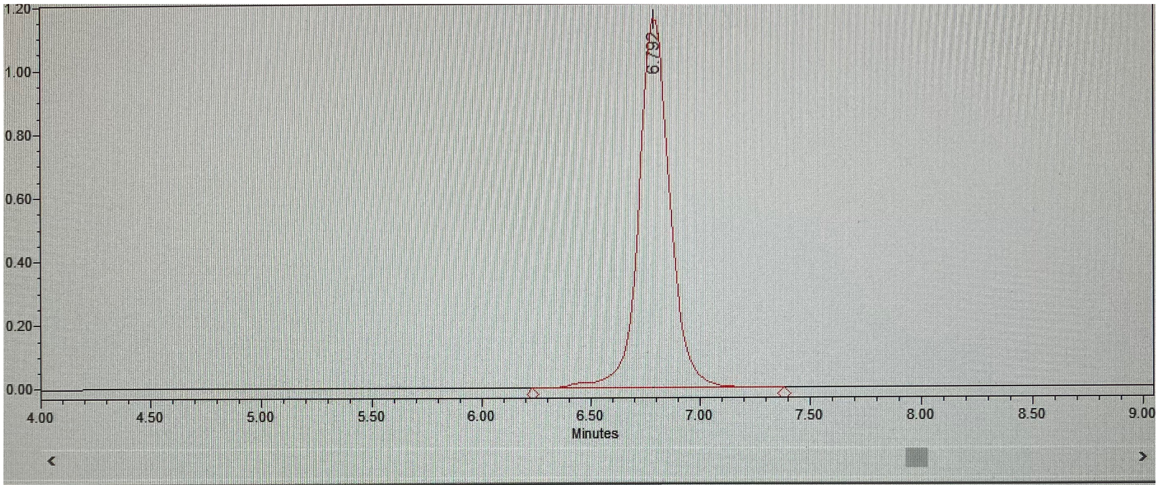

e	Retention Time (min)	Area (µV*sec)	% Area	Height (µV)	Int Type	Amount	Units	Peak Type	Peak Codes
	6.792	11590157	100.00	1160517	VV			Unknown	

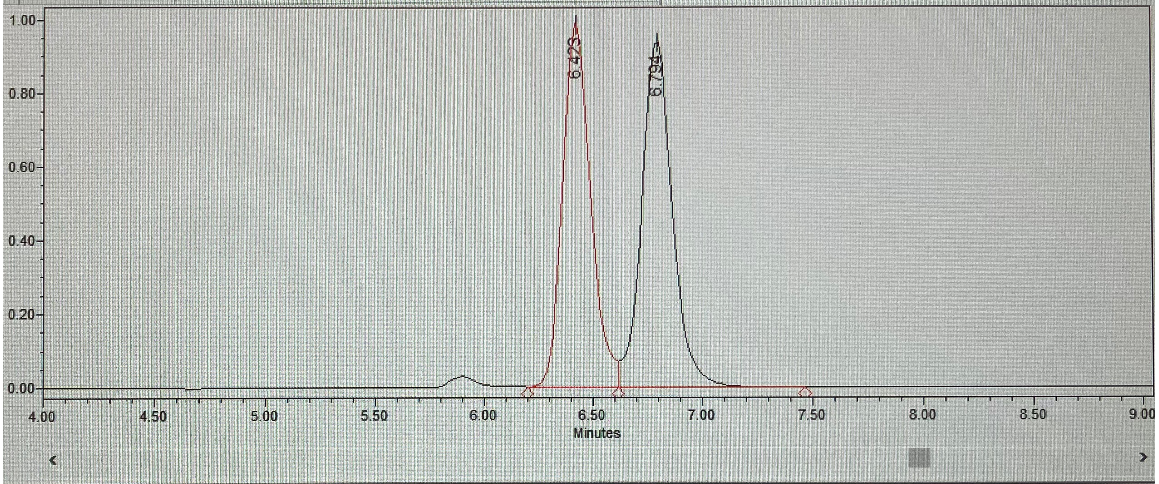

e	Retention Time (min)	Area (µV*sec)	% Area	Height (µV)	Int Type	Amount	Units	Peak Type	Peak Codes
	6.423	8931933	49.08	996296	VV			Unknown	
	6.794	9265286	50.92	945229	VV			Unknown	

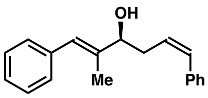

This compound was prepared via Suzuki coupling of **3i** with PhI. Enantiomeric excess was determined by HPLC analysis to be 90% ee (254 nm, 25 °C); t_1 = 10.6 min, t_2 = 11.4 min [(Chiralpak IA) hexane/i-PrOH, 95:5, 1.0 mL/min];

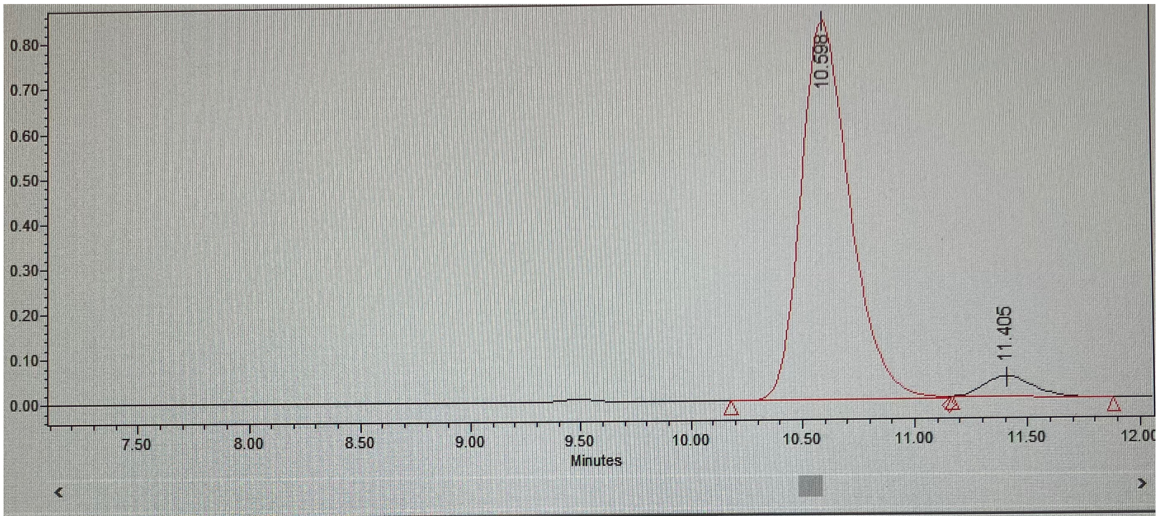

Retention Time (min)	Area (µV*sec)	% Area	Height (µV)	Int Type	Amount	Units	Peak Type	Peak Codes
10.598	12665472	94.96	830874	BV			Unknown	
11.405	672392	5.04	44797	bb			Unknown	

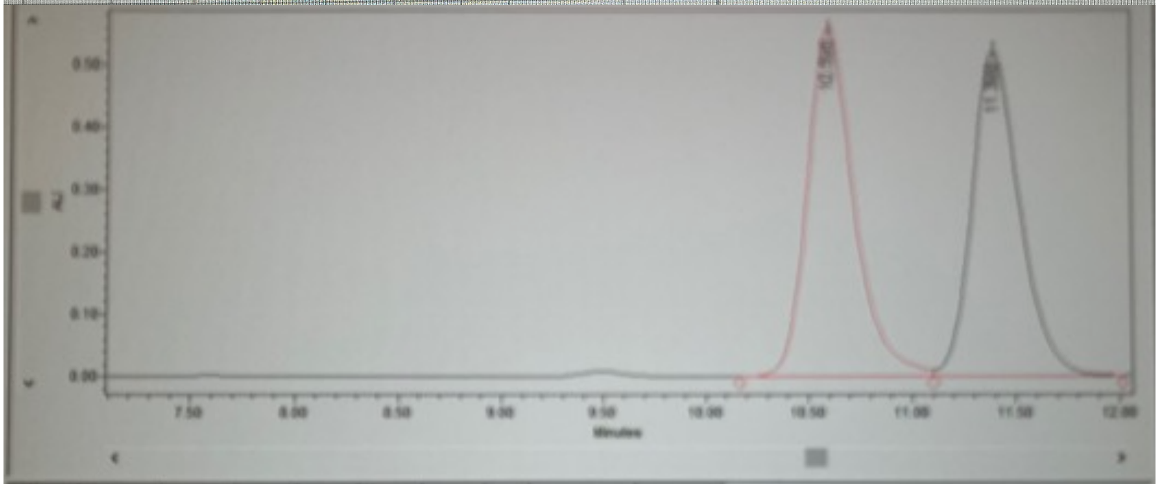

	Name	Retention Time (min)	Area (µV*sec)	% Area	Height (µV)	Int Type	Amount	Units	Peak Type	Peak Codes
1		10.595	8706483	51.16	581179	VV			Unknown	
2		11.399	8388449	48.84	522554	VV			Unknown	

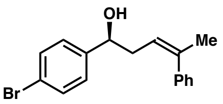

This compound was prepared via Suzuki coupling of **3m** with PhI. Enantiomeric excess was determined by HPLC analysis to be > 99% ee (254 nm, 25 ˚C); t_1 = 9.83 min, t_2 = 10.3 min [(Chiralpak IA) hexane/i-PrOH, 95:5, 1.0 mL/min]

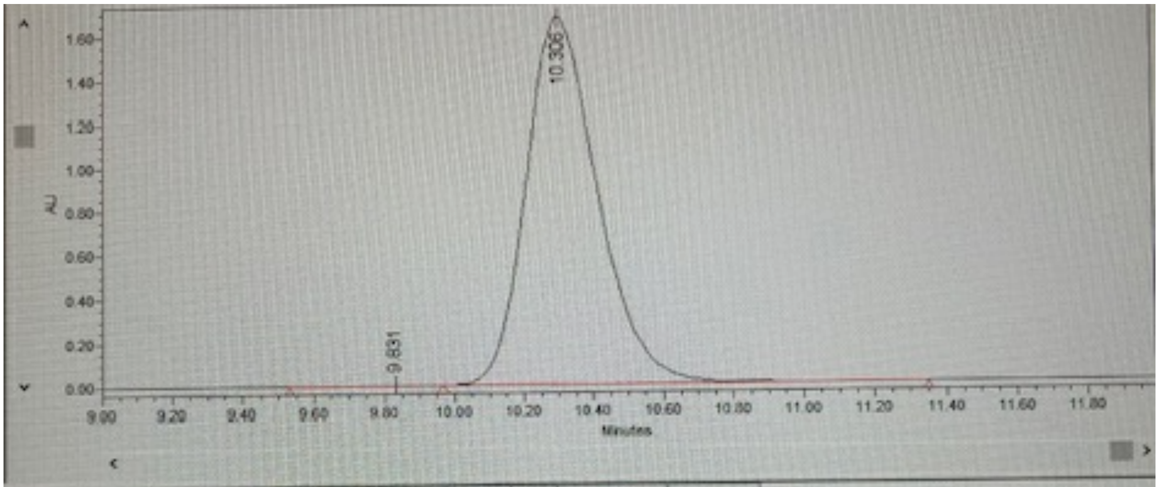

	Name	Retention Time (min)	Area (µV*sec)	% Area	Height (µV)	Int Type	Amount	Units	Peak Type	Peak Codes
1		9.831	53688	0.22	4432	BV			Unknown	
2		10.306	24087144	99.78	1671841	VB			Unknown	

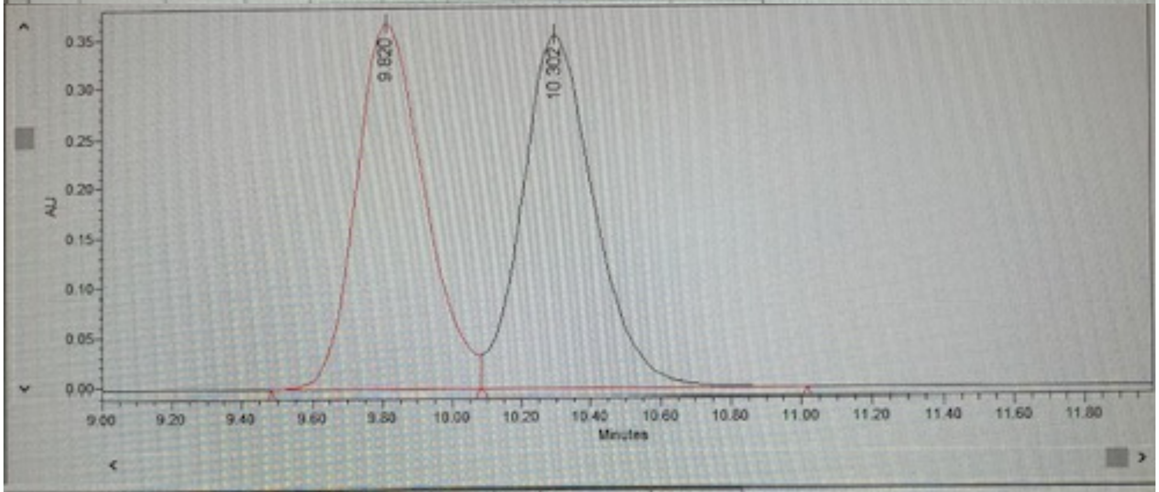

	Name	Retention Time (min)	Area (µV*sec)	% Area	Height (µV)	Int Type	Amount	Units	Peak Type	Peak Codes
1		9.820	4990691	49.29	367294	BV			Unknown	
2		10.302	5133645	50.71	355186	VB			Unknown	

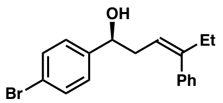

This compound was prepared via Suzuki coupling of **3n** with PhI. Enantiomeric excess was determined by HPLC analysis to be > 99% ee (254 nm, 25 ˚C); t_1 = 8.77 min, t_2 = 9.57 min [(Chiralpak IA) hexane/i-PrOH, 95:5, 1.0 mL/min]

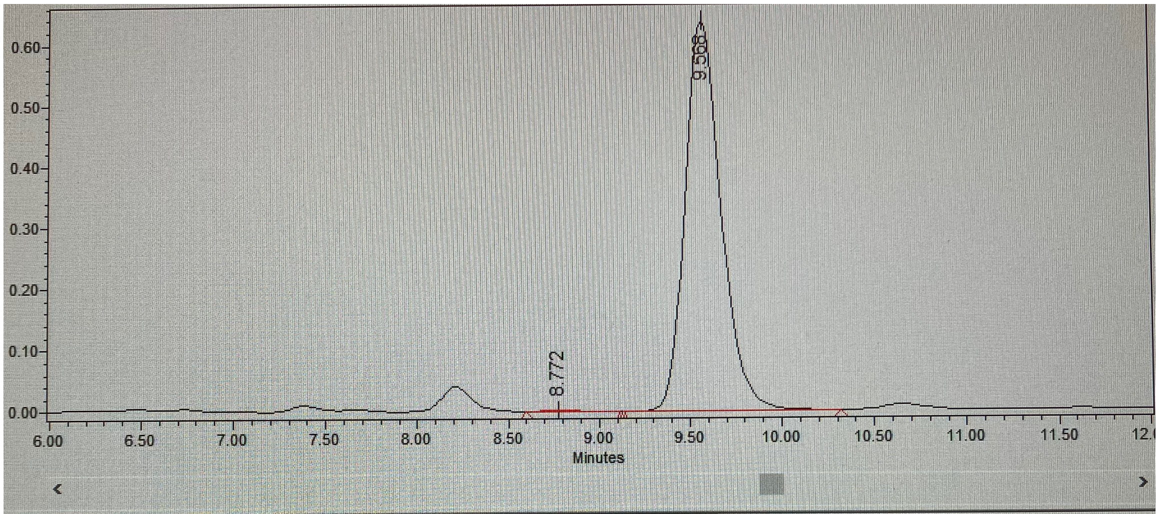

Retention Time (min)	Area (μV*sec)	% Area	Height (μV)	Int Type	Amount	Units	Peak Type	Peak Codes
8.772	27946	0.32	2114	VB			Unknown	
9.568	8696300	99.68	635594	BV			Unknown	

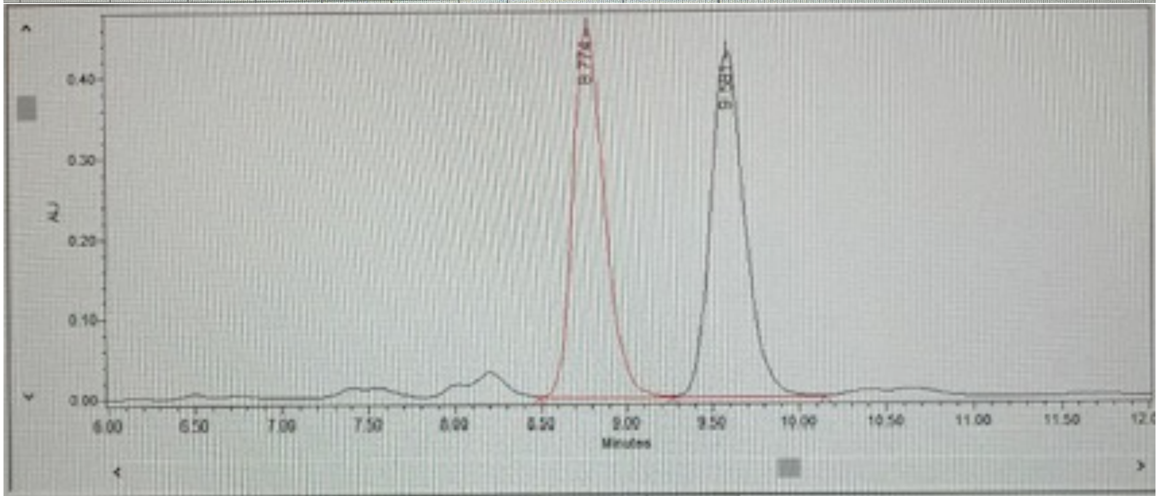

	Name	Retention Time (min)	Area (μV*sec)	% Area	Height (μV)	Int Type	Amount	Units	Peak Type	Peak Codes
1		8.774	5922455	49.94	461960	VV			Unknown	
2		9.581	5936110	50.06	429999	VV			Unknown	

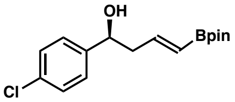

Enantiomeric excess of **2d** was determined by HPLC analysis to be 97% ee (254 nm, 25 ˚C); t_1 = 8.48 min, t_2 = 9.77 min [(Chiralpak IA) hexane/i-PrOH, 95:5, 1.0 mL/min]

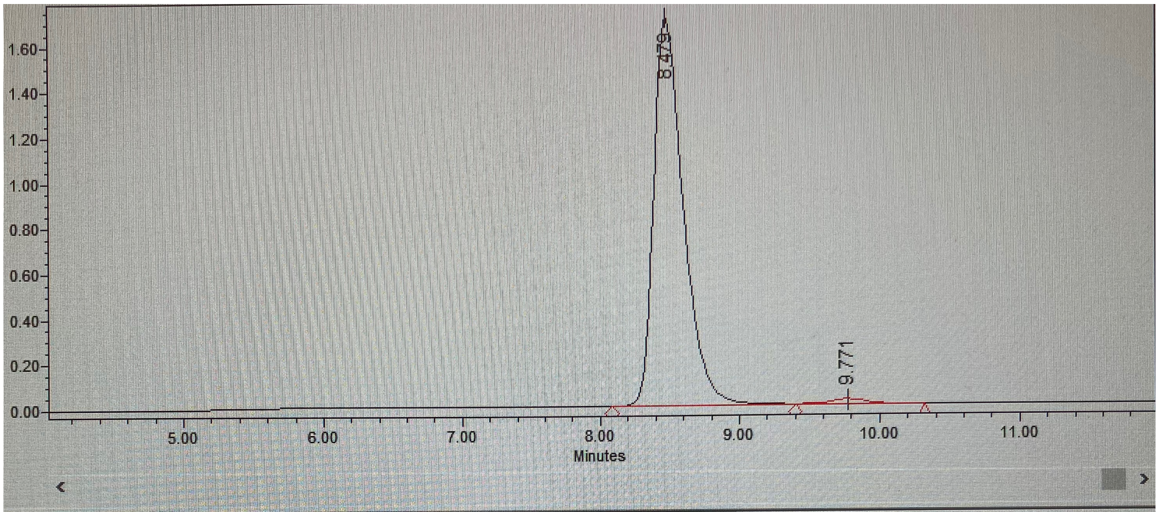

Retention Time (min)	Area (µV*sec)	% Area	Height (µV)	Int Type	Amount	Units	Peak Type	Peak Codes
8.479	25172375	98.35	1697946	VV			Unknown	
9.771	421615	1.65	22716	VB			Unknown	

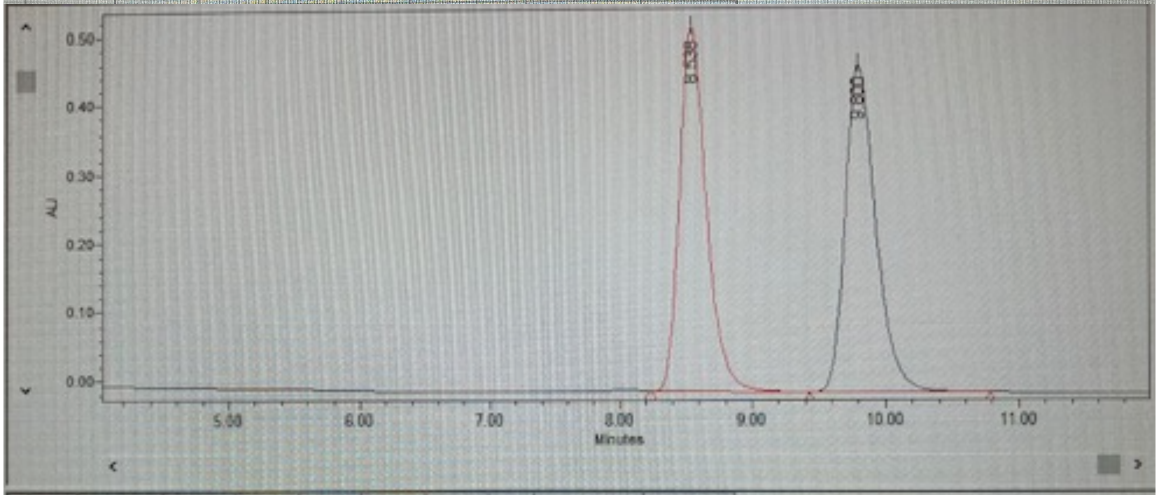

	Name	Retention Time (min)	Area (µV*sec)	% Area	Height (µV)	Int Type	Amount	Units	Peak Type	Peak Codes
1		8.538	7492862	49.94	527423	VB			Unknown	
2		9.800	7509990	50.06	472564	BB			Unknown	
